# Molecular Design of Sexiphenyl-Based Liquid Crystals: Towards Temperature-Stable, Nematic Phases with Enhanced Optical Properties

**DOI:** 10.3390/molecules29050946

**Published:** 2024-02-21

**Authors:** Jakub Herman, Piotr Harmata, Natan Rychłowicz, Przemysław Kula

**Affiliations:** Faculty of Advanced Technologies and Chemistry, Military University of Technology, 2 Gen. S. Kaliskiego St., 00-908 Warsaw, Poland; jakub.herman@wat.edu.pl (J.H.); piotr.harmata@wat.edu.pl (P.H.); natan.rychlowicz@wat.edu.pl (N.R.)

**Keywords:** sexiphenyl, nematic, birefringence, high chemical stability

## Abstract

This research introduces a novel liquid crystal molecular design approach based on the para-sexiphenyl (6P) structure. Six new liquid crystalline materials were synthesized, incorporating an alkyl terminal and lateral substitutions of the sexiphenyl core to achieve temperature-stable and broad nematic phases. The synthetic pathway involved cross-coupling, resulting in derivatives with strong nematogenic characteristics. Optical investigations demonstrated that the tested material had high birefringence values, making it promising for optical and electronic applications. These results open up new avenues of research and offer potential practical applications in electronics, photonics, optoelectronics and beyond.

## 1. Introduction

Para-Sexiphenyl (6P) is a type of organic material that has been extensively studied in the field of materials science due to its unique properties and potential applications in various electronic and optoelectronic devices [1,2,3]. p-Sexiphenyl molecules are composed of six phenyl rings that are linked together by a single bond, resulting in a rigid structure. This arrangement gives rise to strong intermolecular interactions and an extended pi-electron system, which leads to high charge carrier mobility and luminescence efficiency [4]. In recent years, sexiphenyl organic materials have been explored as promising materials for lasers [5,6] and organic thin film transistors (OTFTs) [7,8,9,10]. The high electron mobility of sexiphenyl enables efficient charge transport in OFETs [1], while its high luminescence efficiency makes it an attractive choice for organic diodes [11,12,13,14,15,16]. Additionally, the unique electronic properties of sexiphenyl have been harnessed to create high-performance OPVs with good stability and durability [6,17,18]. In addition to their technological applications, sexiphenyl organic materials are of interest to researchers studying the physics and chemistry of complex materials. Through functionalization or blending with other organic or inorganic materials, they have the potential to be used as hybrid materials in the development of high-performance electronic devices [19,20,21,22,23], as well as in the development of sensors with high sensitivity and selectivity [24,25]. One of the most interesting properties of sexiphenyl materials is their ability to self-assemble, which further creates liquid crystal behavior. Sexiphenyl materials (sometimes referred to as hexaphenyls) have gathered much attention in the field of materials science for their ability to form temperature-stable, nematic liquid crystalline phases with highly anisotropic properties [26,27,28,29,30,31,32,33]. These materials are characterized by their rigid, elongated core consisting of multiple aromatic rings, and they are mainly known for their high thermal and photochemical stability. Sexiphenyl organic materials individually form temperature-stable liquid crystalline phases; however, the use of these materials as dopants in nematic liquid crystal mixtures has also been explored, leading to enhanced nematic stability and electro-optical properties [29,34]. Nevertheless, such materials have interesting liquid crystalline properties, but at elevated temperatures, which are typical of oligophenyls that are strongly elongated by aromatic rings. Therefore, research is currently focused on the element of designing and obtaining new organic materials with enhanced properties, such as improved solubility, thermal stability and even photoluminescence. Overall, the combination of the sexiphenyl structure with liquid crystal behavior represents an exciting area of research with significant potential for practical applications. Continued research into these materials is likely to uncover new and innovative structures and uses for them in a range of fields, from electronics and photonics, even to biomedicine and beyond. 

**Figure 1:**
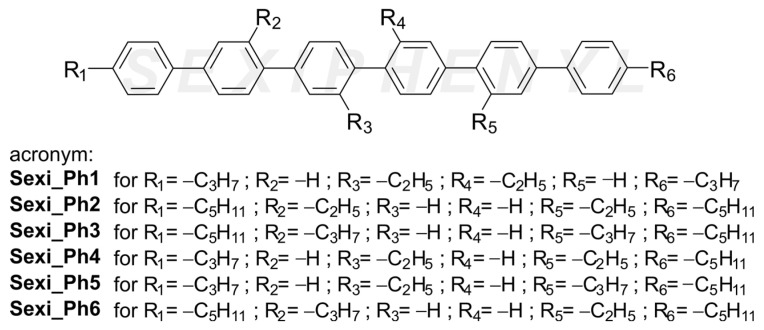
The general structure of the investigated materials.

In this work, we present our approach to a new liquid crystal molecular design, which is based on the sexiphenyl (6P) structure—see the general structure in Figure 1. New materials show a tendency to form temperature-stable and broad nematic phases. In contrast to already developed and investigated 6P materials, alkyl terminals and the lateral substitution of sexiphenyl cores bring the temperatures of phase transitions to much lower levels, which are within reach of many modern experiments. Our findings demonstrate that the incorporation of side groups in a planar mesogenic structure broadens the molecule, thus inducing a separation of the molecular axes. This phenomenon results in diminished intermolecular interaction and ultimately leads to a decrease in the transition temperature, far below 200 °C [29,30,31]. Here, we use the expression temperature-stable nematic, which refers to the ability of a liquid crystal system to maintain constant mesomorphic (here nematic) characteristics over a very wide temperature range.

## 2. Results

### 2.1. Synthesis

**Figure 2:**
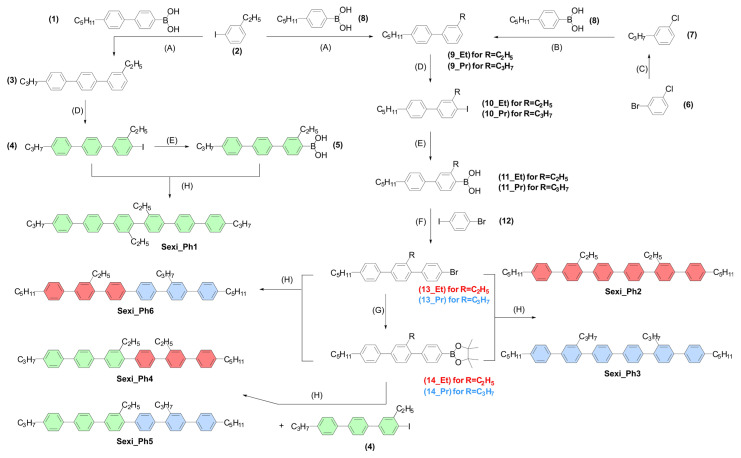
Synthetic pathway to sexiphenyl materials. Reaction condition: (**A**) Pd(OAc)_2_, K_2_CO_3_, acetone, H_2_O; (**B**) Pd(OAc)_2_, SPhos, K_3_PO_4_·3H_2_O, THF; (**C**) i. Mg, THF, ii. C_3_H_7_Br, Li_2_CuCl_4_, THF; (**D**) KIO_3_, I_2_, H_2_SO_4_, CH_3_COOH; (**E**) tert-BuLi, then B(OPr)_3_, then HCl(aq); (**F**) PdCl_2_(PPh_3_)_2_, K_3_PO_4_·3H_2_O, toluene; (**G**) Pd_2_(dba)_3_, B_2_pin_2_, Cs_2_CO_3_, toluene; (**H**) Pd(OAc)_2_, Cs_2_CO_3_, DME, H_2_O.

The methodology for the synthesis of sexiphenyl liquid crystalline materials was based on the Suzuki–Miyaura coupling protocol. The appropriate selection of reactants successively forming the polyaromatic core and their sequence of use was planned for all organic materials. Materials were terminally ended with standard alkyl chains and additionally laterally substituted with short alkyl chains. Such a move aimed at lowering the temperatures of phase transitions and allowed us to obtain the nematic phase in a temperature range much lower than that for previously studied materials of this type [29,30,31,35]. The synthetic path is presented in Figure 2.

The synthetic procedure of p-sexiphenyls was divided into several subsections. In general, the idea of synthesizing such structures was based on the best available tool for combining benzene rings, i.e., the Suzuki–Miyaura cross-coupling reaction. The gradual growth of successive aromatic units forming the liquid crystalline core required the generation of suitable multi-ring halogen derivatives and suitable multi-ring boronic acid derivatives or boronic ester derivatives. Depending on the stage of synthesis (i.e., the degree of expansion of the aromatic core), the conditions for conducting the coupling reaction themselves were also modified. Everything depended primarily on the degree of solubility of the reactants used and the products formed in the reaction. The greatest degree of difficulty in the presented methodology turned out to be the proper selection of solvents for the individual steps of the multi-step procedure, to ensure high conversion, which was directly related to the solubility of the organic materials mentioned. It turned out that ether-type solvents (1,2-dimethoxyethane, tetrahydrofuran) were the best choice, generating the highest solubility, and thus the degree of cross-coupling, which significantly raised the process temperature while maintaining the very good solubility of both reactants and products. The synthesis strategy presented in Figure 2 involved the production of terphenylene intermediates, which formed a six-ring core in the coupling reaction in the final step. The problem of the formation of six-ring by-products of homocoupling processes was eliminated by using cyclic boronic esters (dioxaborolane derivatives). The final products of the synthesis were purified to a very high level (a minimum of 99.5% LC) using a multiple sequence of recrystallization processes from toluene and column chromatography (dichloromethane + silica gel). General synthetic procedures are gathered in ESI.

### 2.2. Mesomorphic Properties

The individual structures differed in both the position of placement of lateral substituents in the core and their length. In addition, the series included sexiphenyls that differed in the length of the terminal chains on either side of the core. Each compound obtained had its corresponding acronym (**Sexi_PhX**, where X = 1–6). Table 1 shows the exact temperatures and enthalpies of the phase transitions. 

**Figure 3:**
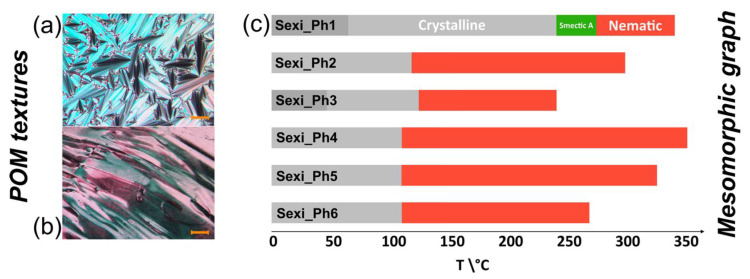
POM textures of **Sexi_Ph1**: (**a**) focal-conic texture of Smectic A phase at 250 °C on cooling; (**b**) nematic phase at 320 °C on cooling, scale bars correspond to 20 μm; (**c**) graphical presentation of the mesomorphic characteristic of sexiphenyls; temperatures obtained from the first cooling cycle.

Each of the sexiphenyl compounds obtained had a nematic phase. For the compounds **Sexi_Ph2**–**Sexi_Ph6**, the study excluded the presence of smectic phases. The exception was the symmetrical derivative **Sexi_Ph1**, for which a monotropic smectic SmA phase was observed, verified on POM with characteristic focal-conic textures—see Figure 3. The smectic phase was not studied in detail. Some of investigated materials also showed crystalline–crystalline transitions, which were not investigated in detail here. For compounds with isotropization temperatures above 300 °C, no thermal decomposition of the samples tested was observed. Among the characterized compounds, the widest temperature range of the nematic phase, together with the lowest melting point, was exhibited by the compound **Sexi_Ph4**. However, the determination of structure–property-type correlations between the individual compounds was problematic due to their high structural diversity. Among the compounds labeled with the acronyms **Sexi_Ph2**–**Sexi_Ph6**, the temperatures of crystal–nematic transition were in a similar range of around 150 °C. On the other hand, depending on the substitution, they differed significantly in the values of isotropization temperatures. Thus, compounds that had different lengths of both terminal and lateral chains were characterized by the highest T_N-Iso_ temperatures (significantly above 300 °C), and the temperature range of the nematic phase exceeded 150 Kelvins—see structure **Sexi_Ph4** and **Sexi_Ph5**. The exception here was the material **Sexi_Ph1**, which very strongly deviated in characteristics from all others. Materials with pentyl terminal chains, in particular **Sexi_Ph3** and **Sexi_Ph6,** already had lower T_N-Iso_, while still maintaining a large nematic phase range (90–120 K). 

### 2.3. Optical Properties

The basic optical properties were also investigated for the selected compounds **Sexi_Ph4**, **Sexi_Ph5**, and **Sexi_Ph6**. As the liquid crystalline compounds under study are not nematic at room temperature, their properties had to be extrapolated from the low birefringent nematic base mixture. For this purpose, the test compound was added to the nematic base mixture in amounts of 2%, 6%, 14%, and 17% by weight. The values of refractive indices and birefringence for the pure liquid crystalline compound were determined by extrapolation from Equation (1), as follows:

(∆n)_gh_ = x(∆n)_g_ + (1 − x)(∆n)_h_, (1)

where ∆n is the birefringence value; gh—the test mixture; g—the base mixture; h—the test compound; and x is the concentration of the test compound added to the base mixture given in molar fractions. 

The nematic matrix is a ternary mixture of compounds from the 4n-alkyloxyphenyl trans-4n-cyclohexylcarboxylate family—see Table 2. 

It has the following properties: isotropization temperature T_N-Iso_ = 63.6 °C; melting point T_Cr-N_ < −20 °C; viscosity η = 21.2 mPa·s; and birefringence Δn = 0.07 measured at T = 22 °C for 636 nm. Table 3 presents the optical properties of three compounds (**Sexi_Ph4**, **Sexi_Ph5**, and **Sexi_Ph6**) at three different wavelengths (443 nm, 636 nm, and 1550 nm). Unfortunately, the solubility of the remaining compounds (**Sexi_Ph1**, **Sexi_Ph2**, and **Sexi_Ph3**) in the low birefringent nematic base was too low (maximum 3%), so the determination of their optical parameters was subject to too much error. The optical parameters include the refractive indices (n_o_ and n_e_) and the birefringence (Δn). For all compounds tested, the refractive indices exhibited a decreasing trend as the wavelength increased, with the birefringence also following this pattern. **Sexi_Ph5** maintains the correct trend, with slightly higher n_o_ values compared to **Sexi_Ph4** at each wavelength. **Sexi_Ph6** also followed the general trend as well, with the lowest n_e_ values among the three compounds at each wavelength. Notably, despite variations in the substitution of the sexiphenyl core, the refractive indices (n_o_ and n_e_) and birefringence (Δn) showed very similar values of refractive indices and birefringence at all wavelengths, and their change was small. This suggests that changing the length of the alkyl lateral substituent and changing the length of the alkyl terminal substituent of the sexiphenyl core does not significantly change the optical properties, emphasizing the robust and stable behavior. It is the rigid aromatic core rich in π-electrons that has the greatest impact on the birefringence value here. Theoretical studies were employed to quantify the influence of alkyl substituents on the electron polarizability of compounds (Table S1 in ESI). The increase in isotropic polarizability could be observed for compounds with alkyl substituents. The influence of the alkyl chains did not affect the polarizability anisotropy which was consistent among all of the compounds. Importantly, the birefringence values also followed this pattern, showing that the substitution of the sexiphenyl core did not strongly affect the magnitude of Δn. 

**Figure 4:**
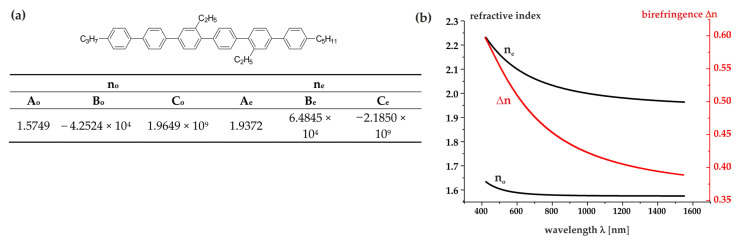
(**a**) Fitting parameters for the extended Cauchy model at 25 °C using the experimental data measured at 443, 636, and 1550 nm wavelengths for **Sexi_Ph4**; (**b**) refractive indices n_e_ and n_o_ and the birefringence dispersion determined for **Sexi_Ph4**.

Figure 4a presents the dispersion of the refractive indices n_e_, n_o_, and birefringence for the tested **Sexi_Ph4** structure. With the values of the refractive indices n_e_ and n_o_ for any three wavelengths, using the methods of mathematical model fitting to the experimental results, we determined the exact values of the three coefficients of the Cauchy equation *A_e,o_*, *B_e,o_*, and *C_e,o_* (Table 3) from Equation (2) as follows:

(2) $$n_{e,o}=A_{e,o}+\frac{B_{e,o}}{\lambda^{2}}+\frac{C_{e,o}}{\lambda^{4}}$$

Having Cauchy coefficients, we further calculated the refractive index and birefringence dispersion in a large range of the electromagnetic spectrum—see Figure 4b.

The material studied belonged to the group of liquid crystals with high birefringence. Its extrapolated birefringence value was 0.4983 for lambda = 636 nm. The linear arrangement of six benzene rings connected by single bonds and extended π-conjugation resulted in high electron polarizability along the long molecular axis, increasing polarizability anisotropy and birefringence. Even in the infrared range wavelengths, these compounds still showed high values of optical anisotropy Δn. Undoubtedly, this should be considered a great advantage of these materials, which, combined with the high chemical stability of such systems, creates many research and application opportunities for them. 

### 2.4. Spectral Properties

**Figure 5:**
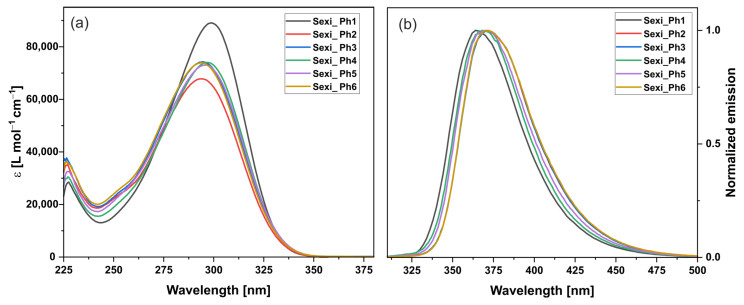
(**a**) Absorption (left) and (**b**) emission (right) spectra of 6P compounds.

The spectral properties of the investigated 6P compounds are displayed in Table 4. The absorption spectra measured in DCM at room temperature are shown in Figure 5a. All the compounds had a strong absorption band in the region of 290–300 nm, with molar absorption coefficients ranging from approximately 73,000 for **Sexi_Ph4** to around 89,000 for **Sexi_Ph6**, and a weaker band was found around 230 nm. Normalized emission spectra are presented in Figure 5b. Compound **Sexi_Ph1** was the only one with blue-shifted emissions—for the rest, there was only minimal difference. All the compounds had high values of quantum yield. 

Computational studies were performed to correlate spectral measurements with the structure of compounds. The optimized ground-state geometries in the gas phase of compounds **Sexi_Ph1**, **Sexi_Ph2**, and **Sexi_Ph4**, together with the non-substituted p-sexiphenyl core [36], are presented in ESI. The dihedral angles of the non-substituted p-sexiphenyl were all around 37°. The introduction of a substituent caused distortion of a torsion angle adjacent to it. Compound **Sexi_Ph1**, bearing two substituents on two adjacent phenyl rings, had the most distorted torsion angle with a value of 91.72°. In the case of other compounds with two substituted phenyl rings separated by one (**Sexi_Ph4**) or two (**Sexi_Ph2**) phenyl rings, torsion angles on adjacent bonds were all approximately 63°. In all compounds, other bonds were not significantly affected by the presence of substituents, with values of approximately 37°. All torsion angles are displayed in detail in ESI. 

Distorted torsion angles in the ground state did not affect excitation energy, but due to the planarization of the molecule in the excited state, they affected emission energy. The small difference between the emission maximum of **Sexi_Ph1** and the rest of the compounds was caused by the slightly bigger dihedral angle in the excited state in this compound. Lateral alkyl substituents in adjacent aromatic rings resulted in almost 45 dihedral angles compared to an average of 40 degrees in other compounds. More planar excited states of compounds **Sexi_Ph2–6** relaxed with lower energy. 

## 3. Materials and Methods

2-Dicyclohexylphosphino-2′,6′-dimethoxybiphenyl (SPhos), 1-bromo-3-chlorobenzene, 1-bromo-4-iodobenzene, and bis(pinacolato)diboron were purchased from Trimen Chemicals (Łódż, Poland) and used as received. Magnesium for Grignard reactions (turnings) was purchased from Acros-Organics, (Geel, Belgium) and used as received. Toluene, acetone, hydrochloric acid, anhydrous potassium carbonate, and cesium carbonate were purchased from Avantor Performance Materials Poland S.A (Gliwice, Poland) and used as received. Palladium (II) chloride was purchased from Merck KGaA (Darmstadt, Germany) and used as received. THF was distilled from sodium under a nitrogen atmosphere before use. 4-pentylphenyl boronic acid, 4′-propylbiphenyl-4yl boronic acid, and 1-ethyl-3-iodobenzene were synthesized in our lab according to common procedures [37,38]. 

Synthesis progress and the purity of the synthesized compounds were determined using a SHIMADZU GCMS-QP2010S (Shimadzu, Kyoto, Japan) series gas chromatograph equipped with a quadrupole mass analyzer MS(EI), high-performance liquid chromatography HPLC-PDA-MS (APCI-ESI dual source) Shimadzu LCMS 2010 EV (Shimadzu, Kyoto, Japan) equipped with a polychromatic UV–VIS detector (Shimadzu, Kyoto, Japan) and by thin-layer chromatography (silica gel on aluminum). Proton (1H) and carbon (13C) nuclear magnetic resonance (NMR) spectra in CDCl3 were collected using a Bruker model Avance III spectrometer (Bruker, Billerica, MA, USA). 

The phase transition temperatures and enthalpy data were determined by polarizing optical microscopy (POM) with an OLYMPUS BX51 (Olympus, Shinjuku, Tokyo, Japan) equipped with a Linkam hot stage THMS-600 (Linkam Scientific Instruments Ltd., Tadworth, UK) and differential scanning calorimeter DSC 204 F1 Phoenix instrument (Netzsch, Selb, Germany) with the scanning rate of 2 Kmin^−1^ on both the heating and cooling cycles with the isothermal time of 5 min between cycles.

Refractive indices of the multicomponent nematic mixtures were measured by The Metricon Model 2010/M Prism Coupler (Metricon Corporation, Pennington, NJ, USA) equipped with 443 nm, 636 nm, and 1550 nm lasers. Samples of liquid crystals were placed on Kapton^®^ MT polyimide film (DuPont, Wilmington, DE, USA). Ordinary refractive index no and extraordinary refractive index ne were measured separately using different polarization of incident beams. Samples were measured at room temperatures (25 °C). 

Solutions of sexiphenyls for absorption measurements were prepared using spectroscopy-grade DCM (Thermo Fisher Scientific, Waltham, MA, USA). The absorption spectra were acquired using a Shimadzu UV–VIS–NIR spectrometer UV-3600 (Shimadzu, Kyoto, Japan). Photoluminescence spectra were acquired using an Edinburgh Instruments FS5 spectrofluorometer (Livingston, UK). Spectra were collected with solutions at a concentration of 0.7–1.2 × 10–5 M. Quantum yield measurements were performed with a calibrated integrating sphere (SC-30 module for FS5 spectrofluorometer, Livingston, UK) using solutions with an absorbance of 0.1 ± 0.05. 

The computational investigation of the properties of selected compounds was performed. Ground-state optimization was performed with the B3-LYP [39,40] functional and 6–31G** [41] basis set. Frequency calculations were performed on optimized structures to verify that the stationery points corresponded to the ground-state minima. Calculations were performed in Gaussian16 (Revision C.01) [42] on the PLGrid ASK Cyfronet Ares cluster.

## 4. Conclusions

In summary, our study presents a novel molecular design approach for liquid crystals based on the structure of para-sexiphenyl (6P). By introducing alkyl terminals and the lateral substitution of the sexiphenyl core, we obtained six new liquid crystalline materials with temperature-broad nematic phases. The synthesis strategy allowed us to tailor the properties of the materials by controlling mainly the positioning of the alkyl chains. The mesomorphic investigations revealed that all compounds displayed a nematic phase, with one member additionally showing a smectic phase. This study highlights several key insights into molecular design. In particular, nonsymmetric compounds consistently exhibit better mesomorphic properties, i.e., they have wider temperature ranges of the nematic phase and higher isotropization temperatures. At the same time, their emission properties compared to their symmetric counterparts remain unchanged. Considering the positions of lateral substituents in the core, it is the lateral substitution of the second and fifth rings on the sexiphenyl core that results in the most favorable combination of individual properties. While symmetric compounds offer simplicity in synthesis, they tend to exhibit inferior mesomorphic properties. Interestingly, our studies have shown that for both lateral and terminal alkyl substitutions, position is more important than length in affecting material properties. Contrary to conventional expectations, changing the length of the terminal alkyl chains (from three to five carbon atoms) does not yield significant improvements in mesomorphic or optical properties. Moreover, the optical properties of the tested material show high birefringence values, even in the infrared range wavelengths. This characteristic, combined with the strong intermolecular interactions and extended π-electron system, makes these materials highly promising for various optical and electronic applications. The combination of polyaromatic and sexiphenyl materials with liquid crystal behavior represents an exciting area of research with significant potential for practical applications in electronics, photonics, optoelectronics, and beyond. Continued research into these materials is likely to uncover new and innovative uses in a range of fields, from electronics and photonics to biomedicine and beyond. We believe that our findings open up new avenues for research and development in the fields of materials science and offer prospects for real-world applications in various devices. 

## Figures and Tables

**Table 1:** Phase transition temperatures and enthalpies of investigated p-sexiphenyls, temperatures, and enthalpies. Red color means the first heating process; blue color means the first cooling process; temperatures are bolded; enthalpy values are italics.

Compound	Phase Transition Temperatures [°C]/(Enthalpy Change ΔH [kJ mol^−1^])	
	Heating Process	Cooling Process
**Sexi_Ph1**	Cr_1_ **77.9** *(−7.0)* Cr_2_ ** 293.1** *(−55.3)* N **345.8** *(−3.8)* Iso	Iso **347.2** *(2.2)* N **273.5** *(0.9)* SmA **243.7** *(22.3)* Cr_2_ **74.5** *(5.6)* Cr_1_
**Sexi_Ph2**	Cr **155.0** *(−30.6)* N **302.9** *(−2.5)* Iso	Iso **303.0** *(2.3)* N **111.6** *(30.3)* Cr_2_ **−39.2** *(0.6)* Cr_1_
**Sexi_Ph3**	Cr_1_ **49.8** *(−4.3)* Cr_2_ **157.7** *(−36.7)* N **244.2** *(−2.4)* Iso	Iso **244.4** *(2.3)* N **117.4** *(23.2)* Cr_2_ **48.7** *(7.3)* Cr_1_
**Sexi_Ph4**	Cr **147.1** *(−17.3)* N **356.0** *(−2.6)* Iso	Iso **357.0** *(2.5)* N **104.3** *(16.8)* Cr
**Sexi_Ph5**	Cr **148.9** *(−22.8)* N **330.0** *(−2.2)* Iso	Iso **329.8** *(2.1)* N **103.5** *(13.1)* Cr
**Sexi_Ph6**	Cr **149.0** *(−26.0)* N **271.8** *(−2.5)* Iso	Iso **272.2** *(2.4)* N **103.0** *(28.0)* Cr

**Table 2:** Composition of nematic matrix mixture.

Compound	n	wt %	Properties
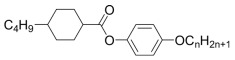	1	32	T_N-Iso_ = 63.6 °C T_Cr-N_ < −20 °C η = 21.2 mPa·s Δn = 0.07; 20 °C (636 nm) Δε = −1.3; 20 °C (1 kHz)
	2	31	
	5	37	

**Table 3:** Refractive indices and birefringence values (extrapolated) for tested **Sexi_Ph4**, **Sexi_Ph5**, and **Sexi_Ph6** materials (T = 25 °C).

Compound	443 nm			636 nm			1550 nm		
	n_o_	n_e_	Δn	n_o_	n_e_	Δn	n_o_	n_e_	Δn
**Sexi_Ph4**	1.6236	2.2105	0.5869	1.5859	2.0842	0.4983	1.5751	1.9638	0.3887
**Sexi_Ph5**	1.6255	2.2072	0.5817	1.5954	2.0807	0.4853	1.5816	1.9577	0.3761
**Sexi_Ph6**	1.6261	2.2011	0.5750	1.5950	2.0741	0.4791	1.5814	1.9518	0.3704

**Table 4:** Spectral properties of 6P compounds.

Compound	$\lambda_{abs}^{max}$[nm]	$\varepsilon_{max}$[L mol^−1^ cm^−1^]	$\lambda_{em}^{max}$[nm]	$\varphi_{fl}$	δν [nm]
**Sexi_Ph1**	299	89,086	364	0.79	65
**Sexi_Ph2**	297	66,972	370	0.68	73
**Sexi_Ph3**	294	74,250	372	0.80	78
**Sexi_Ph4**	298	74,025	368	0.77	70
**Sexi_Ph5**	296	73,039	371	0.79	75
**Sexi_Ph6**	294	73,897	370	0.73	76

## Data Availability

Data are contained within the article and Supplementary Materials.

## Supplementary Materials

The following supporting information can be downloaded at: https://www.mdpi.com/article/10.3390/molecules29050946/s1, Synthesis experimental procedures, Figure S1: 1H NMR of 3-ethyl-4′′-propylterphenyl (3); Figure S2: 13C NMR of 3-ethyl-4′′-propylterphenyl (3); Figure S3: 1H NMR of (3-ethyl-4″-propyl-1,1′:4′,1″-terphenyl-4-yl)boronic acid (5); Figure S4: 13C NMR of (3-ethyl-4″-propyl-1,1′:4′,1″-terphenyl-4-yl)boronic acid (5); Figure S5: 1H NMR of 4-bromo-2′-ethyl-4″-pentyl-1,1′:4′,1″-terphenyl (13_Et); Figure S6: 13C NMR of 4-bromo-2′-ethyl-4″-pentyl-1,1′:4′,1″-terphenyl (13_Et); Figure S7: 1H NMR of (4′-pentyl-3-propylbiphenyl-4-yl)boronic acid (11_Pr); Figure S8: 13C NMR of (4′-pentyl-3-propylbiphenyl-4-yl)boronic acid (11_Pr); Figure S9: 1H NMR of 4,4,5,5-tetramethyl-2-(4″-pentyl-2′-propyl-1,1′:4′,1″-terphenyl-4-yl)-1,3,2-dioxaborolane (14_Pr); Figure S10: 13C NMR of 4,4,5,5-tetramethyl-2-(4″-pentyl-2′-propyl-1,1′:4′,1″-terphenyl-4-yl)-1,3,2-dioxaborolane (14_Pr); Figure S11: 1H NMR of Sexi_Ph2; Figure S12: 13C NMR of Sexi_Ph2; Figure S13: 1H NMR of Sexi_Ph3; Figure S14: 13C NMR of Sexi_Ph3; Figure S15: 1H NMR of Sexi_Ph4; Figure S16: 13C NMR of Sexi_Ph4; Figure S17: 1H NMR of Sexi_Ph5; Figure S18: 13C NMR of Sexi_Ph5; Figure S19: 1H NMR of Sexi_Ph6; Figure S20: 13C NMR of Sexi_Ph6; Figure S21: MS spectrum of 1-chloro-3-propylbenzene (7); Figure S22: MS spectrum of 3-ethyl-4′′-propylterphenyl (3); Figure S23: MS spectrum of 4-iodo-3-ethyl-4′′-propylterphenyl (4); Figure S24: MS spectrum of 4′-pentyl-3-ethyllbiphenyl (9_Et); Figure S25: MS spectrum of 4-iodo-4′-pentyl-3-ethylbiphenyl (10_Et); Figure S26: MS spectrum of 4,4,5,5-tetramethyl-2-(4″-pentyl-2′-ethyl-1,1′:4′,1″-terphenyl-4-yl)-1,3,2-dioxaborolane (14_Et); Figure S27: MS spectrum of 4-bromo-2′-ethyl-4″-pentyl-1,1′:4′,1″-terphenyl (13_Et); Figure S28: MS spectrum of 4′-pentyl-3-propylbiphenyl (9_Pr); Figure S29: MS spectrum of 4-iodo-4′-pentyl-3-propylbiphenyl (10_Pr); Figure S30: MS spectrum of 4,4,5,5-tetramethyl-2-(4″-pentyl-2′-propyl-1,1′:4′,1″-terphenyl-4-yl)-1,3,2-dioxaborolane (14_Pr); Figure S31: MS spectrum of 4-bromo-2′-propyl-4″-pentyl-1,1′:4′,1″-terphenyl (13_Pr); Figure S32: MS spectrum of Sexi_Ph1; Figure S33: MS spectrum of Sexi_Ph2; Figure S34: MS spectrum of Sexi_Ph3; Figure S35: MS spectrum of Sexi_Ph4; Figure S36: MS spectrum of Sexi_Ph5; Figure S37: MS spectrum of Sexi_Ph6; Figure S38: DSC of Sexi_Ph1; Figure S39: DSC of Sexi_Ph2; Figure S40: DSC of Sexi_Ph3; Figure S41: DSC of Sexi_Ph4; Figure S42: DSC of Sexi_Ph5; Figure S43: DSC of Sexi_Ph6; Figure S44: Optimized ground-state geometry of p-sexiphenyl, Sexi_Ph1, Sexi_Ph2, and Sexi_Ph4; Figure S45: Average dihedrals in ground (■) and excited (▲)state for p-sexiphenyl, Sexi_Ph1, Sexi_Ph2, and Sexi_Ph4; Table S1: Calculated values of computed polarizabilites; Table S2: Ground-state Cartesian coordinates of Sexi_Ph1; Table S3: Excited-state Cartesian coordinates of Sexi_Ph1; Table S4: Ground-state Cartesian coordinates of Sexi_Ph2; Table S5: Excited-state Cartesian coordinates of Sexi_Ph2; Table S6: Ground-state Cartesian coordinates of Sexi_Ph4; Table S7: Excited-state Cartesian coordinates of Sexi_Ph4.
