# Supplementary material for: Molecular Design of Sexiphenyl-Based Liquid Crystals: Towards Temperature-Stable, Nematic Phases with Enhanced Optical Properties
Source: Molecules. 2024 Feb 21;29(5):946. doi: 10.3390/molecules29050946 (PMC10934087; doi:10.3390/molecules29050946)
Supplement: Supplementary file 1 [file molecules-29-00946-s001.zip › molecules-2866249-supplementary.pdf]

Supplementary Information

Molecular Design of Sexiphenyl-Based Liquid Crystals: Towards Temperature-Stable, Nematic Phases with Enhanced Optical Properties

Jakub Herman, Piotr Harmata, Natan Rychłowicz and Przemysław Kula*

Institute of Chemistry, Faculty of Advanced Technologies and Chemistry,
Military University of Technology, gen. S. Kaliskiego 2, 00-908 Warsaw, Poland

email: przemyslaw.kula@wat.edu.pl

Contents

1. Synthesis experimental procedures 2
2. Nuclear Magnetic Resonance data 6
3. Mass Spectrum MS(EI) data 27
4. Differential scanning calorimetry (DSC) data 35
5. Computational investigation 39

1. Synthesis experimental procedures

3-ethyl-4''-propyl-1,1':4',1''-terphenyl (3) - flask containing 1-ethyl-3-iodobenzene **(2)** (25g ; 0.108mol), 4’-propylbiphenyl-4yl boronic acid **(1)** (26g ; 0.108mol), potassium acetate (40g ; 0.3mol), acetone (400cm^3) and water (100cm^3) was flushed with nitrogen under reflux for 30min. Then catalyst $PdCl_2$ (3mol%) was added and reaction was refluxed for 10h. Next reaction was cooled to room temperature and poured into 5% HCl solution. Product was extracted with toluene, organic layer was washed with water x3, dried over $MgSO_4$. Solvent was evaporated, product was recrystallized twice from acetone, yield 22g (77%), 1H NMR (500 MHz, $CDCl_3$) δ ppm 1.05 (t, J=7.32 Hz, 3 H) 1.36 (t, J=7.63 Hz, 3 H) 1.76 (m, J=7.48, 7.48, 7.48, 7.48, 7.32 Hz, 2 H) 2.70 (m, 2 H) 2.79 (q, J=7.63 Hz, 2 H) 7.26 (d, J=7.63 Hz, 1 H) 7.33 (d, J=8.24 Hz, 2 H) 7.43 (t, J=7.48 Hz, 1 H) 7.53 (m, 2 H) 7.62 (d, J=8.24 Hz, 2 H) 7.72 (s, 4 H). 13C NMR (500 MHz, $CDCl_3$) d ppm 13.88 ; 15.64 ; 24.53 ; 28.99 ; 37.72 ; 124.39 ; 126.65 ; 126.83 ; 127.23 ; 127.47 ; 128.75 ; 128.91 ; 138.10 ; 139.98 ; 140.04 ; 140.83 ; 141.90 ; 144.73

3-ethyl-4-iodo-4''-propyl-1,1':4',1''-terphenyl (4) - The mixture consisted of 3-ethyl-4''-propyl-1,1':4',1''-terphenyl **(3)** (21.5 g; 0.07 mol), I_2 (7.3 g; 0.029 mol), HIO_3 (2.5 g; 0.014 mmol), glacial acetic acid (500 cm^3), water (50 cm^3) and concentrated sulphuric acid (5 cm3) was stirred for 3 h under reflux. After the completion of reagents, mixture was poured into Na_2SO_3 water solution. The product was isolated and dried. Product **(4)** was recrystallized form acetone, yield 22g (72%).

(3-ethyl-4''-propyl-1,1':4',1''-terphenyl-4-yl)boronic acid (5) - flask containing 3-ethyl-4-iodo-4''-propyl-1,1':4',1''-terphenyl **(4)** (8g ; 19mmol) and anhydrous THF (200cm^3) was flushed with nitrogen and cooled to −78°C in an acetone/dry ice bath. Solution of *tert*-butyllithium dissolved in pentane (23mmol) was added dropwise and temperature was kept below −70°C for 2.5h. Then trimethyl borate (2.4g; 23mmol) was added dropwise and temperature was kept below−70°C. The reaction mixture was allowed to reach room temperature. Then tetrahydrofuran was evaporated and crude product was treated with water and hydrochloric acid. Product was filtered off and dried. m.p=>230°C (decomposition), yield 5.5g (84%), 1H NMR (500 MHz, THF-d8) δ ppm 0.97 (t, J=7.32 Hz, 3 H) 1.26 (t, J=7.48 Hz, 3 H) 1.68 (m, 2 H) 2.63 (t, J=7.63 Hz, 2 H) 2.95 (q, J=7.32 Hz, 2 H) 7.20 (s, 2 H) 7.25 (d, J=7.63 Hz, 2 H) 7.43 (d, J=7.63 Hz, 1 H) 7.48 (s, 1 H) 7.58 (m, 3 H) 7.69 (q, J=8.04 Hz, 4 H). 13C NMR (500 MHz, THF-d8) d ppm 14.12; 17.50; 25.80; 30.10 ; 38.49 ; 123.69 ; 127.24 ; 127.43 ; 127.76 ; 127.97 ; 129.65 ; 134.95 ; 139.08 ; 140.79 ; 140.93 ; 141.98 ; 142.50 ; 150.29

2''',3''-diethyl-4,4'''''-dipropyl-1,1':4',1'':4'',1''':4''',1'''':4'''',1'''''-sexiphenyl Sexi_Ph1 - flask containing a mixture of (3-ethyl-4''-propyl-1,1':4',1''-terphenyl-4-yl)boronic acid **(4)** (2.3 g; 6.7 mmol) and 3-ethyl-4-iodo-4''-propyl-1,1':4',1''-terphenyl **(5)** (1.9 g; 4.4 mmol) in 100 cm^3 of dimethoxyethane, 20 cm^3 of water with 4.4 g (13mmol) of anhydrous caesium carbonate was flushed with nitrogen under reflux for 30min. Then catalyst $PdCl_2$ (3mol%) was added and reaction was refluxed for 10h. Next reaction was cooled to room temperature and poured into 5% HCl solution. Product was filtered off and dried. Product was purified using liquid chromatography (silica gel-dichloromethane) and recrystallization from toluene, yield 1.3g (49%), 1H NMR (500 MHz, $CDCl_3$) δ 0.92 (t, J=7.30 Hz, 6 H) 1.03 (t, J=6.70 Hz, 6 H) 1.63 (m, 4 H) 2.60 (t, 4 H) 3.08 (q, 4 H) 7.08 (d, 2 H) 7.33 (d, J=7.90 Hz, 2 H) 7.46 (s, 2 H) 7.49 (dd, J=7.78, 4 H) 7.58 (d, J=7.93 Hz, 4 H) 7.74 (d, J=8.24 Hz, 4 H) 7.90 (d, J=8.20 Hz, 4 H) 13C NMR (500 MHz, $CDCl_3$) δ 143.28 ; 141.51 ; 139.91 ; 137.6 ; 137.16 ; 132.86 ; 132.58 ; 131.91 ; 130.81 ; 129.91 ; 123.66 ; 122.3 ; 112.65 ; 98.85 ; 37.69 ; 26.27 ; 24.58 ; 15.07 ; 14.21

1-chloro-3-propylbenzene (7) - a solution of 1-bromo-3-chlorobenzene **(6)** (95g , 0.5 mol) in anhydrous THF ($500cm^3$) was added dropwise to Mg chips (12g, 0.5 mol) with vigorous stirring under N_2 atmosphere. The mixture was refluxed for 1 h, then a solution of catalyst Li_2CuCl_4 in anhydrous THF (3mol%) was added. Next, propyl bromide (61g , 0.5 mol) was added dropwise to the mixture which was stirred under reflux for 5h. When the reaction was completed the mixture was poured into $500cm^3$ of H_2O and 100 cm^3 of 10% HCl. The crude product was extracted with hexane. The organic layer was washed with H_2O, separated, dried over $MgSO_4$ and the solvent evaporated. The product was distilled under reduced pressure: b.p. 98-100°C (25 mmHg), yield 24.3 g (32%).

4'-pentyl-3-propylbiphenyl (9_Pr) - flask containing 1-chloro-3-propylbenzene **(7)** (24g ; 0.156mol), 4-pentylbenzene boronic acid **(8)** (33g ; 0.17mol), caesium carbonate (127g ; 0.39mol) and anhydrous tetrahydrofuran ($200cm^3$) was flushed with nitrogen under reflux for 30min. Then catalyst $PdCl_2$ (3mol%) and SPhoS (1.5mol%) were added and reaction was refluxed for 10h. Next reaction was cooled to room temperature and poured into 5% HCl solution. Product was extracted with toluene, organic layer was washed with water, dried over $MgSO_4$. Solvent was evaporated, The product was distilled under reduced pressure: b.p. 150°C (0.1 mmHg), yield 34g (81%).

4'-pentyl-3-ethylbiphenyl (9_Et) – analogous procedure. b.p. 130°C (0.1 mmHg), yield 24g (78%).

4-iodo-4'-pentyl-3-propylbiphenyl (10_Pr) – synthesis using standard iodination procedure described for **(4)**. Product purified using column chromatography (silica gel – hexane). Brown oily liquid

4-iodo-4'-pentyl-3-ethylbiphenyl (10_Et) – synthesis using standard iodination procedure described for **(4)**. Product purified using column chromatography (silica gel – hexane). Brown oily liquid

(4'-pentyl-3-propylbiphenyl-4-yl) boronic acid (11_Pr) – synthesis using standard lithiation-boronation procedure described for **(5)**. White crystals, yield 14g (61%). 1H NMR (500 MHz, THF-d8) δ ppm 0.91 (t, J=6.87 Hz, 3 H) 0.96 (t, J=7.32 Hz, 3 H) 1.35 (s, 1 H) 1.36 (d, J=3.66 Hz, 3 H) 1.66 (m, 4 H) 2.63 (m, 2 H) 2.88 (m, 2 H) 7.16 (s, 2 H) 7.21 (d, J=8.24 Hz, 2 H) 7.35 (d, J=7.63 Hz, 1 H) 7.38 (s, 1 H) 7.52 (d, J=8.24 Hz, 2 H) 7.55 (d, J=7.63 Hz, 1 H). 13C NMR (500 MHz, THF-d8) ppm 14.38 ; 14.51 ; 23.44 ; 26.82 ; 32.18 ; 32.44 ; 36.34 ; 39.20 ; 123.66 ; 127.50 ; 127.98 ; 129.44 ; 134.88 ; 139.72 ; 142.21 ; 142.53 ; 148.49

(4'-pentyl-3-ethylbiphenyl-4-yl)boronic acid (11_Et) – synthesis using standard lithiation-boronation procedure described for **(5)**. White crystals, yield 10g (55%).

4-bromo-4''-pentyl-2'-propyl-1,1':4',1''-terphenyl (13_Pr) - synthesis using standard Suzuki cross-coupling procedure described for **(3)**. Product recrystallized from ethyl alcohol/acetone mixture (5/1 vol/vol), white crystals, yield 13g (90%).

4-bromo-4''-pentyl-2'-ethyl-1,1':4',1''-terphenyl (13_Et) - synthesis using standard Suzuki cross-coupling procedure described for **(3)**. Product recrystallized from ethyl alcohol/acetone mixture (5/1 vol/vol), white crystals, yield 10g (79%). 1H NMR (500 MHz, $CDCl_3$) δ ppm 0.99 (t, J=6.71 Hz, 3 H) 1.21 (t, J=7.48 Hz, 3 H) 1.44 (m, 4 H) 1.74 (m, 2 H) 2.71 (dd, J=7.78, 3.81 Hz, 3 H) 2.74 (s, 1 H) 7.29 (d, J=8.24 Hz, 3 H) 7.34 (d, J=7.93 Hz, 2 H) 7.51 (d, J=7.63 Hz, 1 H) 7.59 (s, 1 H) 7.61 (dd, J=7.93, 5.19 Hz, 4 H). 13C NMR (500 MHz, $CDCl_3$) δ 142.2 ; 141.8 ; 140.7 ; 139.0 ; 138.2 ; 131.2 ; 130.9 ; 130.2 ; 128.8 ; 127.4 ; 126.9 ; 124.3 ; 121.0 ; 35.6 ; 31.6 ; 31.2 ; 26.3 ; 22.6 ; 15.7 ; 14.0

4,4,5,5-tetramethyl-2-(4''-pentyl-2'-propyl-1,1':4',1''-terphenyl-4-yl)-1,3,2-dioxaborolane (14_Pr) - flask containing 4-bromo-4''-pentyl-2'-propyl-1,1':4',1''-terphenyl **(13_Pr)** (7g ; 17mmol), bis(pinacolato)diboron (4.8g ; 18.9mmol), caesium carbonate (19.6g ; 60mmol) and toluene ($200cm^3$) was flushed with nitrogen under reflux for 30min. Then catalyst $Pd(dba)_2$ (2mol%) and XPhoS (2mol%) were added and reaction was refluxed for 10h. Next reaction was cooled to room temperature and poured into 5% HCl solution. Product was extracted with toluene, organic layer was washed with water, dried over $MgSO_4$. Solvent was evaporated, The product was recrystallized from acetone, yield 6g (77%). 1H NMR (500 MHz, $CDCl_3$) δ ppm 0.83 (t, J=7.32 Hz, 3 H) 0.92 (t, J=6.56 Hz, 3 H) 1.36 (d, J=6.71 Hz, 2 H) 1.38 (s, 11 H) 1.53 (m, 2 H) 1.57 (s, 2 H) 1.67 (m, 2 H) 2.64 (dt, J=19.91, 7.90 Hz, 4 H) 7.27 (m, 3 H) 7.37 (d, J=7.93 Hz, 2 H) 7.45 (d, J=7.93 Hz, 1 H) 7.56 (d, J=7.93 Hz, 2 H) 7.87 (d, J=7.93 Hz, 2 H). 13C NMR (500 MHz, $CDCl_3$) δ 13.78 ; 22.31 ; 24.29 ; 24.67 ; 30.92 ; 31.33 ; 35.02 ; 35.35 ; 83.56 ; 123.94 ; 126.70 ; 127.69 ; 128.47 ; 128.54 ; 130.06 ; 134.24 ; 138.14 ; 140.00 ; 140.09 ; 140.24 ; 141.81 ; 144.54

4,4,5,5-tetramethyl-2-(4''-pentyl-2'-ethyl-1,1':4',1''-terphenyl-4-yl)-1,3,2-dioxaborolane (14_Et) - The product was recrystallized from acetone, yield 6g (72%).

2'''',3'-diethyl-4,4'''''-dipentyl-1,1':4',1'':4'',1''':4''',1'''':4'''',1'''''-sexiphenyl Sexi_Ph2 - synthesis using standard Suzuki cross-coupling procedure described for **Sexi_Ph1.** Product was purified using liquid chromatography (silica gel-dichloromethane) and recrystallization from toluene, yield 1.0g (45%), 1H NMR (500 MHz, $CDCl_3$) δ ppm 0.95 (m, 6 H) 1.22 (t, J=7.48 Hz, 6 H) 1.39 (s, 4 H) 1.41 (d, J=3.66 Hz, 4 H) 1.70 (m, J=7.40, 7.40, 7.40, 7.40 Hz, 4 H) 2.69 (m, 4 H) 2.77 (q, J=7.53 Hz, 4 H) 7.30 (d, J=7.93 Hz, 4 H) 7.36 (d, J=7.63 Hz, 2 H) 7.48 (d, J=7.93 Hz, 4 H) 7.51 (dd, J=7.78, 1.68 Hz, 2 H) 7.60 (m, 6 H) 7.75 (d, J=8.24 Hz, 4 H) 13C NMR (500 MHz, $CDCl_3$) δ 142.08 ; 142.00 ; 140.74 ; 140.43 ; 139.95 ; 139.20 ; 138.42 ; 130.45 ; 129.74 ; 128.81 ; 127.35 ; 126.95 ; 126.67 ; 124.28 ; 35.62 ; 31.59 ; 31.18 ; 26.37 ; 22.57 ; 15.77 ; 14.04

2'''',3'-dipropyl-4,4'''''-dipentyl-1,1':4',1'':4'',1''':4''',1'''':4'''',1'''''-sexiphenyl Sexi_Ph3 - synthesis using standard Suzuki cross-coupling procedure described for **Sexi_Ph1.** Product was purified using liquid chromatography (silica gel-dichloromethane) and recrystallization from toluene, yield 1.1g (51%), 1H NMR (500 MHz, $CDCl_3$) δ 0.88 (t, J=7.32 Hz, 6 H) 0.93 (t, J=6.71 Hz, 6 H) 1.38 (m, 8 H) 1.60 (m, 4 H) 1.68 (m, 4 H) 2.68 (s, 8 H) 7.28 (d, J=7.93 Hz, 4 H) 7.33 (d, J=7.93 Hz, 2 H) 7.46 (d, J=7.94 Hz, 4 H) 7.49 (dd, J=7.78, 1.68 Hz, 2 H) 7.54 (s, 2 H) 7.58 (d, J=7.93 Hz, 4 H) 7.74 (d, J=8.24 Hz, 4 H) 13C NMR (500 MHz, $CDCl_3$) δ 142.08 ; 140.86 ; 140.50 ; 140.21 ; 139.12 ; 138.40 ; 130.49 ; 129.79 ; 128.81 ; 127.99 ; 126.96 ; 126.62 ; 124.27 ; 35.62 ; 35.37 ; 31.59 ; 31.18 ; 24.60 ; 22.57 ; 14.11 ; 14.04

2''',3'-diethyl-4-pentyl-4'''''-propyl-1,1':4',1'':4'',1''':4''',1'''':4'''',1'''''-sexiphenyl **Sexi_Ph4** - synthesis using standard Suzuki cross-coupling procedure described for **Sexi_Ph1.** Product was purified using liquid chromatography (silica gel-dichloromethane) and recrystallization from toluene, yield 0.9g (45%), 1H NMR (500 MHz, $CDCl_3$) δ ppm 0.90 (m, 3 H) 0.94 (m, 3 H) 1.00 (t, J=7.32 Hz, 3 H) 1.22 (t, J=7.63 Hz, 3 H) 1.38 (dq, J=3.66, 3.46 Hz, 4 H) 1.60 (m, 2 H) 1.71 (m, 3 H) 2.67 (m, 4 H) 2.71 (m, 2 H) 2.78 (q, J=7.43 Hz, 2 H) 7.29 (dd, J=8.24, 1.83 Hz, 4 H) 7.36 (d, J=7.93 Hz, 1 H) 7.40 (d, J=7.63 Hz, 2 H) 7.42 (s, 3 H) 7.50 (dd, J=7.93, 1.83 Hz, 1 H) 7.55 (s, 2 H) 7.59 (dd, J=8.09, 1.98 Hz, 4 H) 7.62 (d, J=1.53 Hz, 1 H) 7.70 (d, J=8.24 Hz, 2 H) 7.74 (m, 2 H) 13C NMR (500 MHz, $CDCl_3$) δ 142.21 ; 142.11 ; 141.99 ; 140.59 ; 140.54 ; 140.41 ; 140.35 ; 140.20 ; 140.11 ; 140.06 ; 139.96 ; 139.74 ; 138.45 ; 138.15 ; 130.67 ; 130.57 ; 129.05 ; 128.99 ; 128.94 ; 128.86 ; 128.03 ; 127.47 ; 127.42 ; 127.36 ; 127.00 ; 126.90 ; 124.32 ; 124.30 ; 37.78 ; 35.66 ; 35.46 ; 31.63 ; 31.24 ; 26.48 ; 24.64 ; 22.62 ; 15.76 ; 14.17 ; 14.09 ; 13.94

2'''-ethyl-4-pentyl-3',4'''''-dipropyl-1,1':4',1'':4'',1''':4''',1'''':4'''',1'''''-sexiphenyl **Sexi_Ph5** - synthesis using standard Suzuki cross-coupling procedure described for **Sexi_Ph1.** Product was purified using liquid chromatography (silica gel-dichloromethane) and recrystallization from toluene, yield 1.0g (49%), 1H NMR (500 MHz, $CDCl_3$) δ 0.92 (s, 3 H) 0.96 (s, 3 H) 1.03 (s, 3 H) 1.25 (s, 3 H) 1.41 (s, 4 H) 1.63 (s, 2 H) 1.72 (s, 4 H) 2.71 (s, 6 H) 2.80 (s, 2 H) 7.31 (s, 4 H) 7.38 (s, 1 H) 7.42 (s, 1 H) 7.45 (s, 4 H) 7.52 (s, 1 H) 7.58 (s, 2 H) 7.61 (s, 4 H) 7.65 (s, 1 H) 7.72 (s, 2 H) 7.76 (s, 2 H) 13C NMR (500 MHz, $CDCl_3$) δ 142.15 ; 142.04 ; 141.92 ; 140.52 ; 140.49 ; 140.36 ; 140.31 ; 140.15 ; 140.05 ; 140.01 ; 139.9 ; 139.69 ; 138.40 ; 138.09 ; 130.62 ; 130.52 ; 129.01 ; 128.93 ; 128.89 ; 128.80 ; 127.79 ; 127.41 ; 127.36 ; 127.30 ; 126.95 ; 126.85 ; 124.28 ; 124.25 ; 37.73 ; 35.62 ; 35.42 ; 31.59 ; 31.18 ; 26.43 ; 24.57 ; 24.54 ; 22.57 ; 15.71 ; 14.12 ; 14.04 ; 13.89

2''''-ethyl-4,4'''''-dipentyl-3'-propyl-1,1':4',1'':4'',1''':4''',1'''':4'''',1'''''-sexiphenyl **Sexi_Ph6** - synthesis using standard Suzuki cross-coupling procedure described for **Sexi_Ph1.** Product was purified using liquid chromatography (silica gel-dichloromethane) and recrystallization from toluene, yield 1.3g (55%), 1H NMR (500 MHz, $CDCl_3$) δ ppm 0.91 (t, J=7.32 Hz, 3 H) 0.95 (m, 6 H) 1.23 (t, J=7.48 Hz, 3 H) 1.38 (s, 1 H) 1.40 (dd, J=6.71, 3.36 Hz, 7 H) 1.63 (m, 2 H) 1.70 (dd, J=14.80, 7.17 Hz, 4 H) 2.71 (m, 6 H) 2.77 (q, J=7.43 Hz, 2 H) 7.31 (d, J=6.71 Hz, 4 H) 7.36 (d, J=7.93 Hz, 2 H) 7.49 (m, 6 H) 7.57 (d, J=1.83 Hz, 1 H) 7.59 (s, 1 H) 7.61 (dd, J=8.09, 1.98 Hz, 4 H) 7.76 (m, 4 H) 13C NMR (500 MHz, $CDCl_3$) δ 142.08 ; 142.00 ; 140.84 ; 140.73 ; 140.49 ; 140.43 ; 140.18 ; 139.95 ; 139.18 ; 139.10 ; 138.41 ; 138.38 ; 130.49 ; 130.45 ; 129.75 ; 128.82 ; 127.98 ; 127.35 ; 126.96 ; 126.66 ; 126.63 ; 124.28 ; 35.62 ; 35.37 ; 31.59 ; 31.19 ; 26.37 ; 24.61 ; 22.57 ; 15.79 ; 14.12 ; 14.05

2. Nuclear Magnetic Resonance data

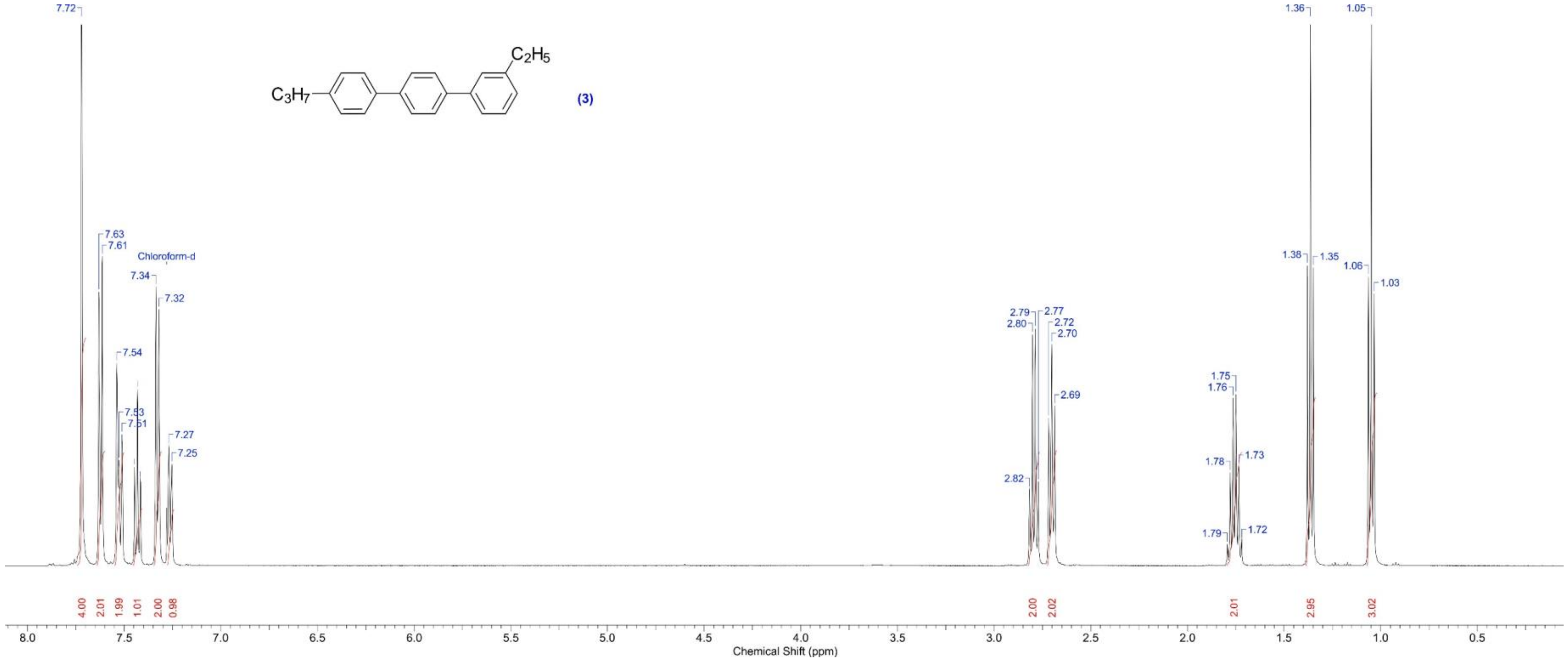

Figure S1. 1H NMR of 3-ethyl-4’’-propylterphenyl (**3**).

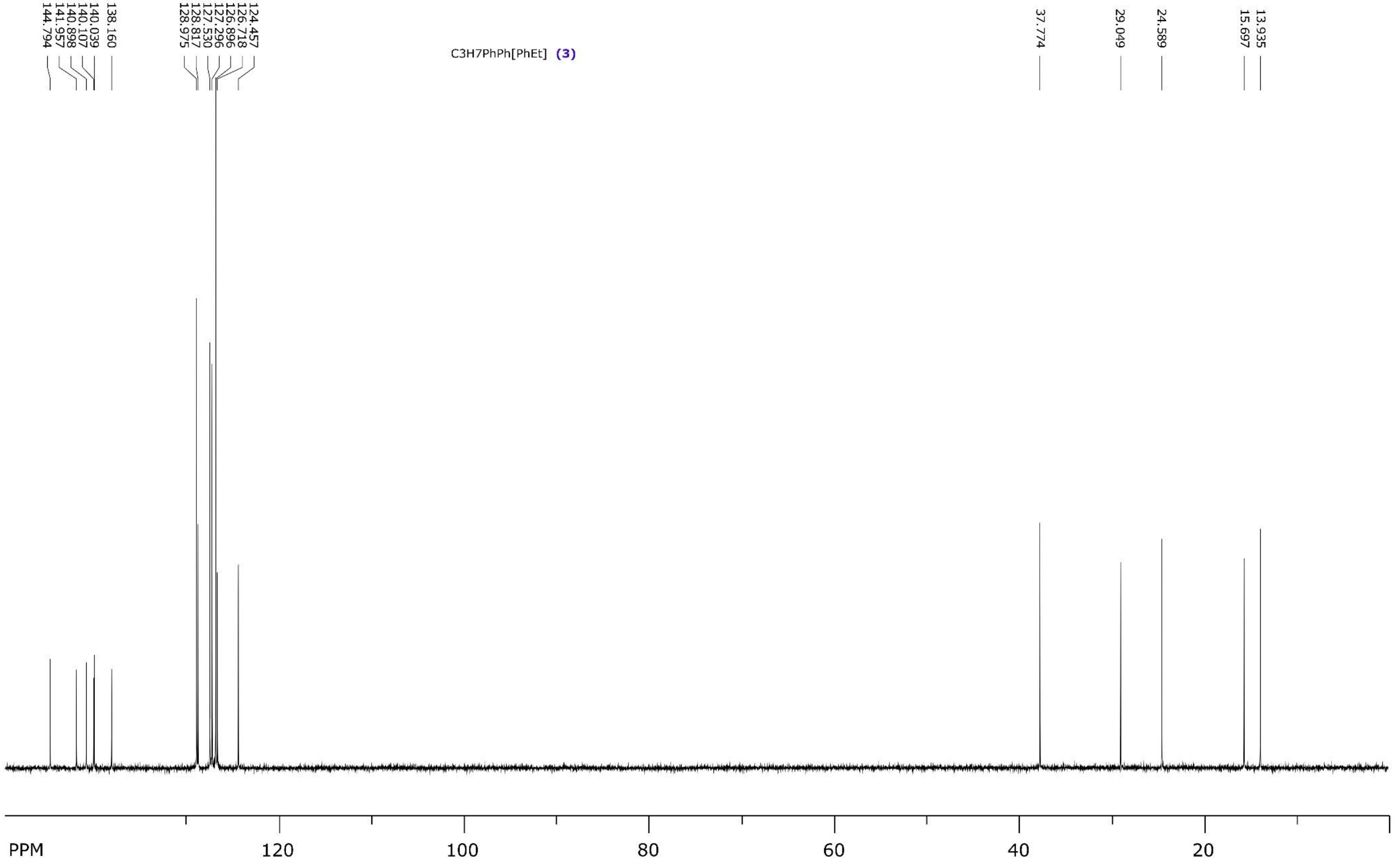

Figure S2. 13C NMR of 3-ethyl-4’’-propylterphenyl (**3**).

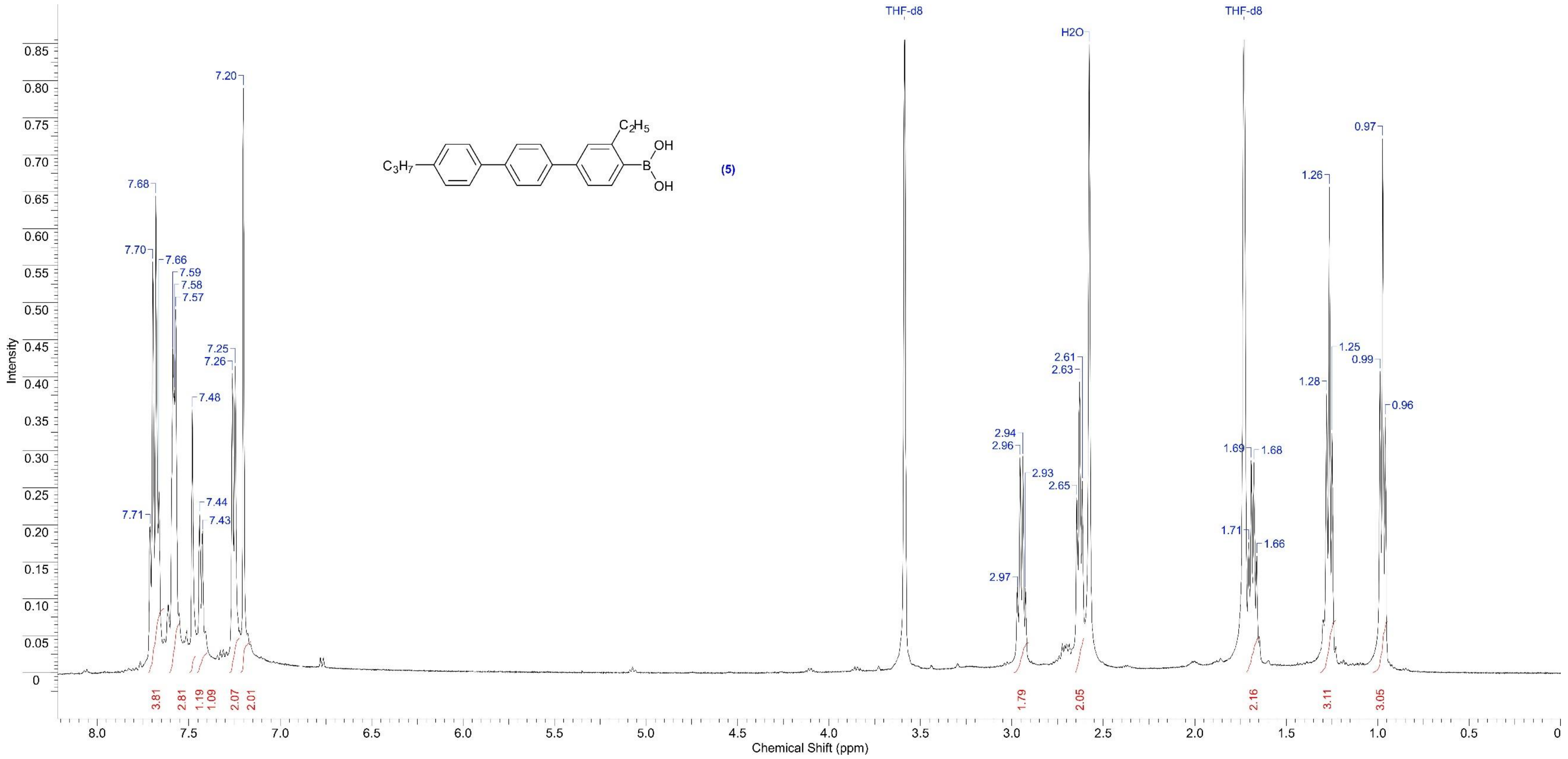

Figure S3. 1H NMR of (3-ethyl-4''-propyl-1,1':4',1''-terphenyl-4-yl)boronic acid (**5**).

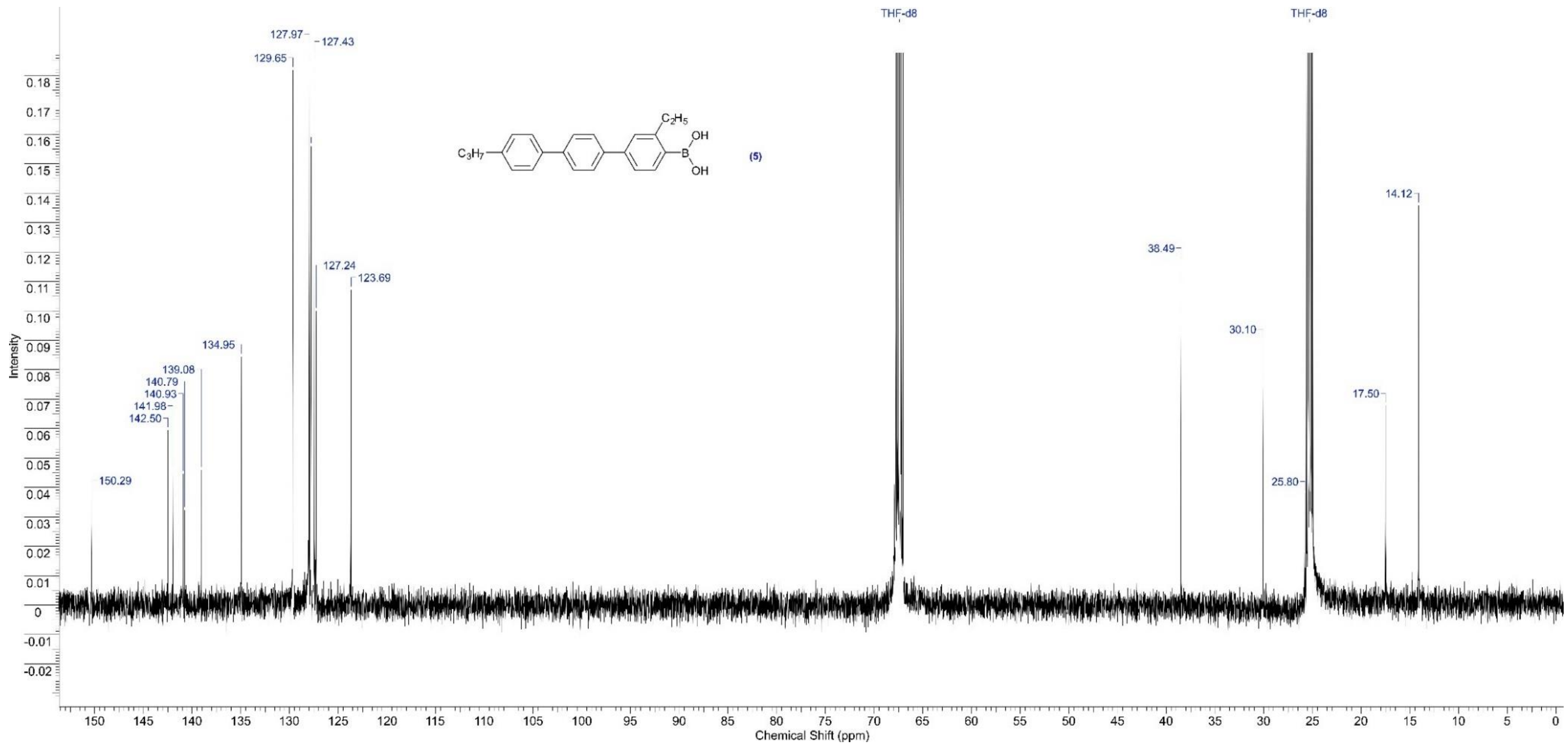

Figure S4. 13C NMR of (3-ethyl-4''-propyl-1,1':4',1''-terphenyl-4-yl)boronic acid (**5**).

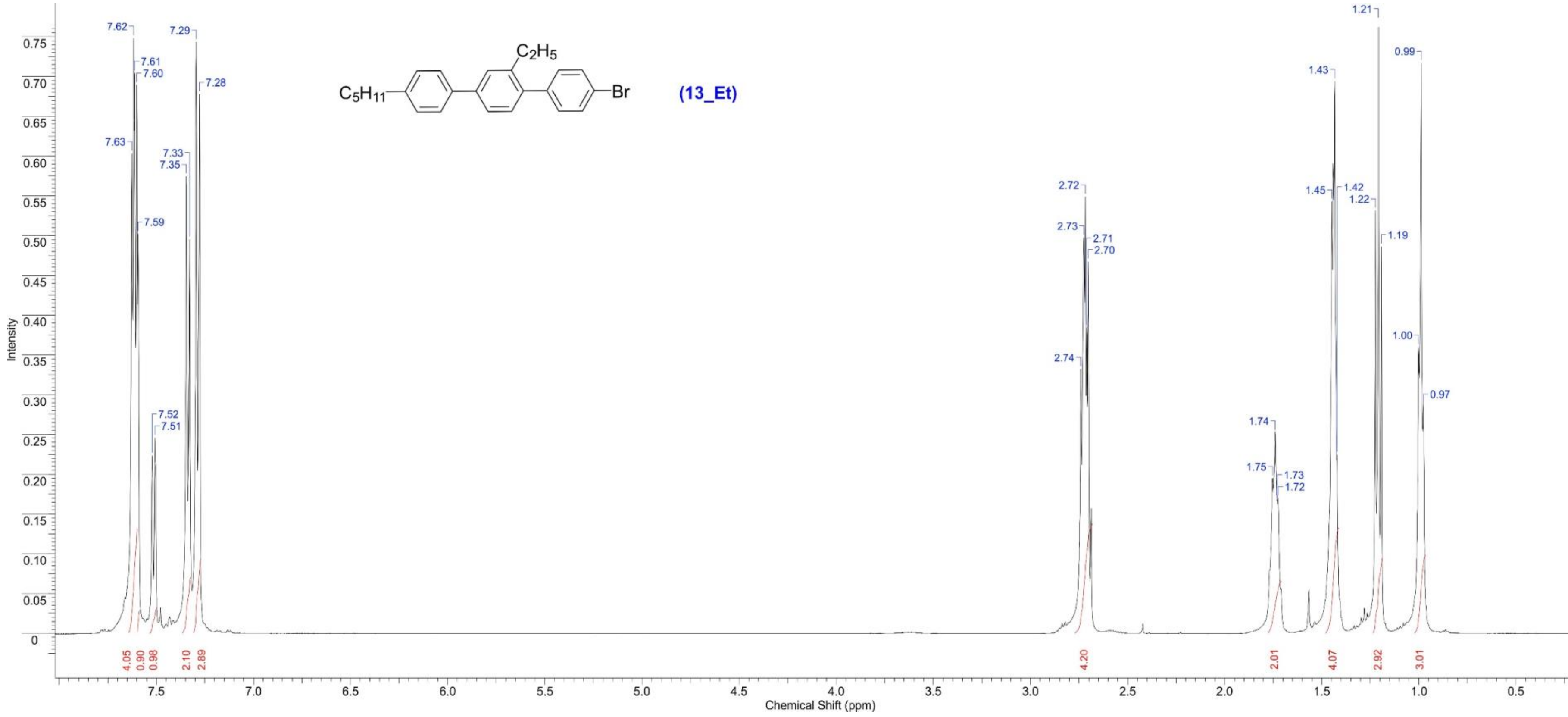

Figure S5. 1H NMR of 4-bromo-2'-ethyl-4''-pentyl-1,1':4',1''-terphenyl (**13_Et**).

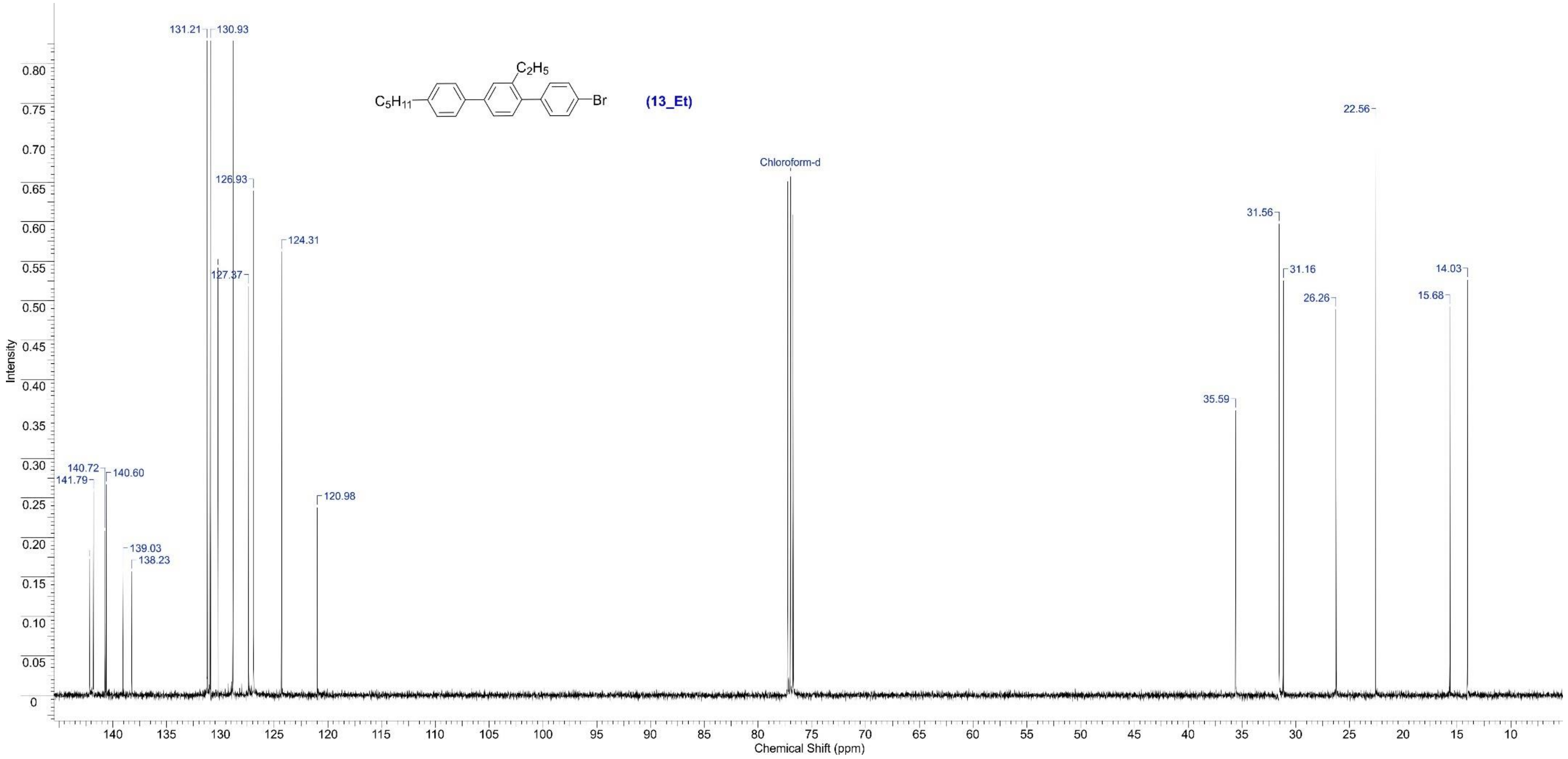

Figure S6. 13C NMR of 4-bromo-2'-ethyl-4''-pentyl-1,1':4',1''-terphenyl (**13_Et**).

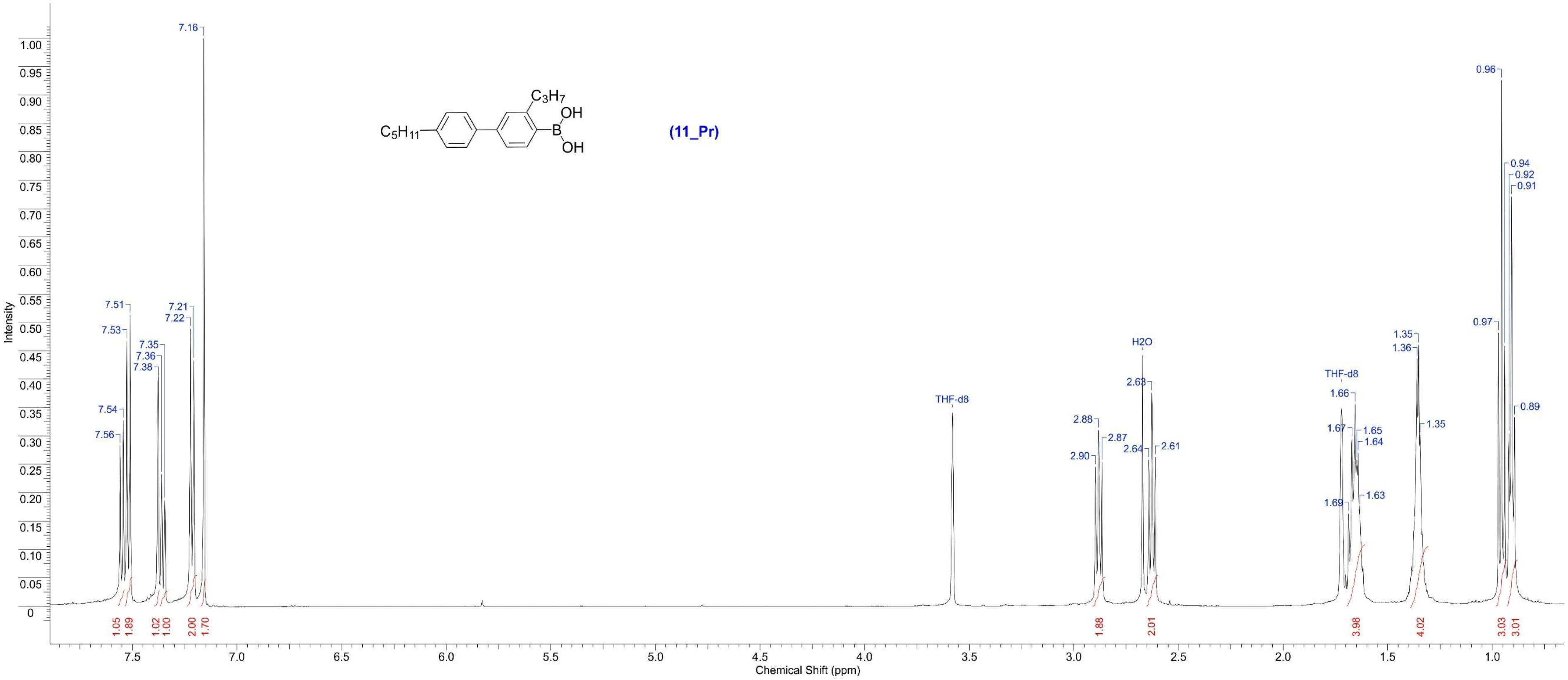

Figure S7. 1H NMR of (4'-pentyl-3-propylbiphenyl-4-yl)boronic acid (**11_Pr**).

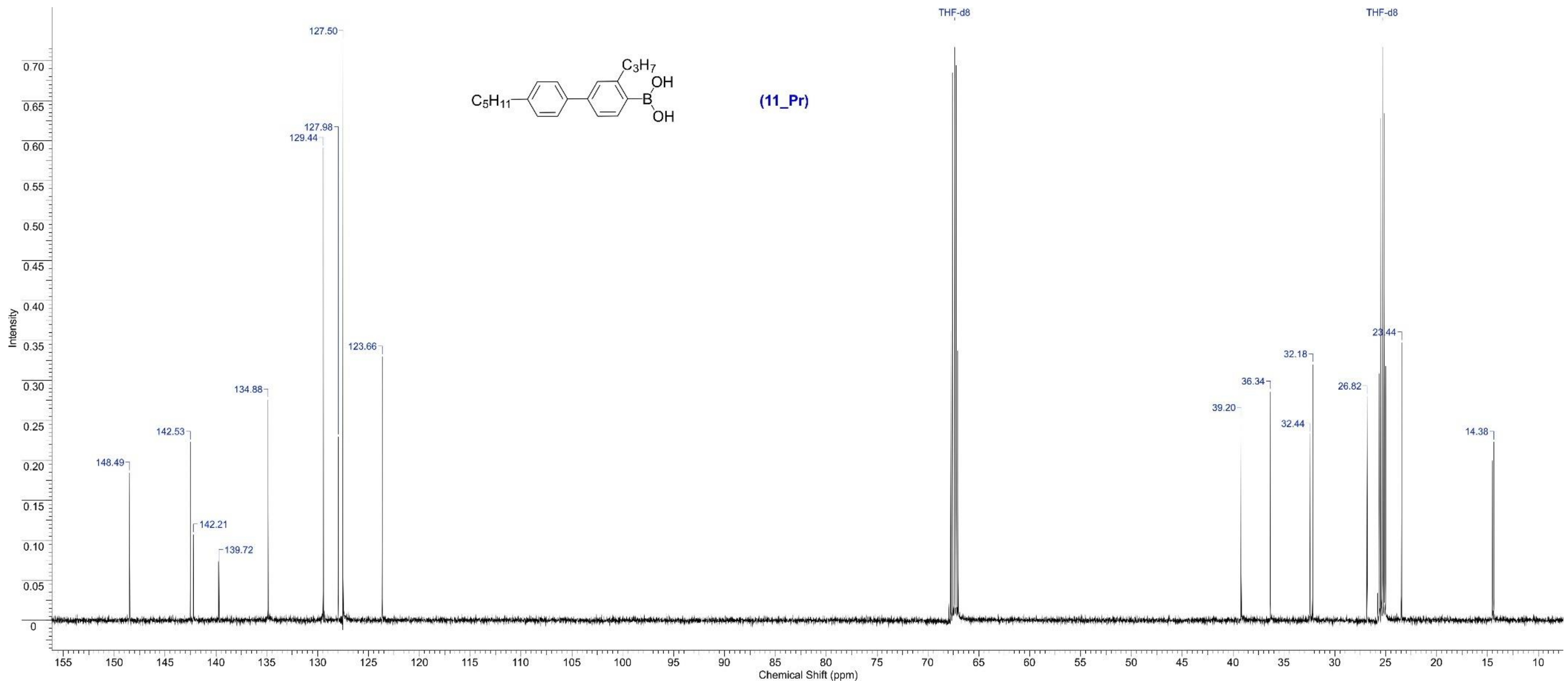

Figure S8. 13C NMR of (4'-pentyl-3-propylbiphenyl-4-yl)boronic acid (**11_Pr**).

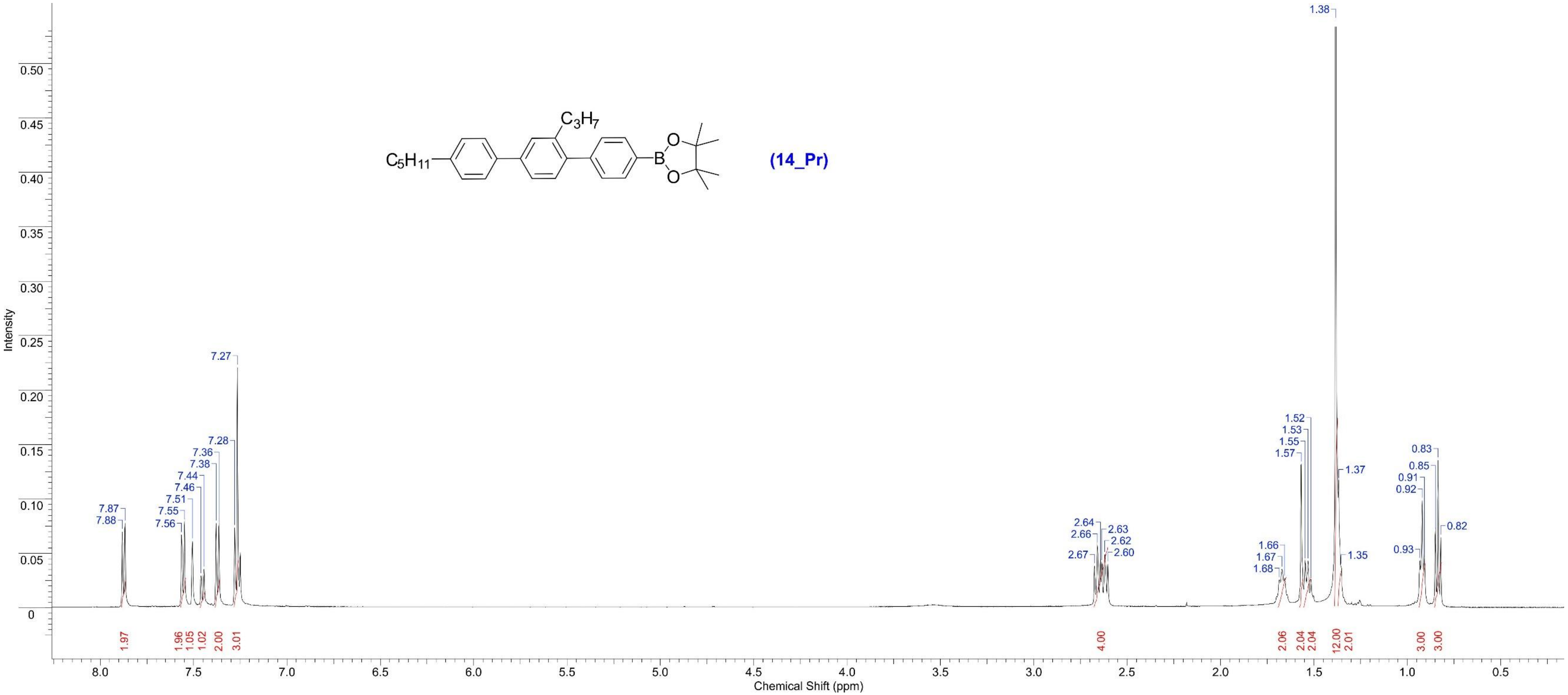

Figure S9. 1H NMR of 4,4,5,5-tetramethyl-2-(4''-pentyl-2'-propyl-1,1':4',1''-terphenyl-4-yl)-1,3,2-dioxaborolane (**14_Pr**).

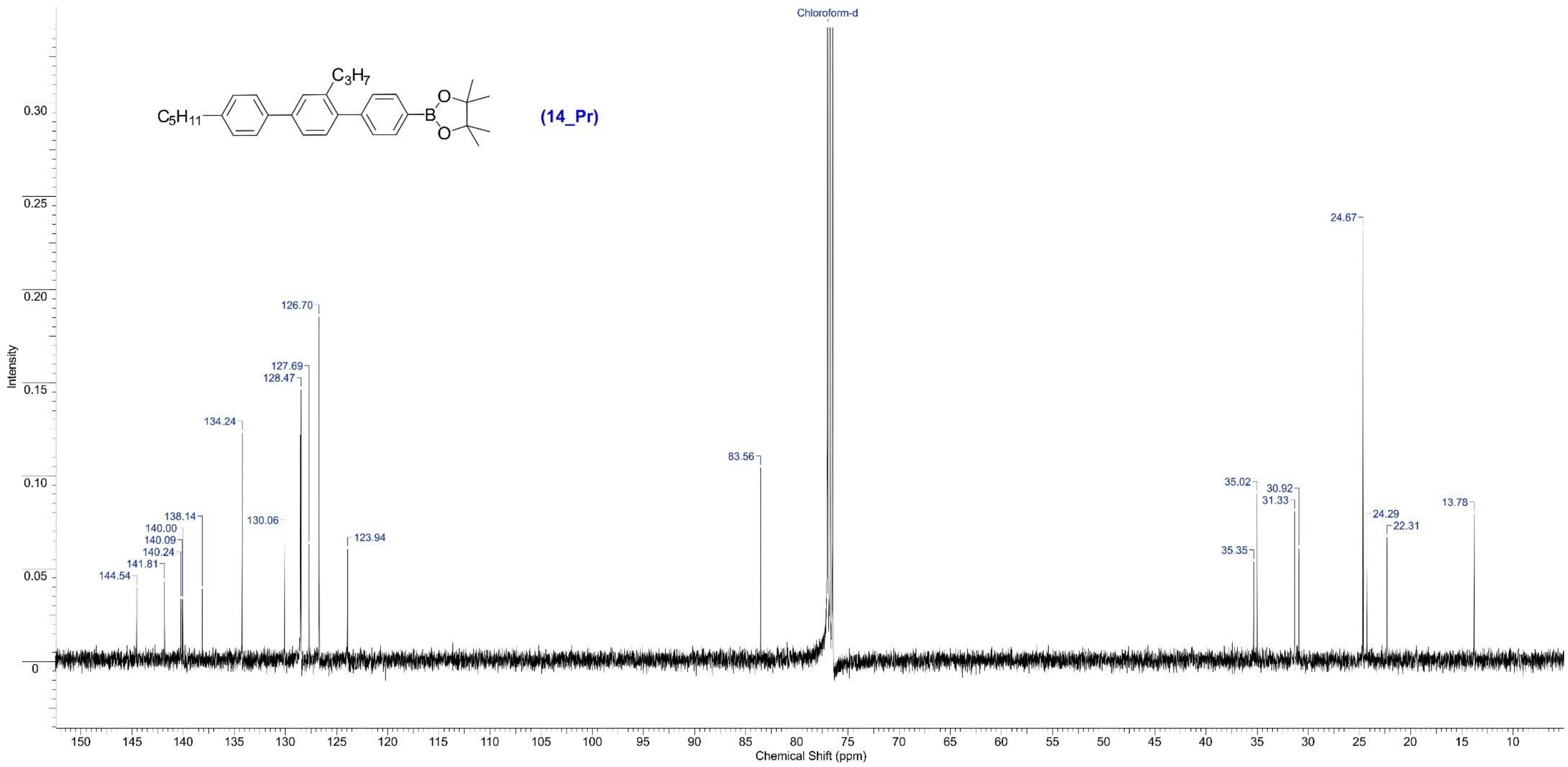

Figure S10. 13C NMR of 4,4,5,5-tetramethyl-2-(4''-pentyl-2'-propyl-1,1':4',1''-terphenyl-4-yl)-1,3,2-dioxaborolane (**14_Pr**).

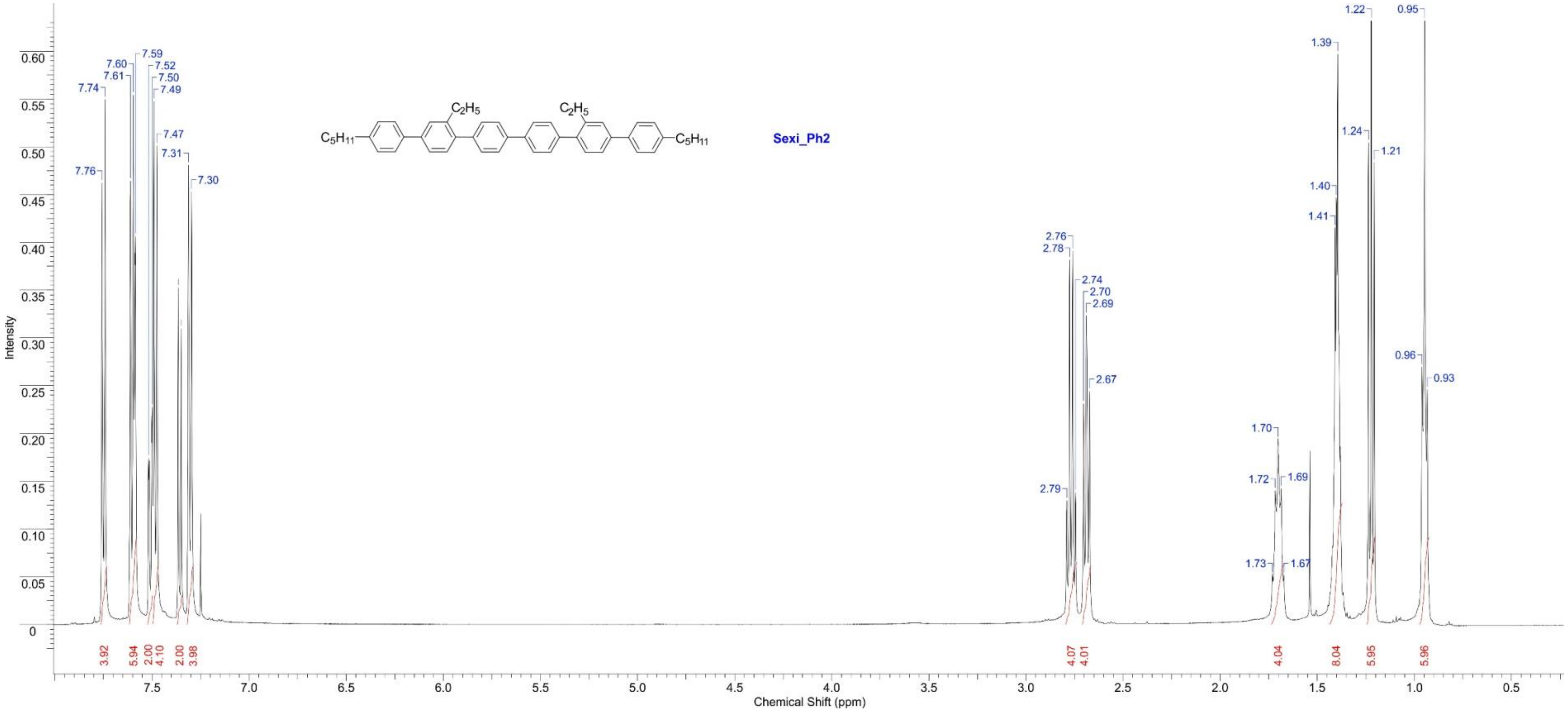

Figure S11. 1H NMR of **Sexi_Ph2**.

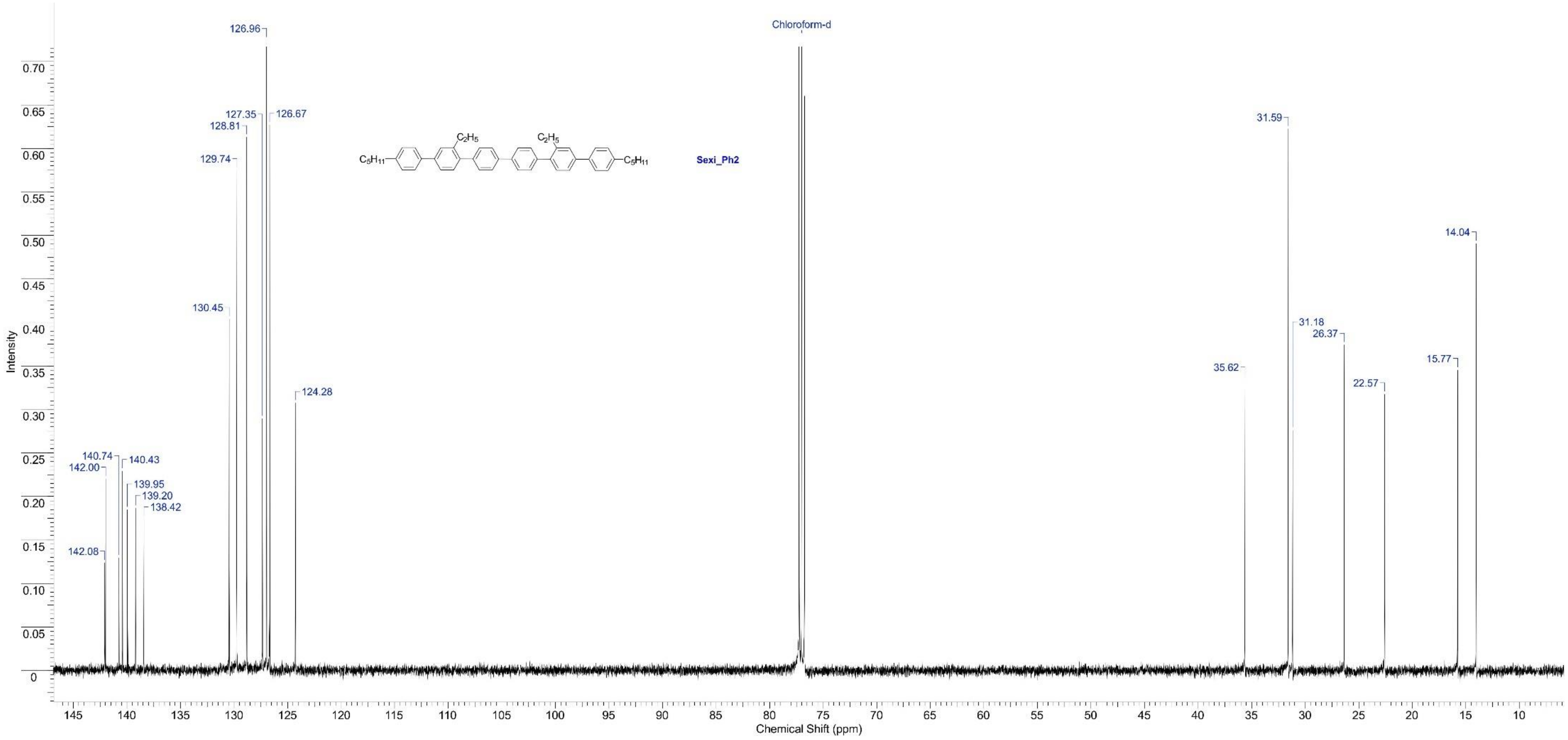

Figure S12. 13C NMR of **Sexi_Ph2**.

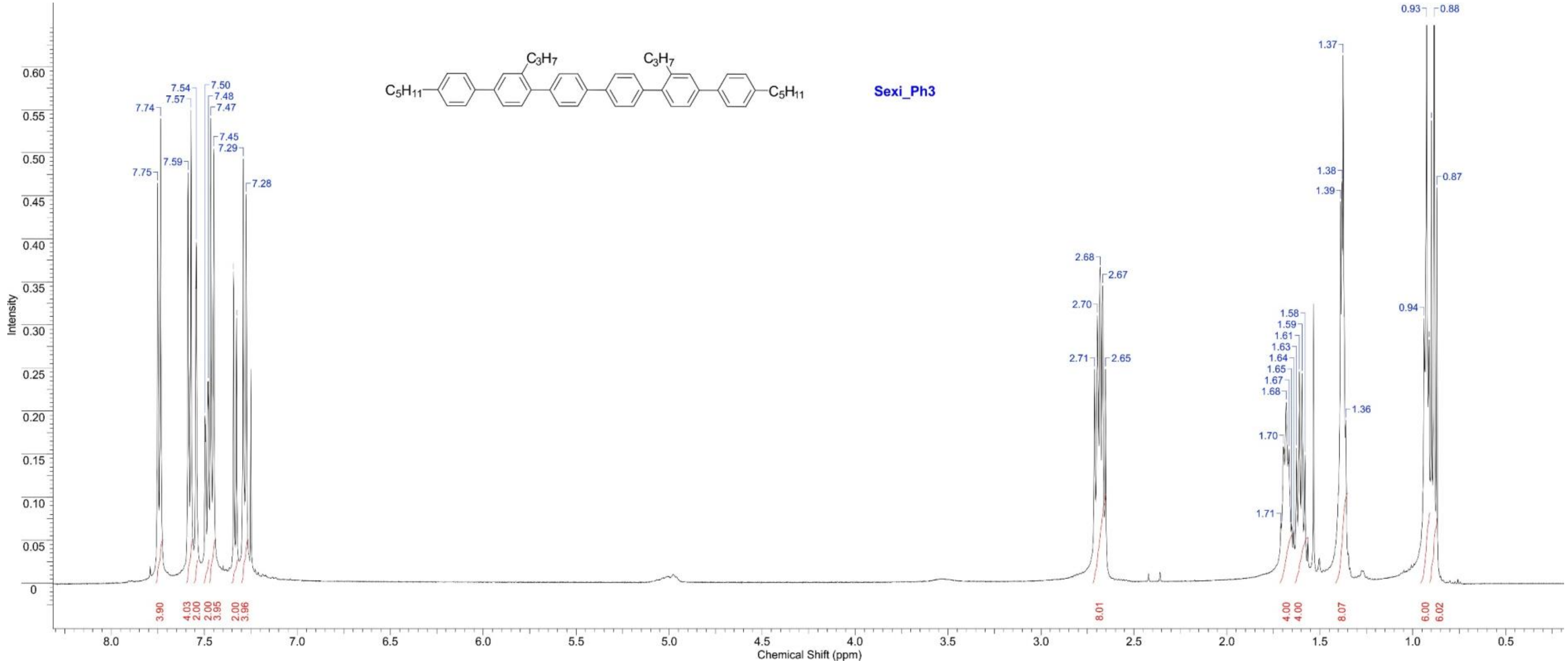

Figure S13. 1H NMR of **Sexi_Ph3**.

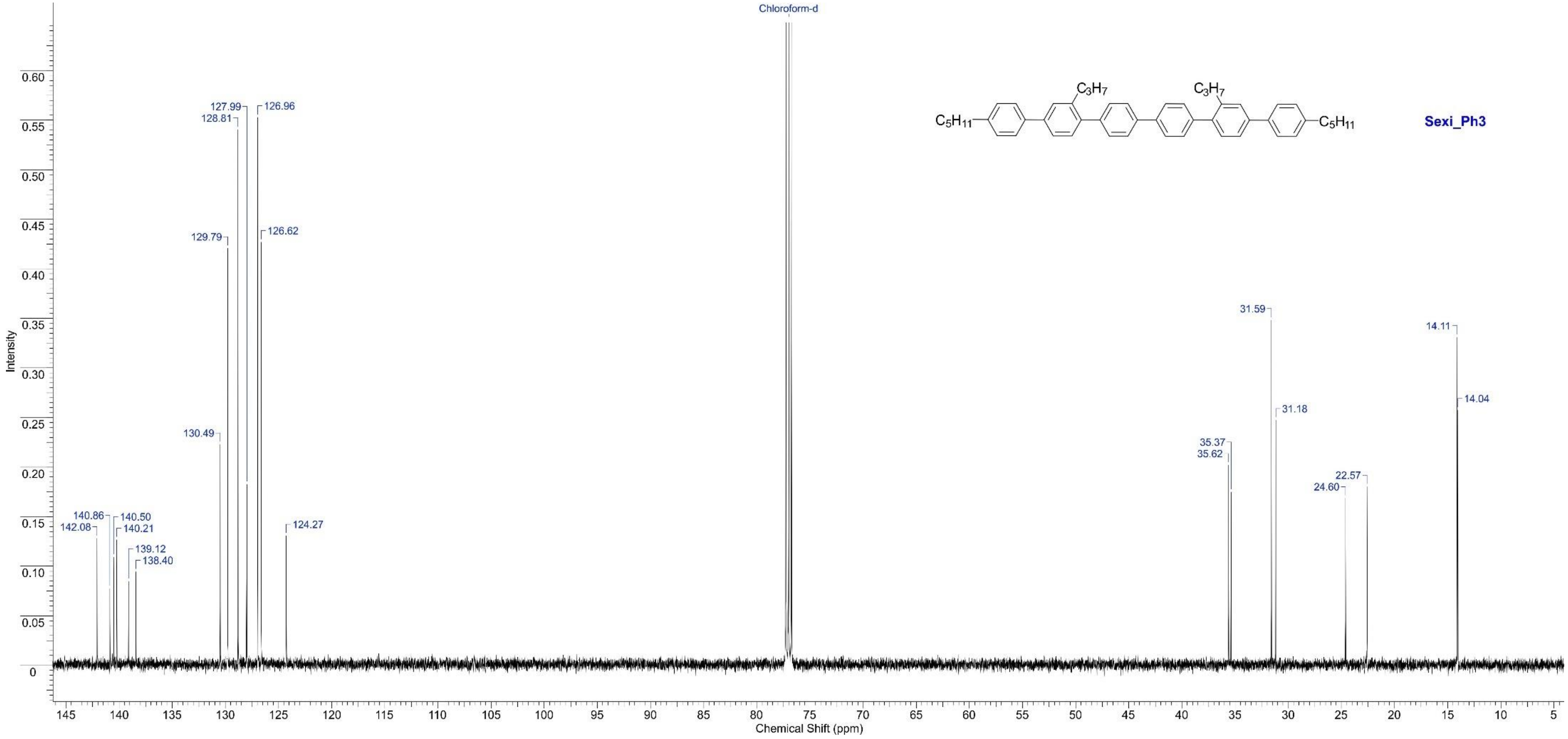

Figure S14. 13C NMR of **Sexi_Ph3**.

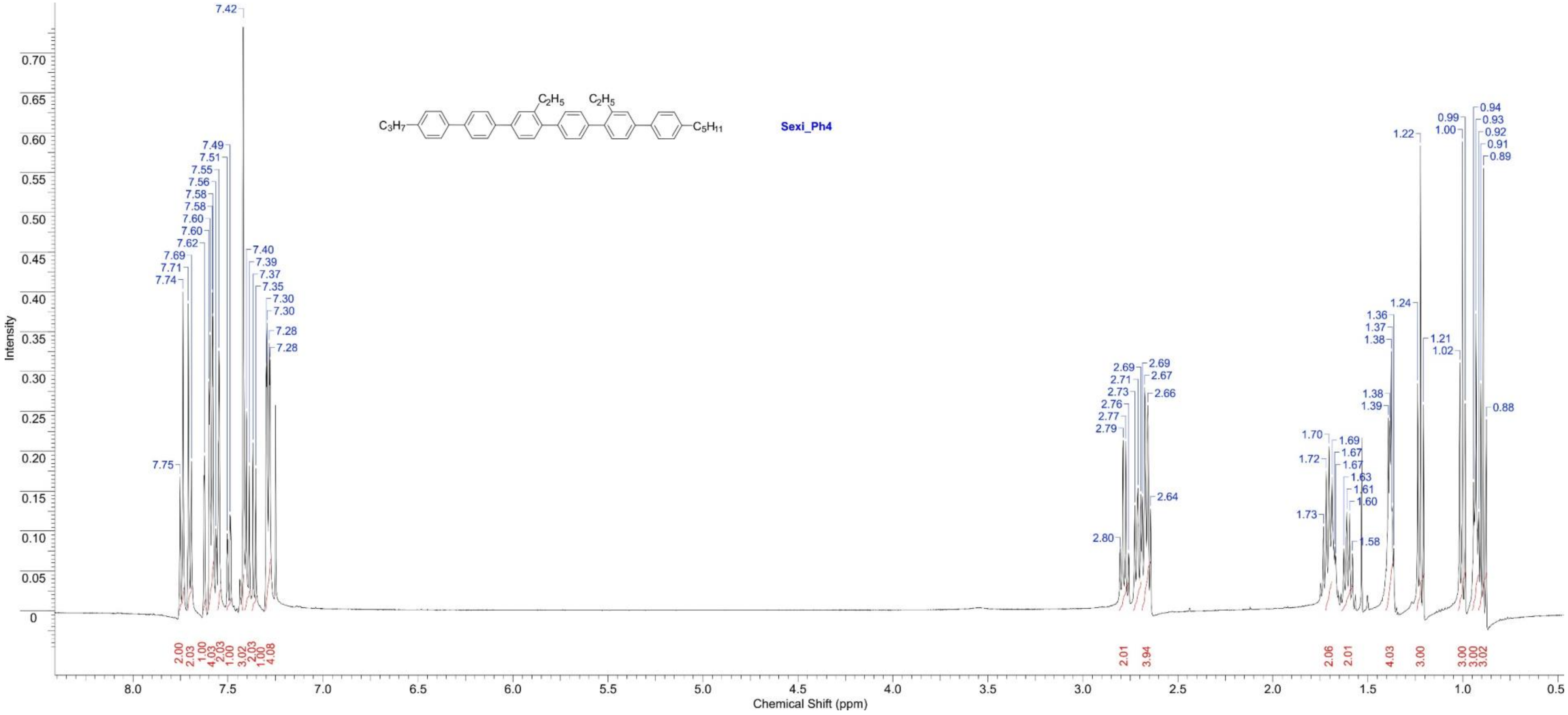

Figure S15. 1H NMR of **Sexi_Ph4**.

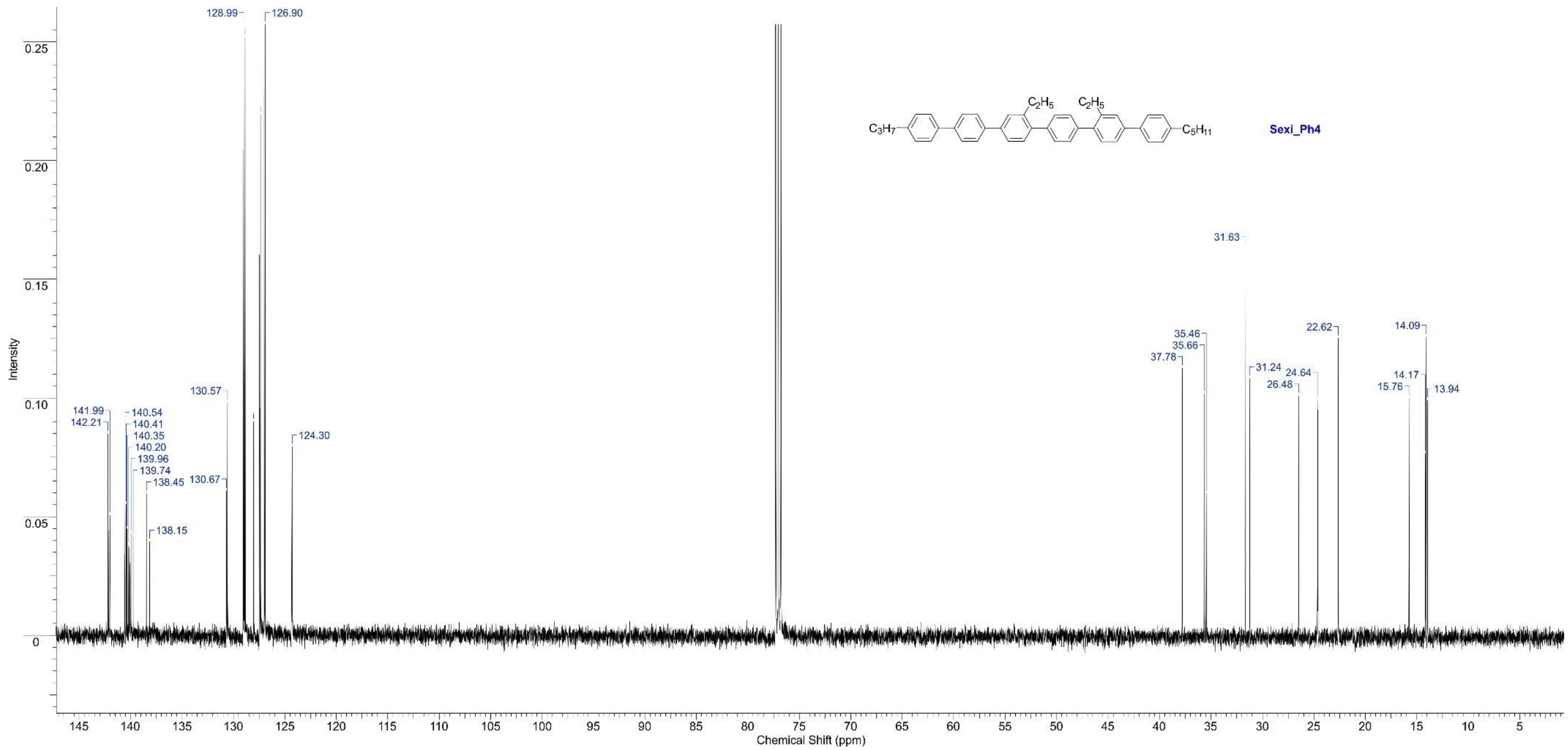

Figure S16. 13C NMR of **Sexi_Ph4**.

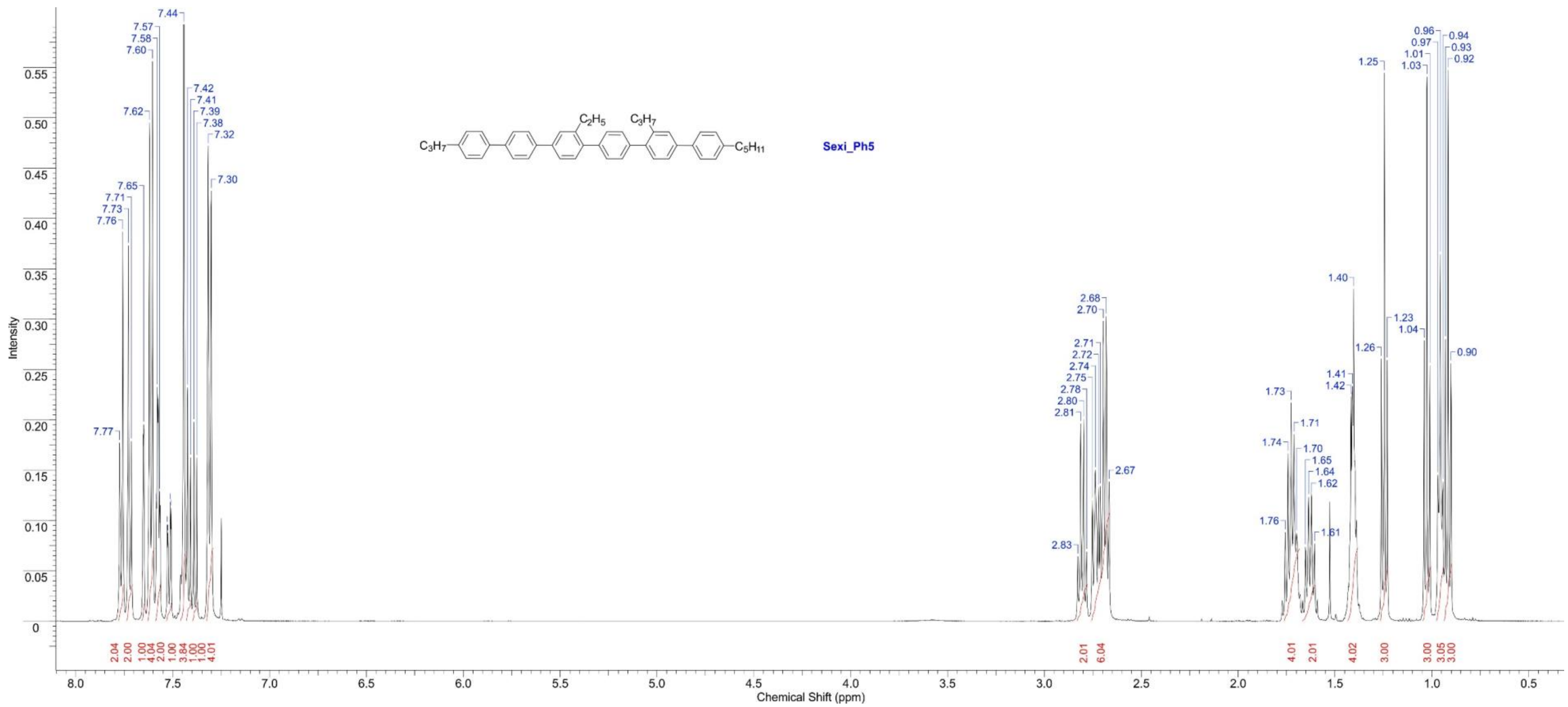

Figure S17. 1H NMR of **Sexi_Ph5**.

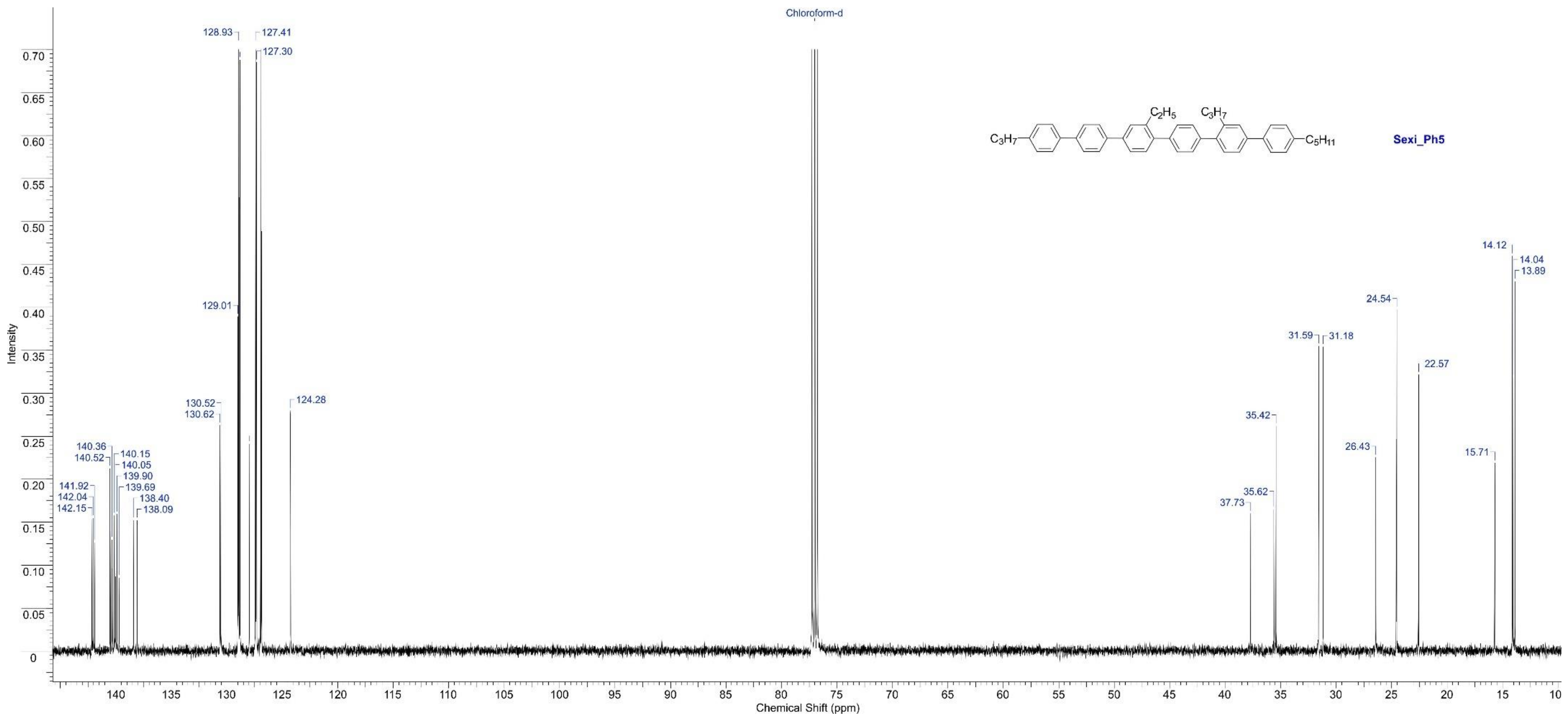

Figure S18. 13C NMR of **Sexi_Ph5**.

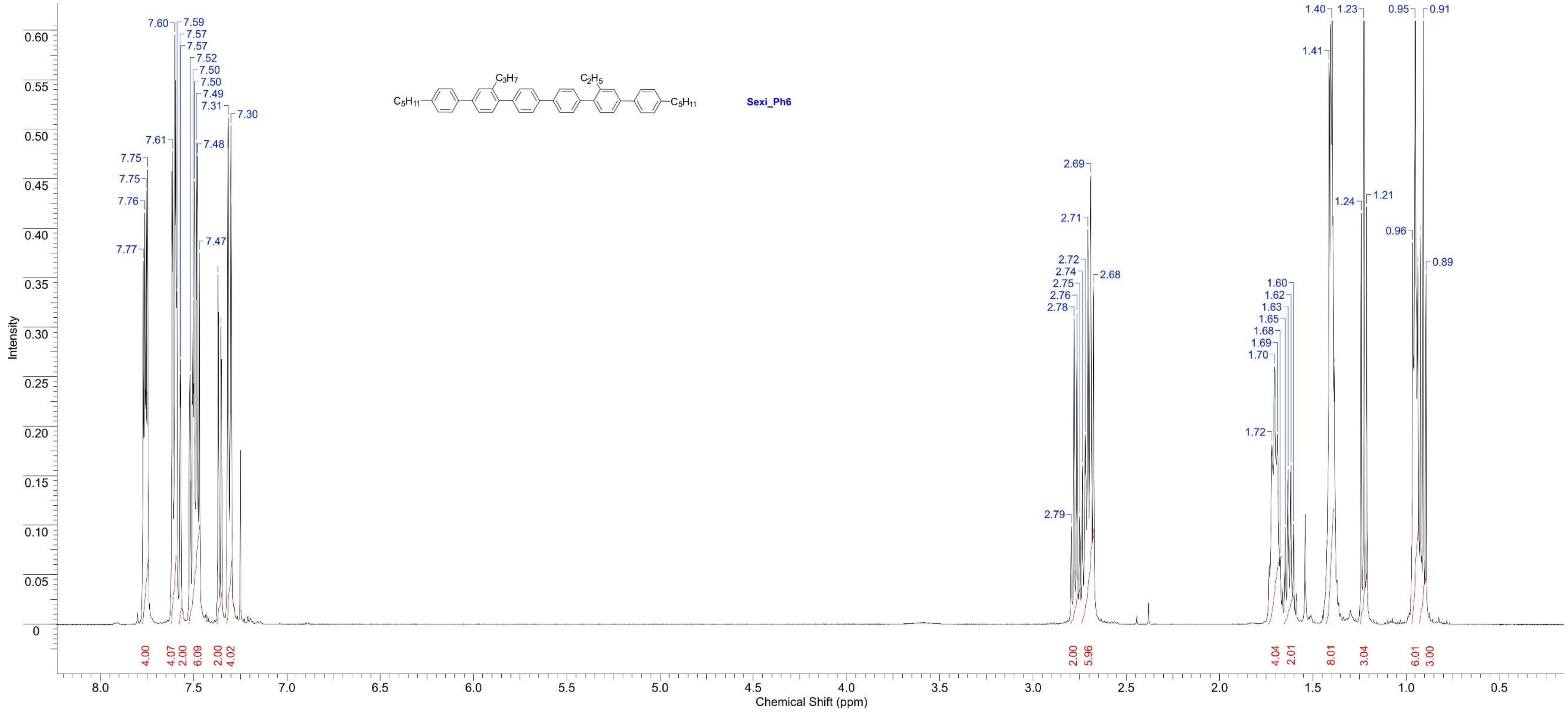

Figure S19. 1H NMR of **Sexi_Ph6**.

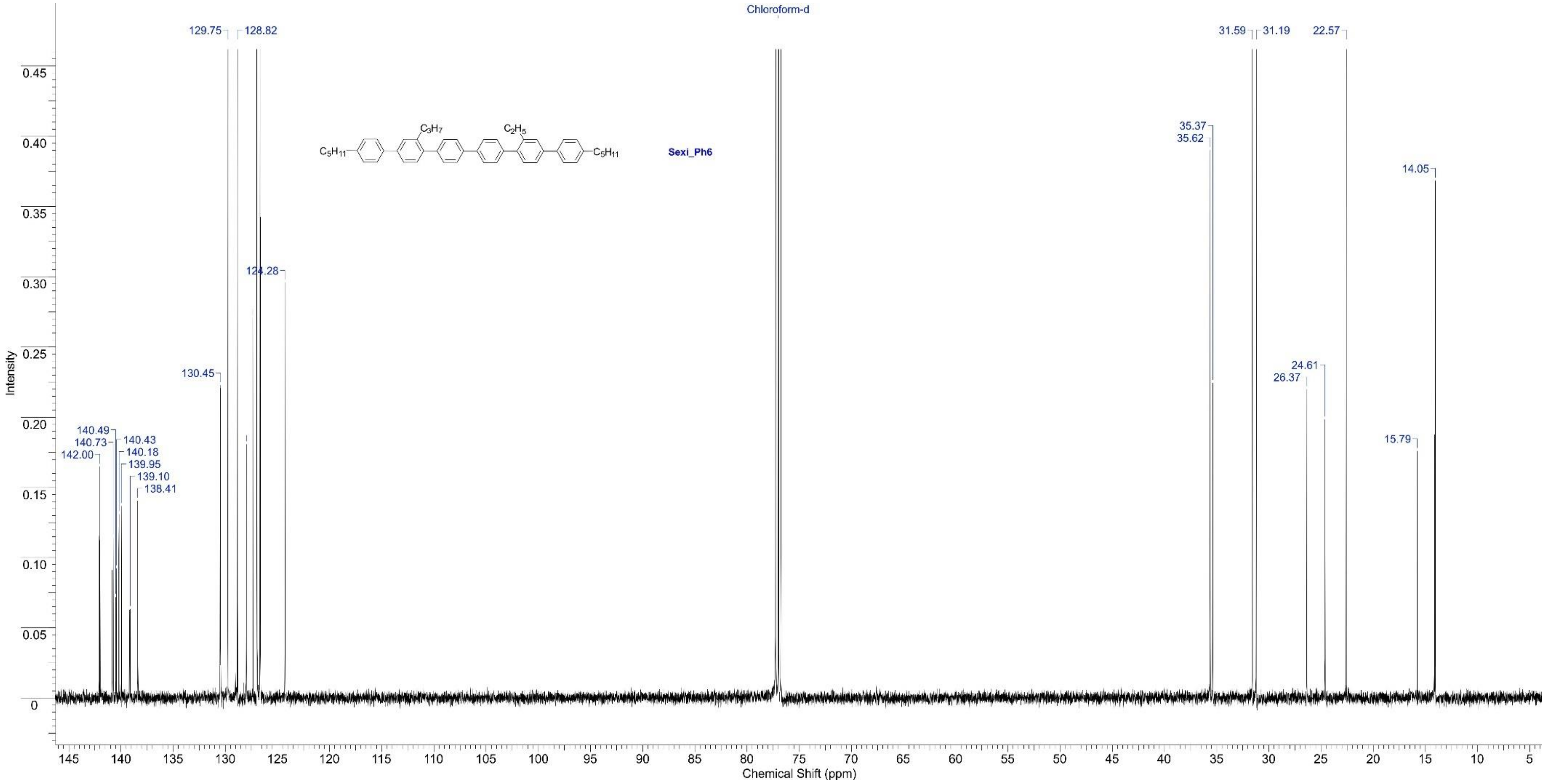

Figure S20. 13C NMR of **Sexi_Ph6**.

3. Mass Spectrum MS(EI) data

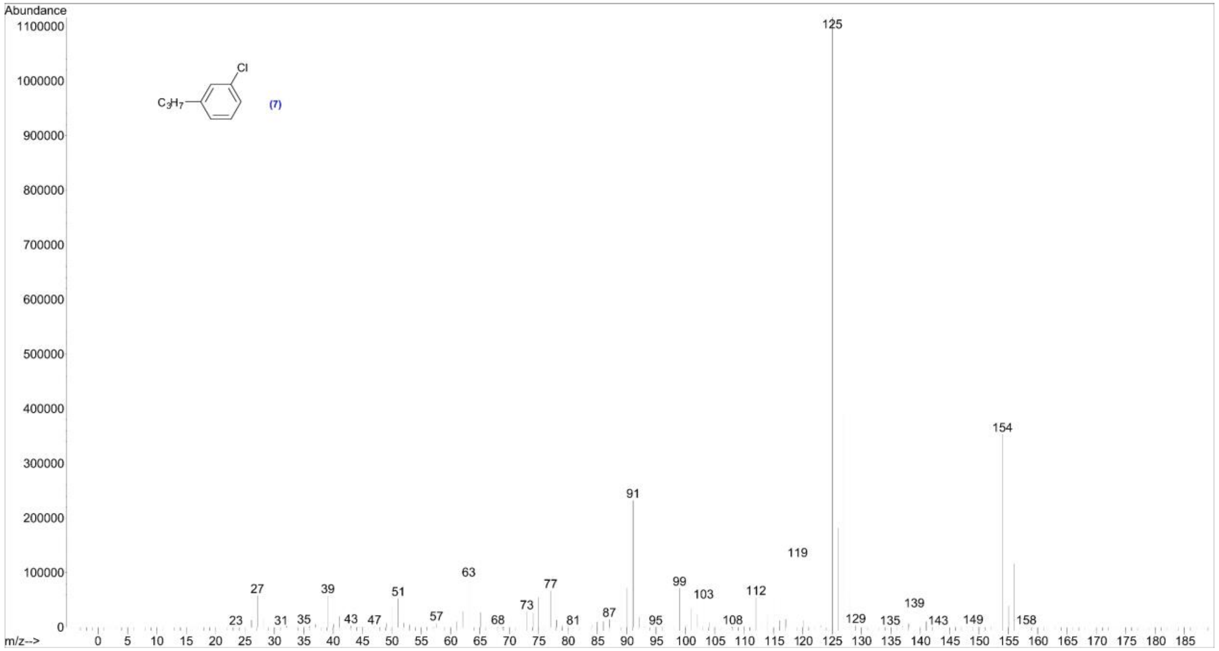

Figure S21. MS spectrum of 1-chloro-3-propylbenzene (**7**).

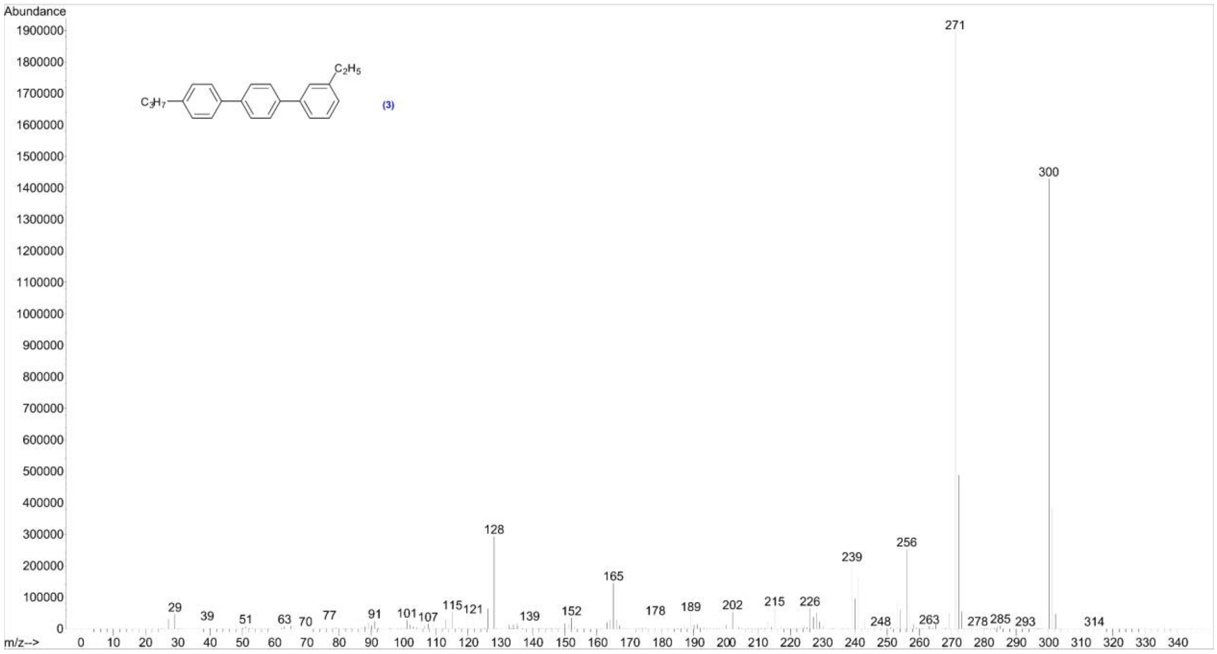

Figure S22. MS spectrum of 3-ethyl-4''-propylterphenyl (**3**).

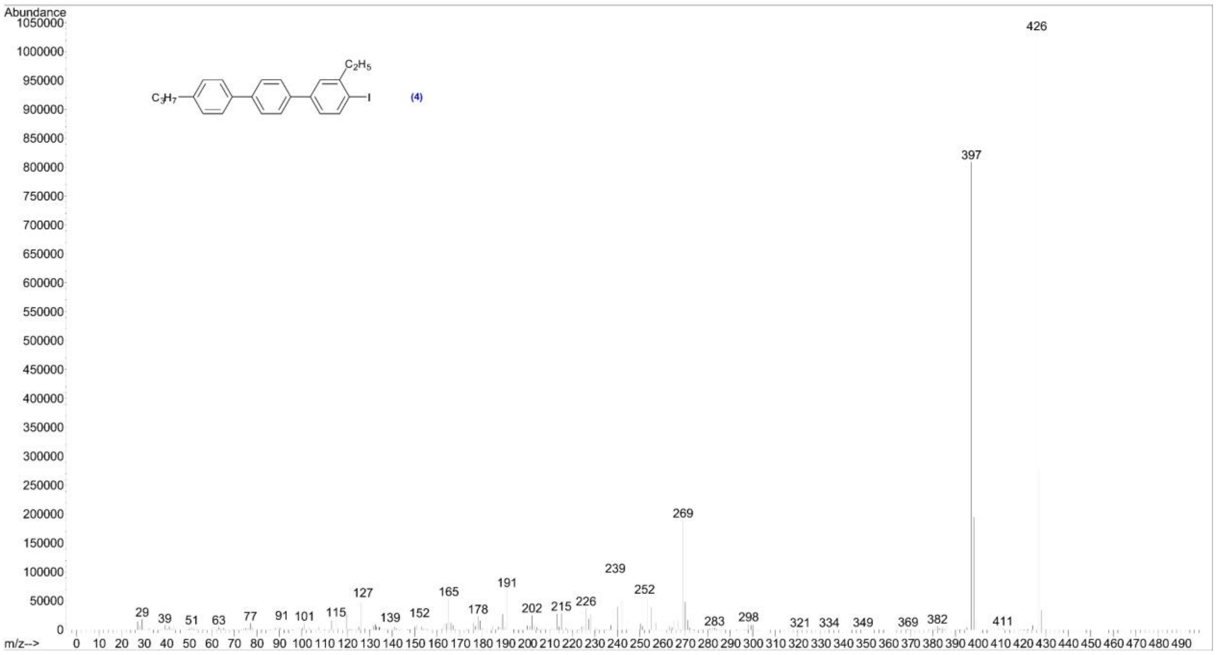

Figure S23. MS spectrum of 4-iodo-3-ethyl-4’’-propylterphenyl (**4**).

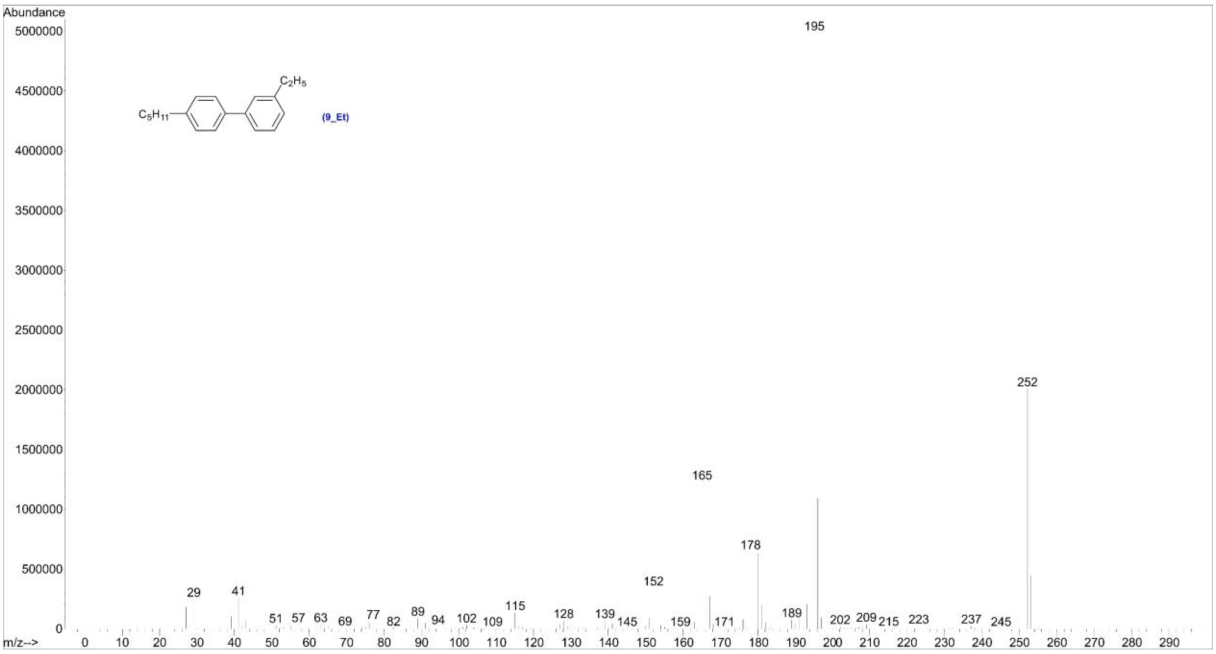

Figure S24. MS spectrum of 4'-pentyl-3-ethyllbiphenyl (**9_Et**).

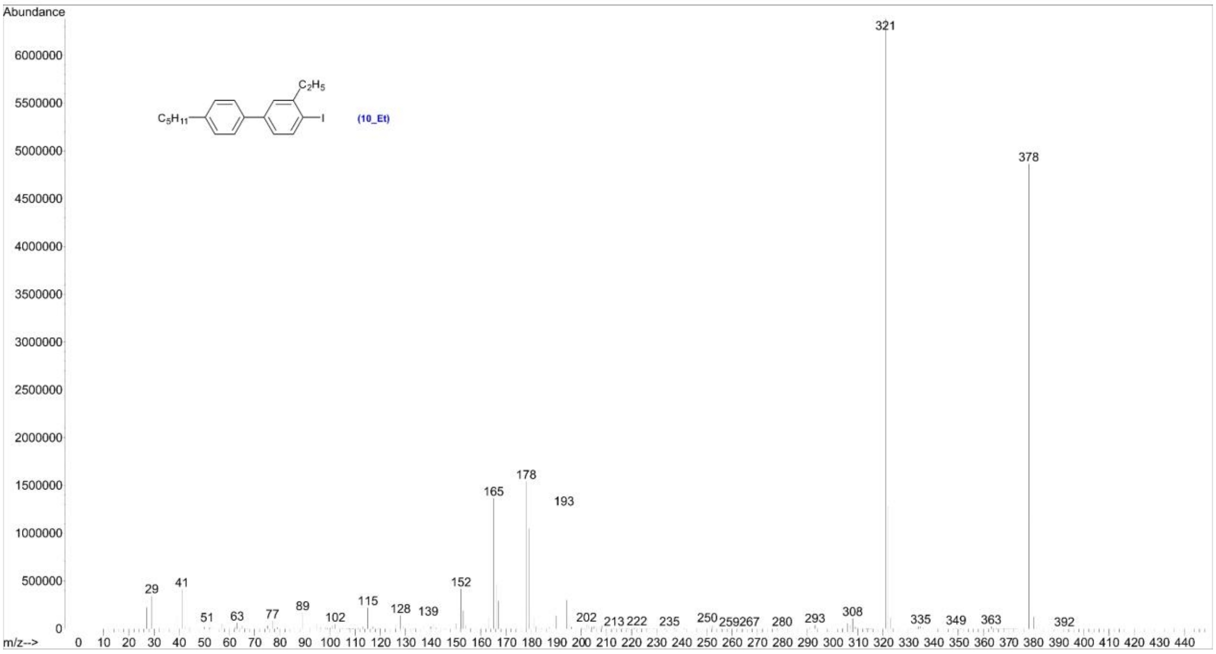

Figure S25. MS spectrum of 4-iodo-4'-pentyl-3-ethylbiphenyl (**10_Et**).

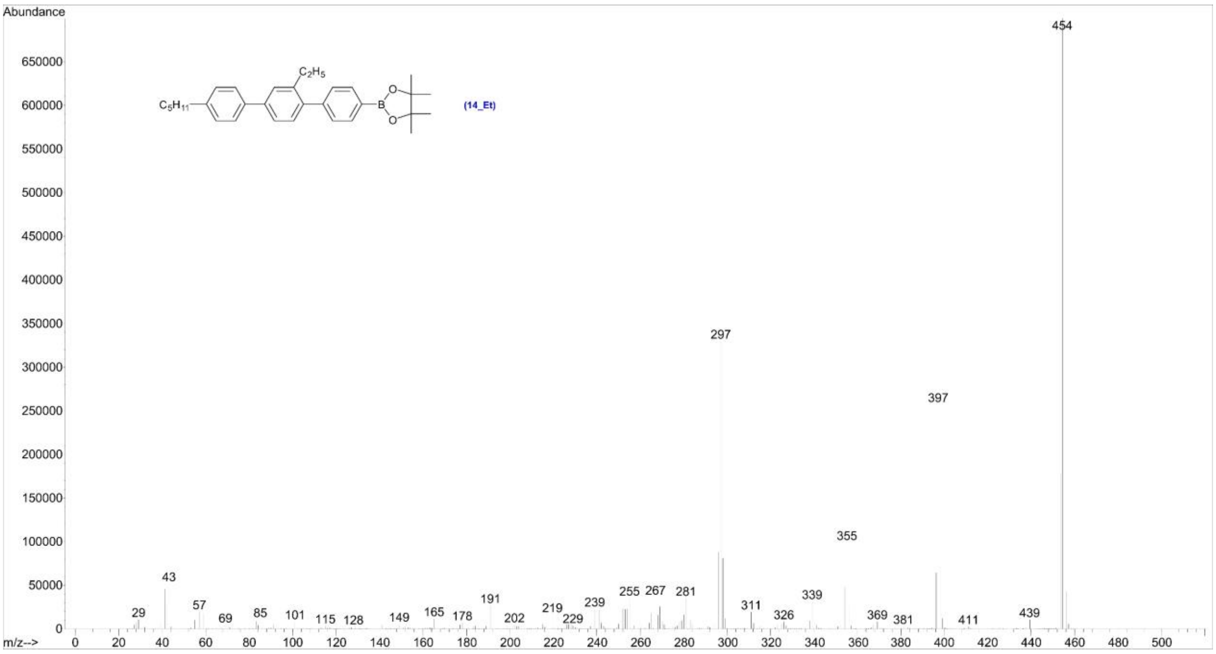

Figure S26. MS spectrum of 4,4,5,5-tetramethyl-2-(4''-pentyl-2'-ethyl-1,1':4',1''-terphenyl-4-yl)-1,3,2-dioxaborolane (**14_Et**)

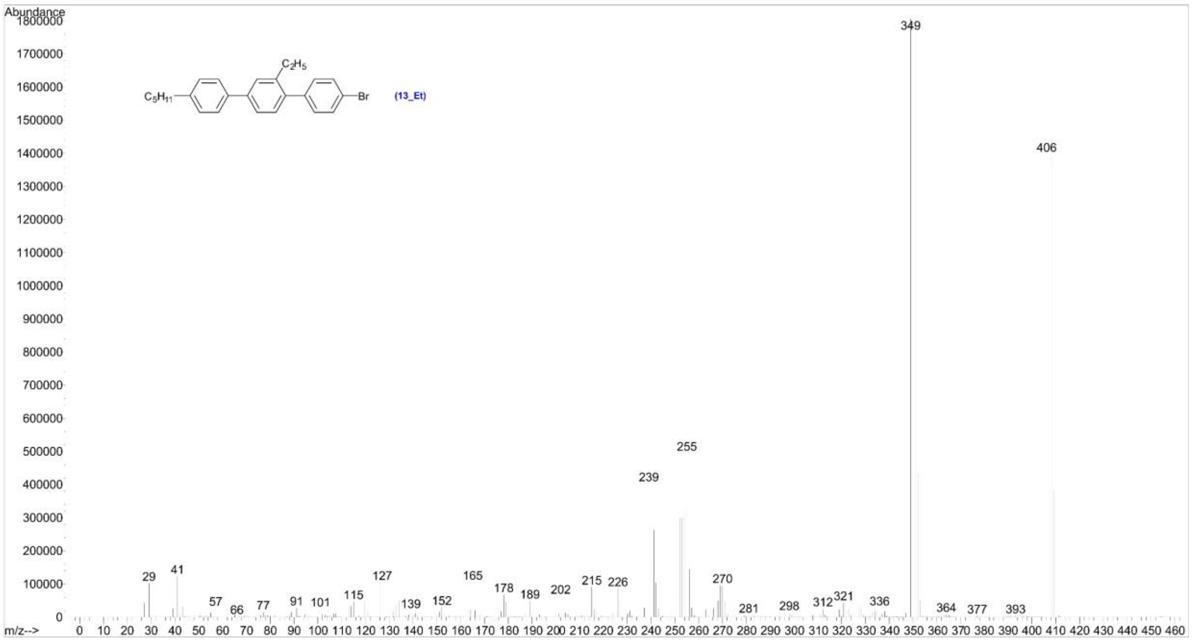

Figure S27. MS spectrum of 4-bromo-2'-ethyl-4''-pentyl-1,1':4',1''-terphenyl (**13_Et**).

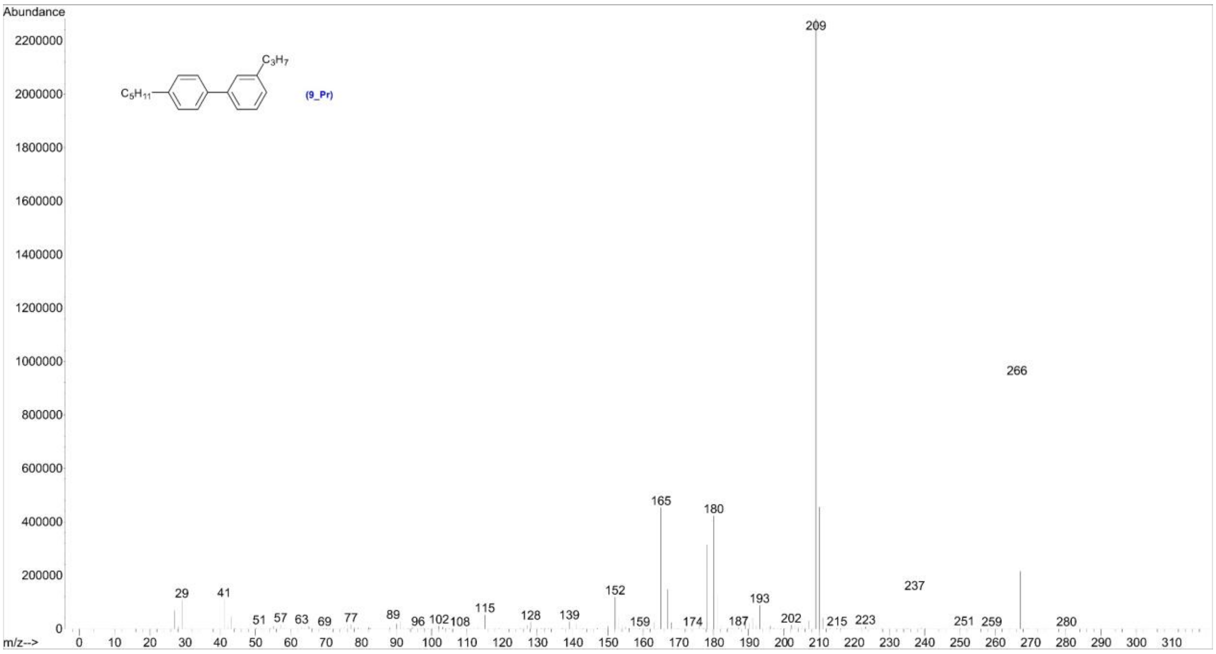

Figure S28. MS spectrum of 4'-pentyl-3-propylbiphenyl (**9_Pr**).

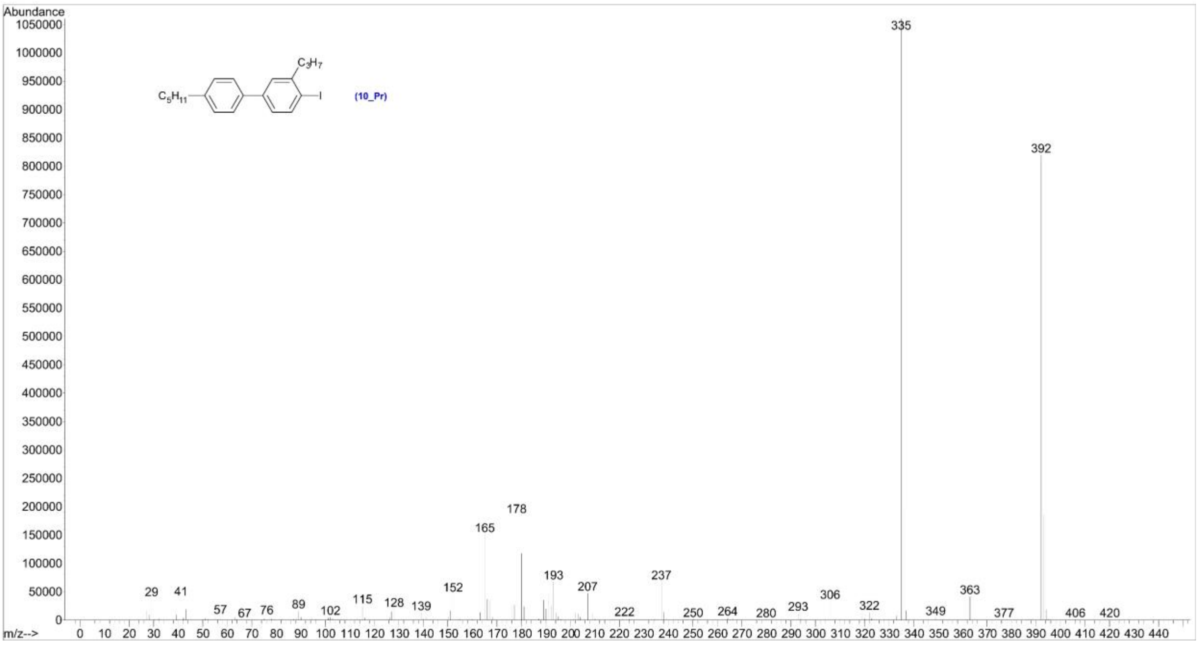

Figure S29. MS spectrum of 4-iodo-4'-pentyl-3-propylbiphenyl (**10_Pr**).

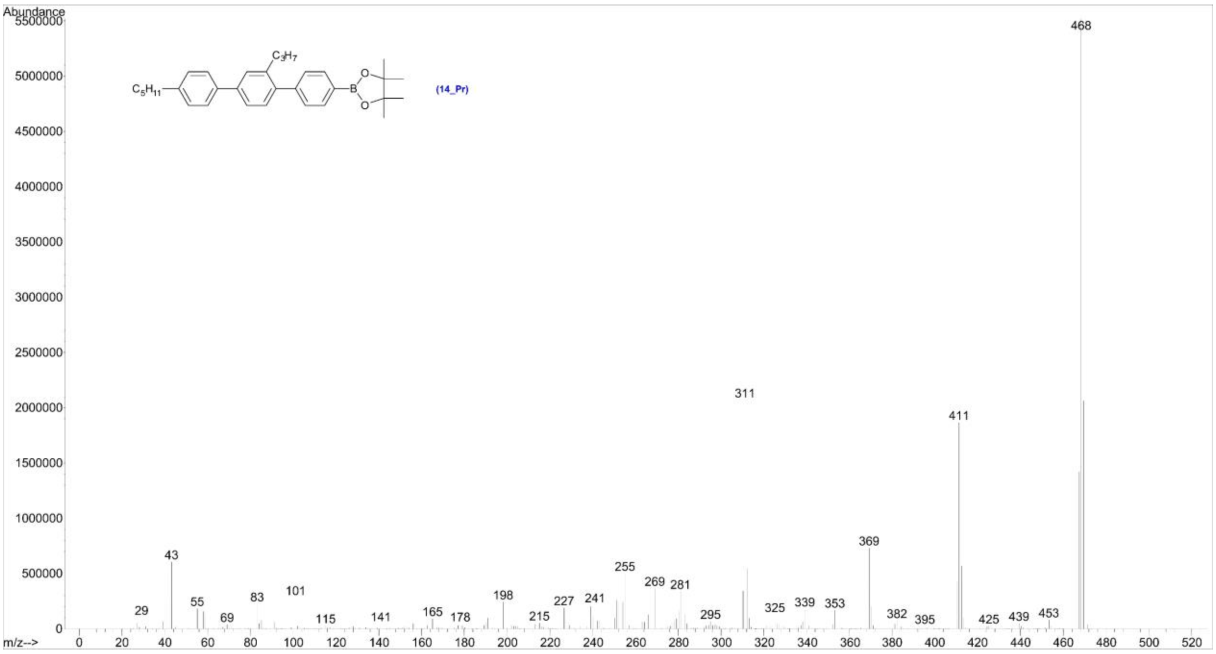

Figure S30. MS spectrum of 4,4,5,5-tetramethyl-2-(4''-pentyl-2'-propyl-1,1':4',1''-terphenyl-4-yl)-1,3,2-dioxaborolane (**14_Pr**)

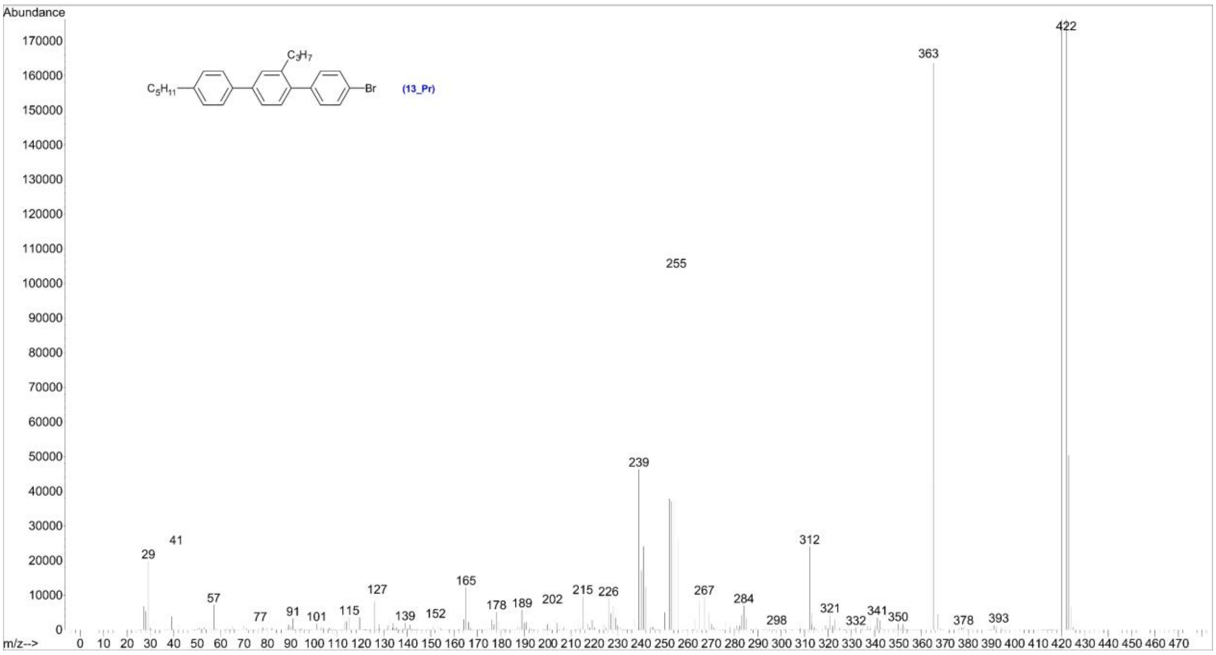

Figure S31. MS spectrum of 4-bromo-2'-propyl-4''-pentyl-1,1':4',1''-terphenyl (**13_Pr**).

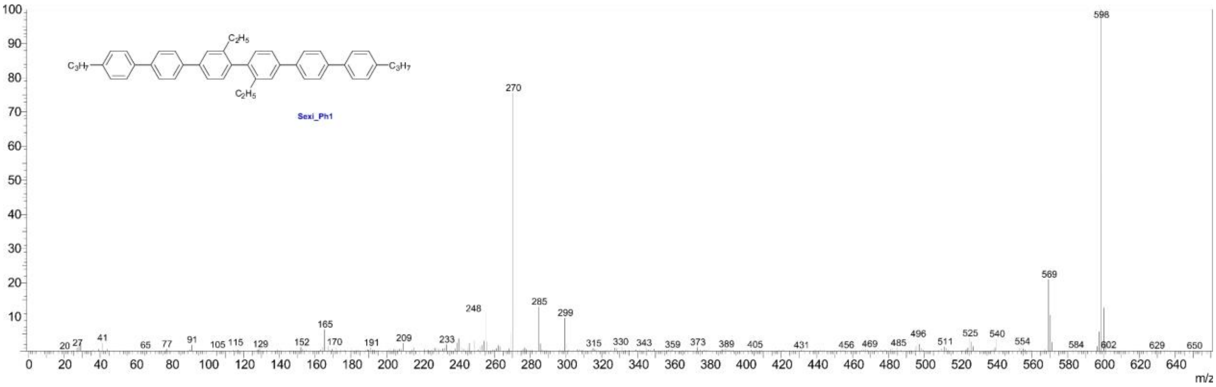

Figure S32. MS spectrum of **Sexi_Ph1**.

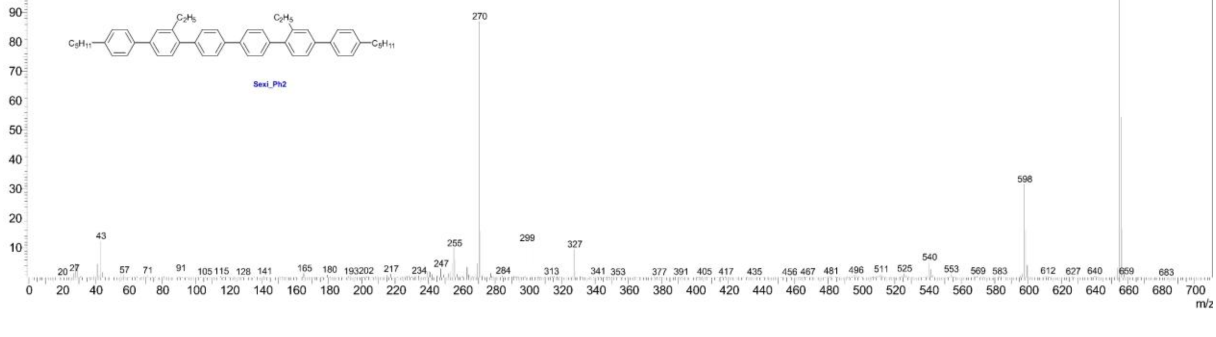

Figure S33. MS spectrum of **Sexi_Ph2**.

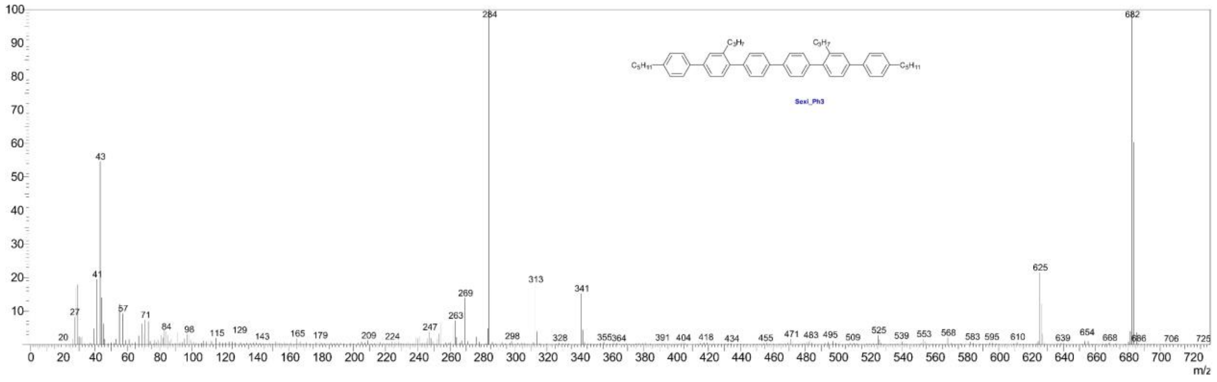

Figure S34. MS spectrum of **Sexi_Ph3**.

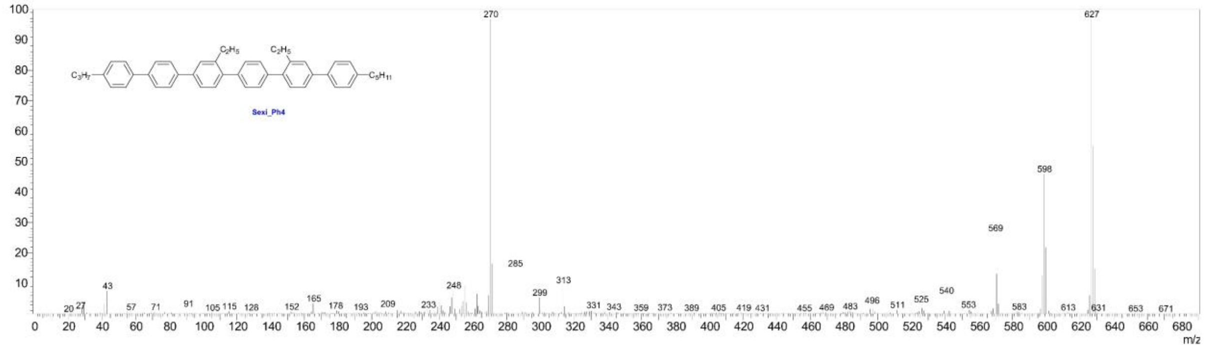

Figure S35. MS spectrum of **Sexi_Ph4**.

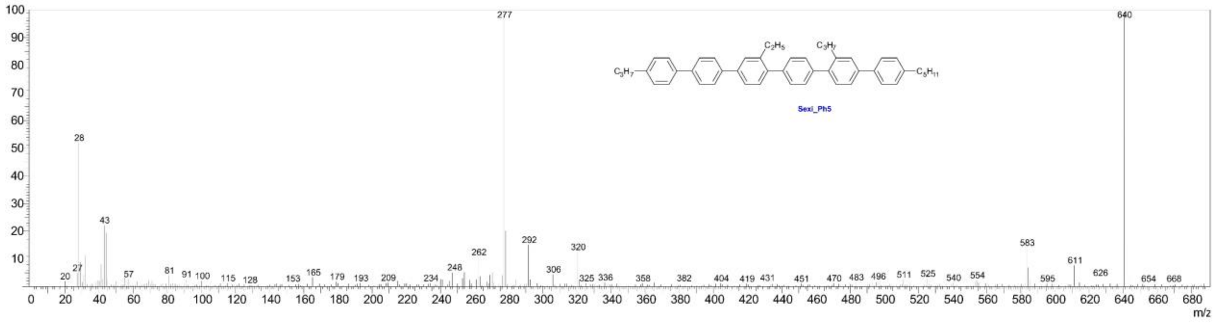

Figure S36. MS spectrum of **Sexi_Ph5**.

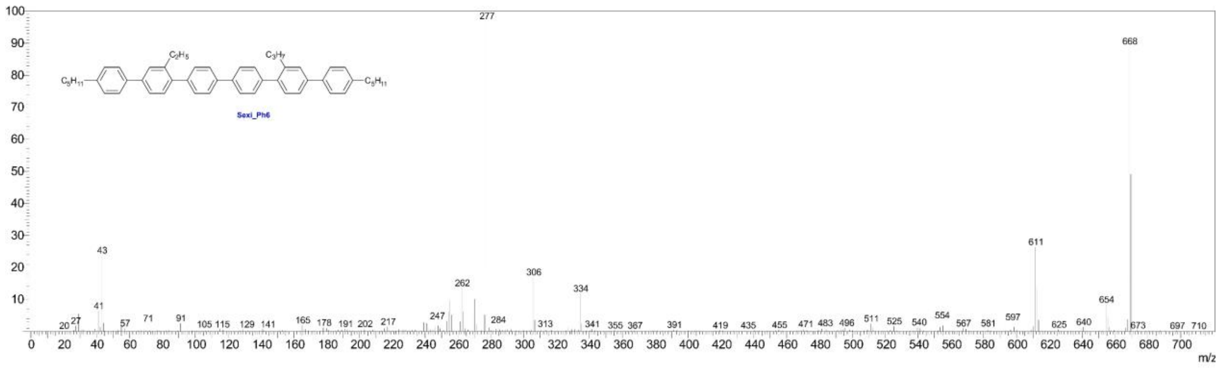

Figure S37. MS spectrum of **Sexi_Ph6**.

4. Differential scanning calorimetry (DSC) data

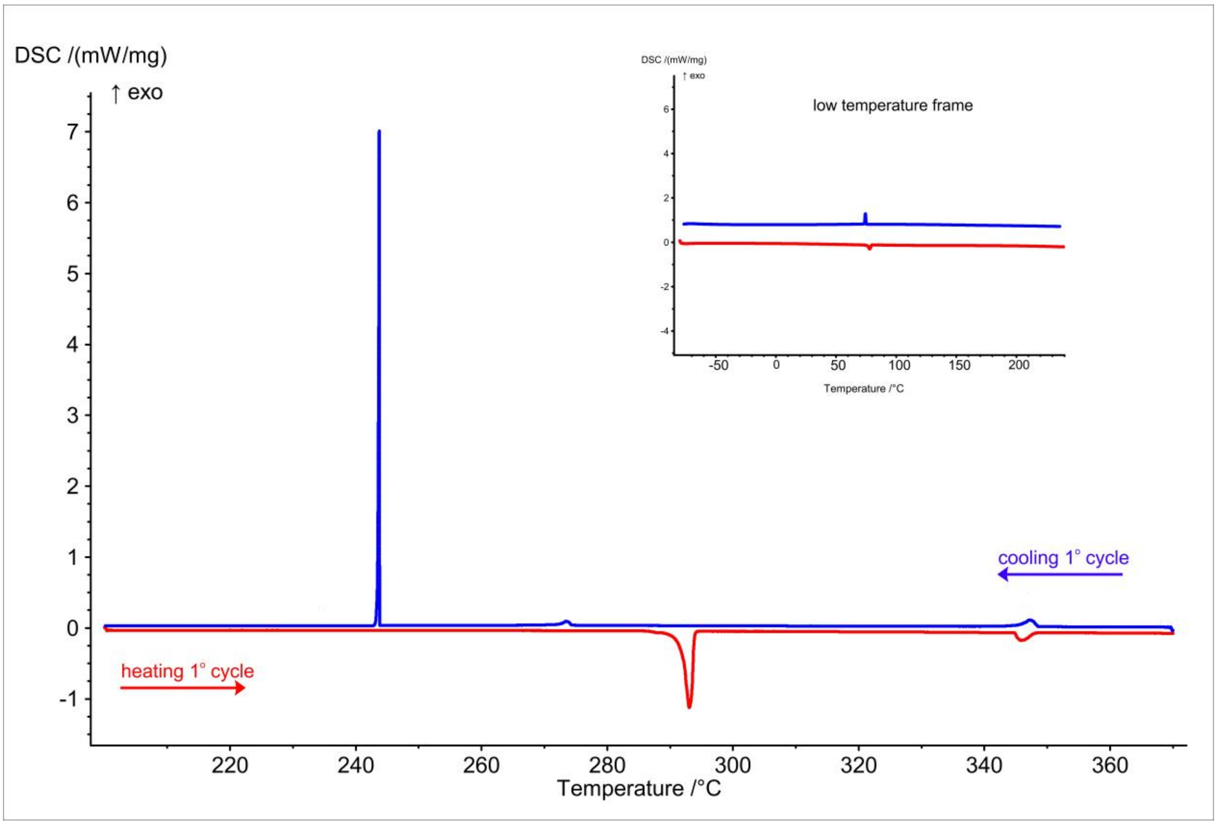

Figure S38. DSC of **Sexi_Ph1**.

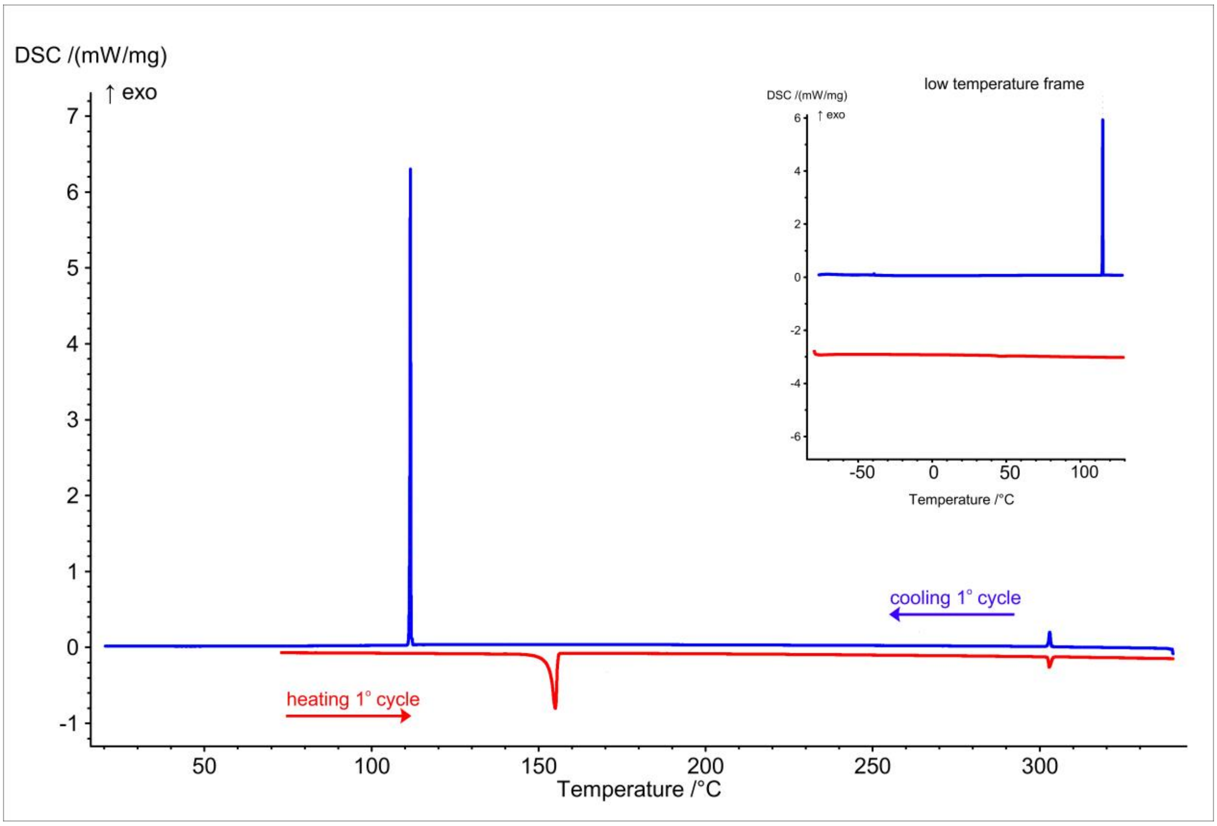

Figure S39. DSC of **Sexi_Ph2**.

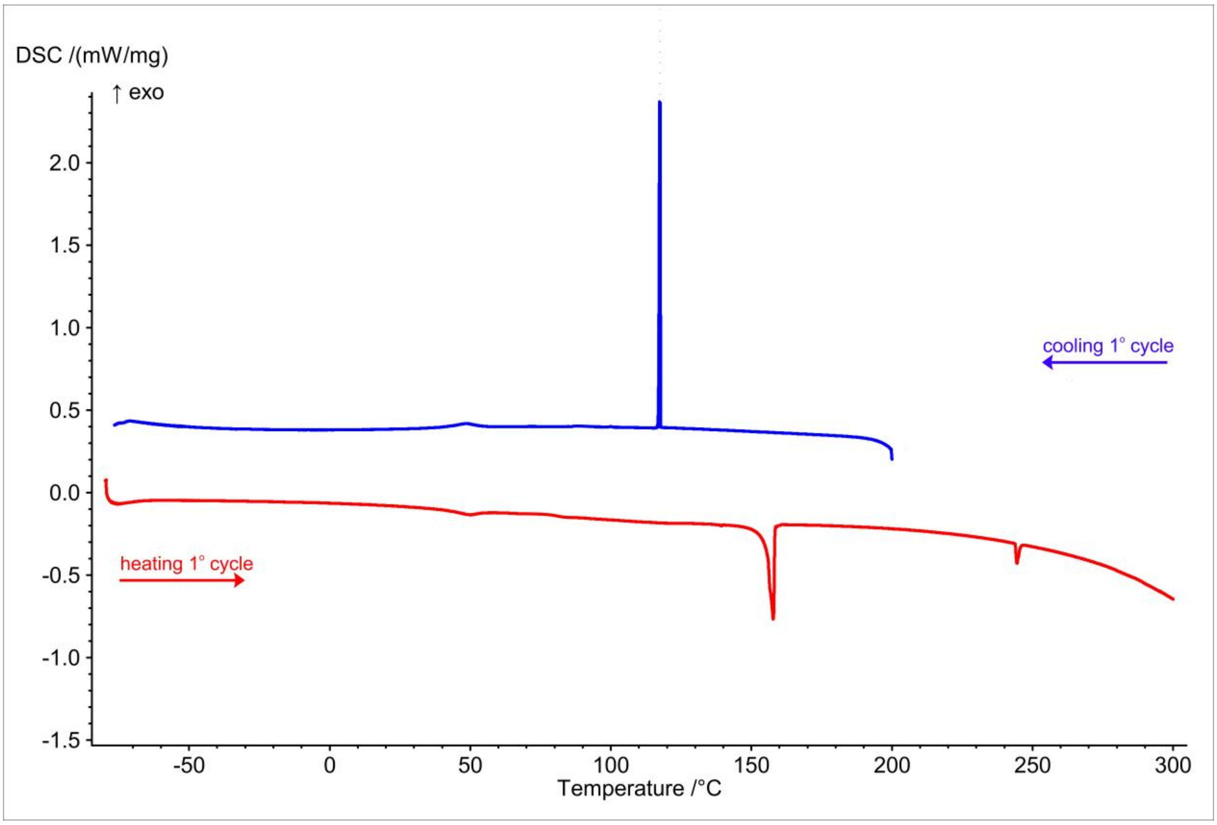

Figure S40. DSC of **Sexi_Ph3**.

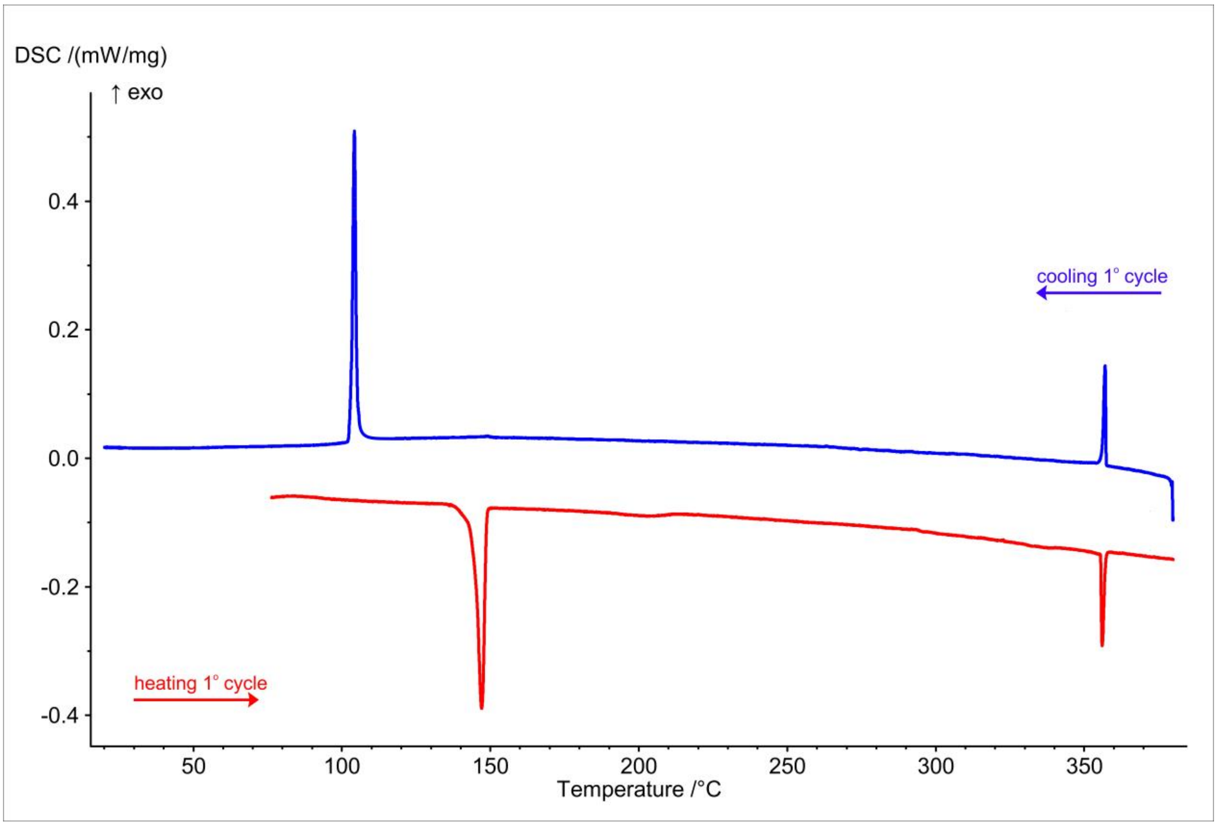

Figure S41. DSC of **Sexi_Ph4**.

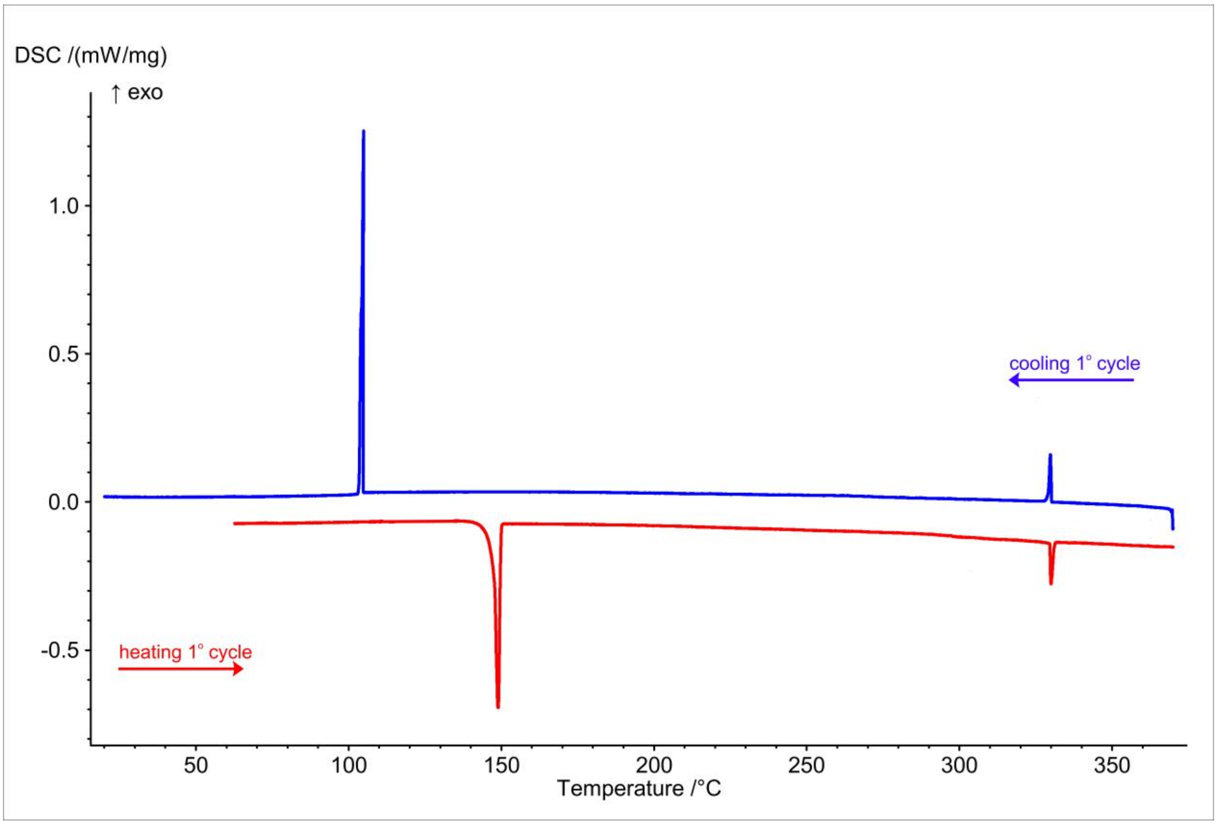

Figure S42. DSC of **Sexi_Ph5**.

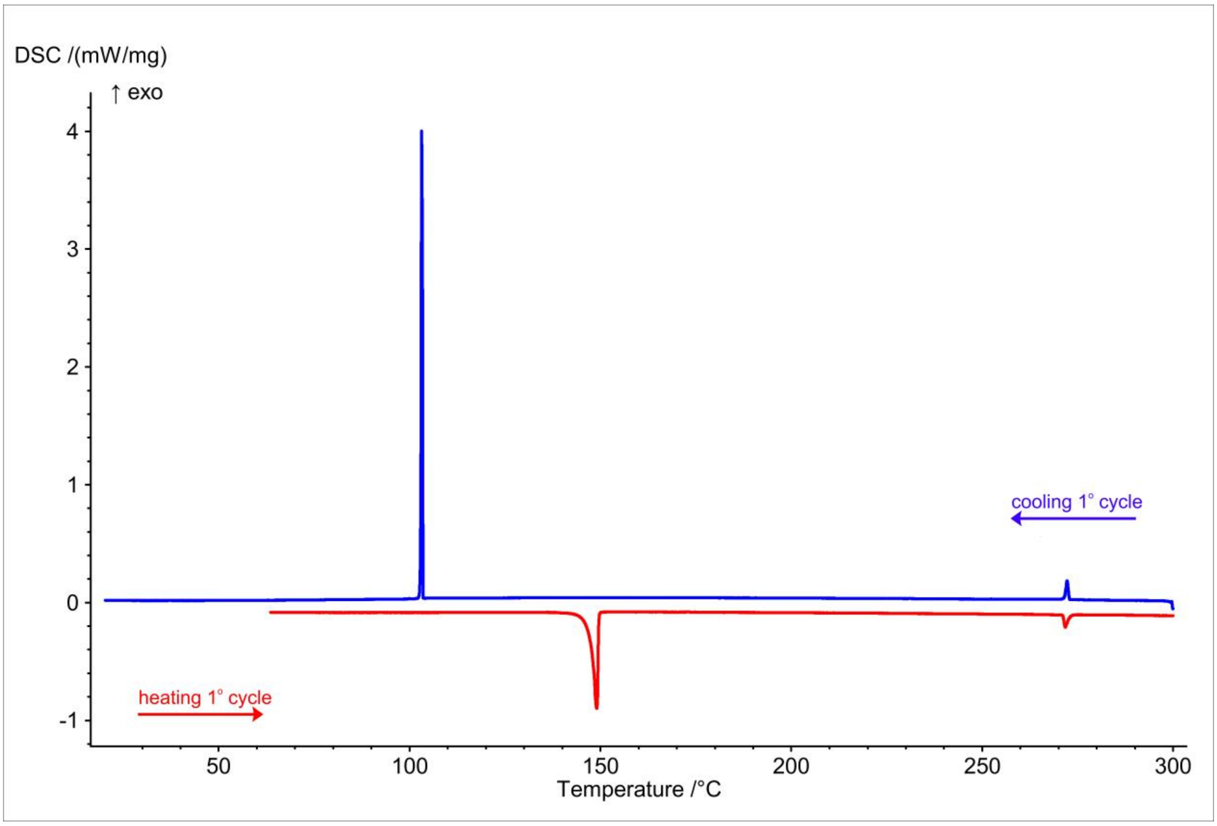

Figure S43. DSC of **Sexi_Ph6**.

5. Computational investigation

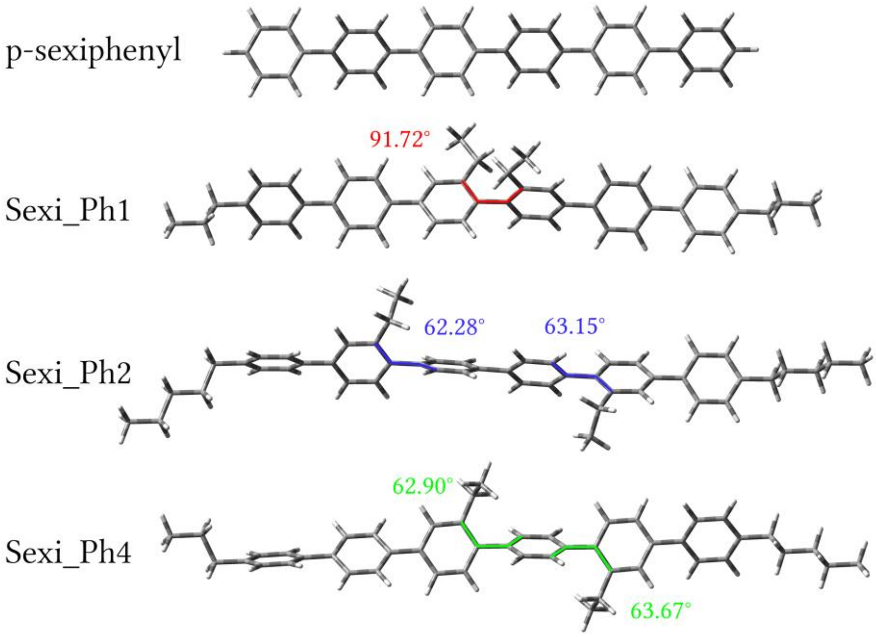

Figure S44. Optimized ground state geometry of p-sexiphenyl[1], **Sexi_Ph1**, **Sexi_Ph2**, and **Sexi_Ph4**.

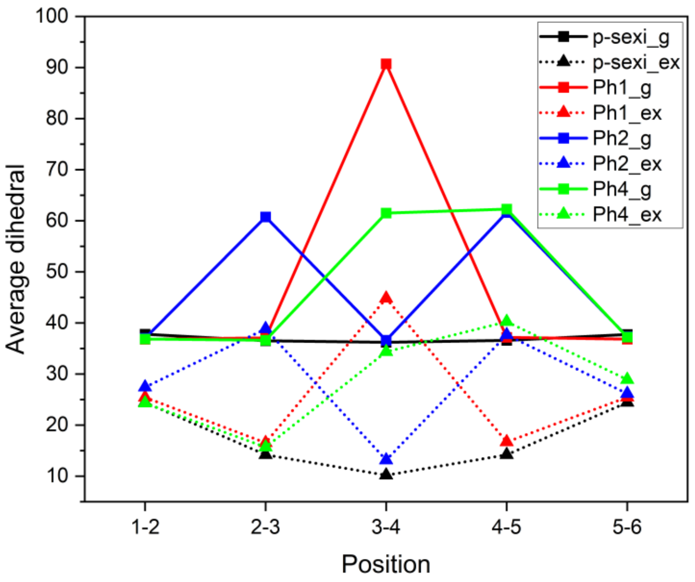

Figure S45. Average dihedrals in ground (■) and excited (▲)state for p-sexiphenyl[1], **Sexi_Ph1**, **Sexi_Ph2**, and **Sexi_Ph4**.

Table S1. Calculated values of isotropic polarizability(α), polarizability anisotropy(Δα), and XX, YY, and ZZ polarizability tensor elements.

Compound	α	Δα	XX	YY	XX
p-sexiphenyl	461	680	895	338	150
Sexi_Ph1	574	694	1032	313	376
Sexi_Ph2	618	703	1082	369	405
Sexi_Ph3	641	697	1099	400	424
Sexi_Ph4	594	694	1054	394	335
Sexi_Ph5	606	691	1064	403	350
Sexi_Ph6	630	704	1096	440	355

All values in expressed in $Bohr^3$(with 1 Bohr = 0.52917 Å).

Table S2. Ground state Cartesian coordinates of Sexi_Ph1

C	11.504327000000	-1.280014000000	0.904140000000
C	10.106850000000	-1.249348000000	0.871685000000
C	9.417387000000	-0.207170000000	0.218837000000
C	10.185671000000	0.802315000000	-0.396067000000
C	11.583020000000	0.768313000000	-0.359550000000
C	12.270376000000	-0.274014000000	0.287997000000
H	12.008255000000	-2.089315000000	1.427364000000
H	9.544334000000	-2.025083000000	1.382789000000
H	9.685506000000	1.608299000000	-0.924884000000
H	12.148888000000	1.559534000000	-0.845810000000
C	7.931731000000	-0.171359000000	0.184682000000
C	7.226647000000	1.043222000000	0.306694000000
C	7.176101000000	-1.351258000000	0.028671000000
C	5.830755000000	1.076671000000	0.271341000000
H	7.776665000000	1.969213000000	0.445391000000
C	5.780154000000	-1.318321000000	-0.001467000000
H	7.687489000000	-2.302461000000	-0.085078000000
C	5.075064000000	-0.103438000000	0.118074000000
H	5.320550000000	2.032026000000	0.349626000000
H	5.229061000000	-2.248274000000	-0.104788000000
C	3.588804000000	-0.069332000000	0.081163000000
C	2.863970000000	-0.908319000000	-0.783535000000
C	2.865101000000	0.804802000000	0.917921000000
C	1.467307000000	-0.861717000000	-0.804272000000
H	3.389482000000	-1.577922000000	-1.457398000000
C	1.463432000000	0.866024000000	0.911009000000
H	3.418365000000	1.429976000000	1.609401000000
C	0.750007000000	0.015340000000	0.026404000000
H	0.918203000000	-1.510772000000	-1.481227000000
C	-0.749995000000	0.015323000000	-0.026403000000
C	-1.467271000000	-0.861654000000	0.804375000000
C	-1.463442000000	0.865890000000	-0.911102000000
C	-2.863936000000	-0.908290000000	0.783647000000
H	-0.918152000000	-1.510632000000	1.481392000000
C	-2.865109000000	0.804635000000	-0.918007000000
C	-3.588792000000	-0.069420000000	-0.081146000000

H	-3.389426000000	-1.577843000000	1.457575000000
H	-3.418385000000	1.429699000000	-1.609577000000
C	-5.075049000000	-0.103580000000	-0.118063000000
C	-5.830778000000	1.076484000000	-0.271497000000
C	-5.780105000000	-1.318469000000	0.001623000000
C	-7.226668000000	1.042984000000	-0.306879000000
H	-5.320605000000	2.031846000000	-0.349899000000
C	-7.176049000000	-1.351457000000	-0.028544000000
H	-5.228986000000	-2.248393000000	0.105068000000
C	-7.931716000000	-0.171609000000	-0.184732000000
H	-7.776711000000	1.968937000000	-0.445707000000
H	-7.687405000000	-2.302665000000	0.085323000000
C	-9.417370000000	-0.207466000000	-0.218921000000
C	-10.106786000000	-1.249771000000	-0.871600000000
C	-10.185705000000	0.802078000000	0.395842000000
C	-11.504268000000	-1.280502000000	-0.904056000000
H	-9.544243000000	-2.025573000000	-1.382573000000
C	-11.583047000000	0.768009000000	0.359326000000
H	-9.685574000000	1.608151000000	0.924554000000
C	-12.270359000000	-0.274442000000	-0.288082000000
H	-12.008159000000	-2.089888000000	-1.427183000000
H	-12.148947000000	1.559302000000	0.845432000000
C	13.784990000000	-0.332121000000	0.288223000000
H	14.137193000000	-0.807232000000	1.214800000000
H	14.194961000000	0.687752000000	0.283098000000
C	14.367531000000	-1.107871000000	-0.920193000000
H	13.959748000000	-2.128548000000	-0.919163000000
H	14.017636000000	-0.636285000000	-1.849295000000
C	15.903952000000	-1.158992000000	-0.906607000000
H	16.290481000000	-1.712175000000	-1.770904000000
H	16.333669000000	-0.149212000000	-0.935795000000
H	16.275365000000	-1.652993000000	0.000687000000
C	-13.784978000000	-0.332568000000	-0.288320000000
H	-14.137095000000	-0.808837000000	-1.214334000000
H	-14.194967000000	0.687311000000	-0.284506000000
C	-14.367605000000	-1.106807000000	0.921008000000
H	-13.959843000000	-2.127493000000	0.921280000000
H	-14.017782000000	-0.634093000000	1.849564000000
C	-15.904027000000	-1.157916000000	0.907367000000
H	-16.275376000000	-1.653024000000	0.000651000000
H	-16.290651000000	-1.710024000000	1.772310000000
H	-16.333725000000	-0.148092000000	0.935268000000
C	0.697412000000	1.801918000000	1.840777000000
H	0.017431000000	2.418800000000	1.235761000000
H	0.036734000000	1.197738000000	2.478889000000
C	1.543358000000	2.723251000000	2.732231000000
H	2.183060000000	2.153860000000	3.417095000000
H	2.186185000000	3.385601000000	2.139678000000
H	0.888082000000	3.355671000000	3.342037000000
C	-0.697437000000	1.801741000000	-1.840924000000
H	-0.018010000000	2.419199000000	-1.235858000000
H	-0.036196000000	1.197598000000	-2.478475000000
C	-1.543399000000	2.722319000000	-2.733141000000
H	-2.182726000000	2.152331000000	-3.417861000000
H	-2.186602000000	3.384821000000	-2.141167000000

H	-0.888133000000	3.354594000000	-3.343108000000

Table S3. Excited state Cartesian coordinates of Sexi_Ph1

C	-11.420880000000	-1.305525000000	0.885754000000
C	-10.036491000000	-1.198554000000	0.868337000000
C	-9.388474000000	-0.131154000000	0.205011000000
C	-10.217122000000	0.819801000000	-0.434280000000
C	-11.600807000000	0.706238000000	-0.409736000000
C	-12.237441000000	-0.358326000000	0.247926000000
H	-11.882545000000	-2.145371000000	1.400065000000
H	-9.443579000000	-1.970218000000	1.348558000000
H	-9.768811000000	1.679805000000	-0.921149000000
H	-12.205245000000	1.467124000000	-0.898330000000
C	-7.927954000000	-0.013111000000	0.184758000000
C	-7.248691000000	0.679526000000	-0.848965000000
C	-7.117072000000	-0.584820000000	1.196964000000
C	-5.873276000000	0.785367000000	-0.875696000000
H	-7.819587000000	1.117768000000	-1.661290000000
C	-5.742285000000	-0.468078000000	1.183277000000
H	-7.586231000000	-1.106607000000	2.024874000000
C	-5.053079000000	0.218985000000	0.142442000000
H	-5.416482000000	1.341440000000	-1.686690000000
H	-5.177807000000	-0.940327000000	1.979514000000
C	-3.608021000000	0.331452000000	0.125833000000
C	-2.817809000000	0.087500000000	1.285737000000
C	-2.893923000000	0.705624000000	-1.047963000000
C	-1.449845000000	0.205901000000	1.250242000000
H	-3.292849000000	-0.178832000000	2.223126000000
C	-1.515724000000	0.805364000000	-1.120442000000
H	-3.462587000000	0.913774000000	-1.945373000000
C	-0.730177000000	0.532564000000	0.061396000000
H	-0.886052000000	-0.023300000000	2.147036000000
C	0.724298000000	0.507127000000	0.078785000000
C	1.424733000000	-0.171352000000	-0.963822000000
C	1.525880000000	1.075114000000	1.138582000000
C	2.786972000000	-0.347073000000	-0.955998000000
H	0.848007000000	-0.624609000000	-1.761687000000
C	2.898909000000	0.906568000000	1.105509000000
C	3.591868000000	0.186446000000	0.091227000000
H	3.246800000000	-0.886964000000	-1.775993000000
H	3.479844000000	1.339618000000	1.909932000000
C	5.031364000000	0.017650000000	0.116419000000
C	5.878504000000	0.819966000000	0.934067000000
C	5.687277000000	-0.966083000000	-0.678767000000
C	7.248548000000	0.656818000000	0.947727000000
H	5.448134000000	1.603149000000	1.548080000000
C	7.056276000000	-1.135975000000	-0.649373000000
H	5.100258000000	-1.624610000000	-1.309188000000
C	7.894352000000	-0.329964000000	0.161103000000
H	7.840456000000	1.287169000000	1.603564000000
H	7.500316000000	-1.891821000000	-1.289191000000
C	9.348933000000	-0.506948000000	0.183713000000
C	9.948841000000	-1.746897000000	-0.136040000000
C	10.219025000000	0.552109000000	0.530833000000

C	11.327327000000	-1.911699000000	-0.109509000000
H	9.321588000000	-2.601433000000	-0.368335000000
C	11.596673000000	0.378977000000	0.552390000000
H	9.808727000000	1.531314000000	0.755413000000
C	12.185554000000	-0.854662000000	0.232919000000
H	11.750755000000	-2.885549000000	-0.344761000000
H	12.233870000000	1.219759000000	0.817073000000
C	-13.740706000000	-0.501643000000	0.233762000000
H	-14.206293000000	0.492369000000	0.219566000000
H	-14.074396000000	-0.988633000000	1.159176000000
C	-14.263524000000	-1.312451000000	-0.971259000000
H	-13.928582000000	-0.829286000000	-1.897690000000
H	-13.797407000000	-2.305693000000	-0.961261000000
C	-15.788118000000	-1.451781000000	-0.977344000000
H	-16.131091000000	-2.030233000000	-1.841167000000
H	-16.144268000000	-1.959972000000	-0.073998000000
H	-16.276516000000	-0.471505000000	-1.018161000000
C	13.685773000000	-1.026911000000	0.217355000000
H	13.942273000000	-2.055575000000	0.502143000000
H	14.140880000000	-0.372262000000	0.971803000000
C	14.319729000000	-0.718885000000	-1.155966000000
H	13.864031000000	-1.369966000000	-1.912499000000
H	14.062564000000	0.307983000000	-1.444599000000
C	15.840268000000	-0.897126000000	-1.162039000000
H	16.120814000000	-1.925223000000	-0.906204000000
H	16.263024000000	-0.672429000000	-2.146526000000
H	16.321015000000	-0.233604000000	-0.434196000000
C	-0.866014000000	1.280856000000	-2.413928000000
H	0.085313000000	1.761598000000	-2.168024000000
H	-0.600914000000	0.417528000000	-3.040707000000
C	-1.699930000000	2.251881000000	-3.261543000000
H	-2.591992000000	1.780347000000	-3.684908000000
H	-2.023707000000	3.116263000000	-2.673229000000
H	-1.102409000000	2.618504000000	-4.102111000000
C	0.901570000000	1.922976000000	2.239790000000
H	-0.033403000000	2.347698000000	1.862984000000
H	0.609676000000	1.284423000000	3.085677000000
C	1.775080000000	3.064661000000	2.778656000000
H	2.652507000000	2.701592000000	3.322391000000
H	2.125623000000	3.713214000000	1.969578000000
H	1.196484000000	3.676985000000	3.477432000000

Table S4. Ground state Cartesian coordinates of Sexi_Ph2

C	11.526001000000	-1.408716000000	-0.511672000000
C	10.131480000000	-1.399466000000	-0.409997000000
C	9.391754000000	-0.226611000000	-0.663869000000
C	10.106206000000	0.933147000000	-1.026448000000
C	11.500768000000	0.918928000000	-1.128123000000
C	12.238684000000	-0.250225000000	-0.870039000000
H	12.068755000000	-2.332223000000	-0.323693000000
H	9.608716000000	-2.317917000000	-0.159878000000
H	9.567084000000	1.857888000000	-1.209876000000
H	12.024923000000	1.830138000000	-1.406879000000
C	7.908181000000	-0.213504000000	-0.559737000000

C	7.118626000000	0.509805000000	-1.473656000000
C	7.244630000000	-0.924243000000	0.457404000000
C	5.728210000000	0.519901000000	-1.352945000000
H	7.590782000000	1.050066000000	-2.288415000000
C	5.845956000000	-0.933928000000	0.592259000000
H	7.835546000000	-1.477467000000	1.183063000000
C	5.067438000000	-0.185143000000	-0.327297000000
H	5.133901000000	1.091353000000	-2.060526000000
C	3.575461000000	-0.110573000000	-0.260949000000
C	2.913212000000	0.482686000000	0.831700000000
C	2.786838000000	-0.586629000000	-1.326148000000
C	1.520071000000	0.585869000000	0.862036000000
H	3.497886000000	0.894299000000	1.649393000000
C	1.392553000000	-0.486811000000	-1.295056000000
H	3.272868000000	-1.055414000000	-2.177183000000
C	0.728865000000	0.099910000000	-0.199251000000
H	1.041951000000	1.078904000000	1.703234000000
H	0.812191000000	-0.893048000000	-2.118084000000
C	-0.753400000000	0.208387000000	-0.164394000000
C	-1.491843000000	0.498243000000	-1.328957000000
C	-1.469782000000	0.020523000000	1.035642000000
C	-2.885837000000	0.600305000000	-1.293749000000
H	-0.969673000000	0.672675000000	-2.264967000000
C	-2.862597000000	0.126037000000	1.070540000000
H	-0.933887000000	-0.243220000000	1.942591000000
C	-3.599532000000	0.423684000000	-0.092602000000
H	-3.429943000000	0.838520000000	-2.203393000000
H	-3.389366000000	-0.054904000000	2.003099000000
C	-5.092840000000	0.494185000000	-0.078665000000
C	-5.814897000000	1.452444000000	0.678564000000
C	-5.814646000000	-0.433154000000	-0.856433000000
C	-7.219093000000	1.424756000000	0.639738000000
C	-7.210066000000	-0.440696000000	-0.883968000000
H	-5.264583000000	-1.177413000000	-1.425540000000
C	-7.943053000000	0.491795000000	-0.125598000000
H	-7.764406000000	2.179506000000	1.200873000000
H	-7.731261000000	-1.193842000000	-1.466902000000
C	-9.430133000000	0.493488000000	-0.133966000000
C	-10.151457000000	0.239562000000	-1.318225000000
C	-10.166809000000	0.753442000000	1.039261000000
C	-11.549629000000	0.243497000000	-1.325782000000
H	-9.613093000000	0.066462000000	-2.245417000000
C	-11.565120000000	0.757852000000	1.026687000000
H	-9.640782000000	0.932904000000	1.972302000000
C	-12.284371000000	0.500210000000	-0.154129000000
H	-12.078943000000	0.057860000000	-2.257516000000
H	-12.106566000000	0.958629000000	1.948254000000
C	13.752986000000	-0.252576000000	-0.939879000000
H	14.088706000000	0.467124000000	-1.699657000000
H	14.105858000000	-1.241068000000	-1.266222000000
C	14.427987000000	0.096534000000	0.410174000000
H	14.077859000000	1.085403000000	0.741091000000
H	14.094512000000	-0.621336000000	1.174004000000
C	15.965271000000	0.091856000000	0.332861000000
H	16.293770000000	0.809477000000	-0.435517000000

H	16.310309000000	-0.898935000000	-0.002727000000
C	16.646232000000	0.437360000000	1.670688000000
H	16.301314000000	1.426970000000	2.005939000000
H	16.318005000000	-0.279599000000	2.438117000000
C	18.182086000000	0.430968000000	1.586184000000
H	18.539400000000	1.162078000000	0.849362000000
H	18.636671000000	0.680153000000	2.552601000000
H	18.556210000000	-0.555989000000	1.284418000000
C	-13.799832000000	0.463073000000	-0.156207000000
H	-14.187524000000	1.165297000000	0.595155000000
H	-14.176108000000	0.806239000000	-1.130257000000
C	-14.377288000000	-0.945176000000	0.132777000000
H	-14.003724000000	-1.292976000000	1.107173000000
H	-13.991257000000	-1.651765000000	-0.616675000000
C	-15.916198000000	-0.976685000000	0.128102000000
H	-16.297626000000	-0.265200000000	0.877511000000
H	-16.284959000000	-0.623812000000	-0.848036000000
C	-16.500418000000	-2.373029000000	0.413989000000
H	-16.131900000000	-2.725460000000	1.389016000000
H	-16.119174000000	-3.083609000000	-0.334626000000
C	-18.038409000000	-2.397316000000	0.407660000000
H	-18.423180000000	-3.403348000000	0.613938000000
H	-18.446508000000	-1.719858000000	1.168985000000
H	-18.433692000000	-2.080537000000	-0.566261000000
C	5.226765000000	-1.808156000000	1.672314000000
H	5.950900000000	-1.926930000000	2.488848000000
H	4.343323000000	-1.328137000000	2.104110000000
C	4.824932000000	-3.206684000000	1.147663000000
H	4.070634000000	-3.125635000000	0.356994000000
H	5.693152000000	-3.734020000000	0.734733000000
H	4.405659000000	-3.817167000000	1.956811000000
C	-5.133246000000	2.573147000000	1.449244000000
H	-5.797558000000	2.899775000000	2.259963000000
H	-4.212737000000	2.217570000000	1.921934000000
C	-4.794903000000	3.788842000000	0.554703000000
H	-4.098725000000	3.505089000000	-0.242534000000
H	-5.699948000000	4.193552000000	0.086354000000
H	-4.328538000000	4.586213000000	1.146225000000

Table S5. Excited state Cartesian coordinates of Sexi_Ph2

C	-11.523609000000	-1.515095000000	0.104767000000
C	-10.140120000000	-1.503208000000	-0.019202000000
C	-9.355855000000	-0.505178000000	0.600386000000
C	-10.042381000000	0.475131000000	1.350388000000
C	-11.426584000000	0.457918000000	1.466990000000
C	-12.200609000000	-0.535536000000	0.847951000000
H	-12.093448000000	-2.309363000000	-0.371990000000
H	-9.653833000000	-2.301081000000	-0.571095000000
H	-9.483367000000	1.277522000000	1.821040000000
H	-11.921328000000	1.234186000000	2.046191000000
C	-7.892102000000	-0.486820000000	0.478487000000
C	-7.070831000000	0.077808000000	1.481182000000

C	-7.233313000000	-1.044297000000	-0.639005000000
C	-5.698261000000	0.091324000000	1.346273000000
H	-7.518358000000	0.491177000000	2.379219000000
C	-5.852925000000	-1.039199000000	-0.808707000000
H	-7.834109000000	-1.498827000000	-1.421451000000
C	-5.032795000000	-0.424796000000	0.199670000000
H	-5.101033000000	0.560846000000	2.121277000000
C	-3.582561000000	-0.298392000000	0.135153000000
C	-2.872135000000	0.039669000000	-1.053932000000
C	-2.789838000000	-0.440382000000	1.313027000000
C	-1.504951000000	0.193037000000	-1.069806000000
H	-3.426862000000	0.263581000000	-1.957435000000
C	-1.421489000000	-0.303221000000	1.298509000000
H	-3.277105000000	-0.725951000000	2.240218000000
C	-0.702951000000	0.016102000000	0.102099000000
H	-1.036148000000	0.514475000000	-1.993190000000
H	-0.876750000000	-0.489176000000	2.217243000000
C	0.728853000000	0.162689000000	0.079962000000
C	1.506960000000	0.260664000000	1.278142000000
C	1.471492000000	0.206793000000	-1.142787000000
C	2.874318000000	0.400894000000	1.250753000000
H	1.008635000000	0.271460000000	2.240954000000
C	2.837741000000	0.362657000000	-1.166530000000
H	0.956596000000	0.055421000000	-2.084976000000
C	3.608270000000	0.483337000000	0.028595000000
H	3.407194000000	0.515146000000	2.189657000000
H	3.347072000000	0.308152000000	-2.121416000000
C	5.057288000000	0.605949000000	0.041631000000
C	5.828401000000	1.381030000000	-0.895580000000
C	5.781893000000	-0.087038000000	1.053110000000
C	7.214846000000	1.349555000000	-0.812420000000
C	7.158892000000	-0.091236000000	1.113788000000
H	5.226471000000	-0.697571000000	1.757629000000
C	7.930702000000	0.618328000000	0.163080000000
H	7.774103000000	1.977665000000	-1.499949000000
H	7.651810000000	-0.697208000000	1.867060000000
C	9.396618000000	0.610799000000	0.196504000000
C	10.106832000000	0.386150000000	1.397614000000
C	10.164235000000	0.830242000000	-0.969555000000
C	11.495275000000	0.382038000000	1.425992000000
H	9.560350000000	0.252180000000	2.325584000000
C	11.552525000000	0.822653000000	-0.932679000000
H	9.662382000000	0.977033000000	-1.920493000000
C	12.252084000000	0.598143000000	0.263495000000
H	12.007549000000	0.222006000000	2.371964000000
H	12.109986000000	0.987962000000	-1.851806000000
C	-13.708082000000	-0.527765000000	0.943709000000
H	-14.013859000000	-0.103533000000	1.908568000000
H	-14.083649000000	-1.558847000000	0.923491000000
C	-14.384188000000	0.271775000000	-0.190104000000
H	-14.006670000000	1.302836000000	-0.174704000000
H	-14.076797000000	-0.149047000000	-1.156689000000
C	-15.914040000000	0.279898000000	-0.092798000000
H	-16.215040000000	0.697840000000	0.878665000000
H	-16.285319000000	-0.755019000000	-0.104098000000
C	-16.593212000000	1.072855000000	-1.216360000000
H	-16.221727000000	2.106457000000	-1.204507000000
H	-16.292431000000	0.654619000000	-2.186463000000
C	-18.121382000000	1.077954000000	-1.113812000000
H	-18.453873000000	1.522819000000	-0.169007000000
H	-18.575517000000	1.651159000000	-1.928539000000
H	-18.525207000000	0.060081000000	-1.158339000000
C	13.761331000000	0.548223000000	0.291024000000

H	14.166679000000	1.225650000000	-0.471392000000
H	14.124383000000	0.915653000000	1.259428000000
C	14.326032000000	-0.868111000000	0.051353000000
H	13.961237000000	-1.239418000000	-0.915550000000
H	13.918221000000	-1.548563000000	0.810635000000
C	15.857877000000	-0.919573000000	0.080341000000
H	16.259958000000	-0.233017000000	-0.678602000000
H	16.216780000000	-0.542420000000	1.048834000000
C	16.426059000000	-2.324276000000	-0.157096000000
H	16.067321000000	-2.700213000000	-1.124806000000
H	16.023883000000	-3.009512000000	0.601315000000
C	17.956704000000	-2.370068000000	-0.126780000000
H	18.330480000000	-3.384562000000	-0.299062000000
H	18.388157000000	-1.721606000000	-0.897796000000
H	18.344376000000	-2.033037000000	0.841334000000
C	-5.292106000000	-1.822288000000	-1.983097000000
H	-6.111768000000	-2.022652000000	-2.682009000000
H	-4.552232000000	-1.248505000000	-2.545362000000
C	-4.658478000000	-3.161053000000	-1.559348000000
H	-3.811761000000	-3.001746000000	-0.886300000000
H	-5.388759000000	-3.789883000000	-1.040264000000
H	-4.296685000000	-3.709879000000	-2.435131000000
C	5.210185000000	2.378780000000	-1.859448000000
H	5.985296000000	2.697634000000	-2.565171000000
H	4.418634000000	1.930461000000	-2.463886000000
C	4.640131000000	3.620482000000	-1.147663000000
H	3.838764000000	3.345451000000	-0.456531000000
H	5.419774000000	4.132401000000	-0.574811000000
H	4.231243000000	4.328258000000	-1.876356000000

Table S6. Ground state Cartesian coordinates of Sexi_Ph4

C	-12.605727000000	-0.397124000000	-1.035213000000
C	-11.210754000000	-0.308138000000	-1.068046000000
C	-10.446127000000	-0.435457000000	0.109597000000
C	-11.136616000000	-0.657881000000	1.318401000000
C	-12.531674000000	-0.747668000000	1.346062000000
C	-13.294282000000	-0.616429000000	0.171544000000
H	-13.167930000000	-0.309134000000	-1.961949000000
H	-10.707568000000	-0.168514000000	-2.020258000000
H	-10.578465000000	-0.740089000000	2.246365000000
H	-13.036729000000	-0.916755000000	2.294217000000
C	-8.962878000000	-0.343911000000	0.076651000000
C	-8.161001000000	-1.173272000000	0.887034000000
C	-8.306455000000	0.575474000000	-0.766711000000
C	-6.767475000000	-1.085278000000	0.857528000000
H	-8.633209000000	-1.904977000000	1.535716000000
C	-6.912741000000	0.659939000000	-0.800048000000
H	-8.893951000000	1.240977000000	-1.392137000000
C	-6.111173000000	-0.167353000000	0.012546000000
H	-6.180998000000	-1.726990000000	1.508265000000
H	-6.439493000000	1.366376000000	-1.475438000000
C	-4.627916000000	-0.076745000000	-0.020333000000
C	-3.978283000000	1.164785000000	-0.151655000000
C	-3.823765000000	-1.228027000000	0.080356000000
C	-2.579706000000	1.291917000000	-0.192836000000
H	-4.578348000000	2.070463000000	-0.190344000000
C	-2.432664000000	-1.123990000000	0.036983000000

H	-4.286147000000	-2.207334000000	0.156053000000
C	-1.785575000000	0.119354000000	-0.107820000000
H	-1.827427000000	-2.024779000000	0.087976000000
C	-0.291170000000	0.140964000000	-0.162861000000
C	0.471169000000	-0.320624000000	0.927905000000
C	0.398586000000	0.570176000000	-1.312816000000
C	1.868816000000	-0.346488000000	0.873857000000
H	-0.038086000000	-0.646065000000	1.830778000000
C	1.796191000000	0.543910000000	-1.367024000000
H	-0.164796000000	0.904941000000	-2.179171000000
C	2.558546000000	0.082093000000	-0.276311000000
H	2.432182000000	-0.681116000000	1.740246000000
H	2.305523000000	0.869164000000	-2.269895000000
C	4.053189000000	0.101255000000	-0.332400000000
C	4.844411000000	-1.073935000000	-0.261424000000
C	4.702843000000	1.344575000000	-0.464472000000
C	6.243491000000	-0.949366000000	-0.302424000000
C	6.094129000000	1.445427000000	-0.511590000000
H	4.098900000000	2.245841000000	-0.523117000000
C	6.895741000000	0.291446000000	-0.424631000000
H	6.843303000000	-1.852122000000	-0.218453000000
H	6.558050000000	2.419410000000	-0.633242000000
C	8.380029000000	0.378976000000	-0.459891000000
C	9.062859000000	1.428300000000	0.187894000000
C	9.152238000000	-0.579846000000	-1.146607000000
C	10.458186000000	1.512028000000	0.151032000000
H	8.498034000000	2.172317000000	0.741828000000
C	10.547406000000	-0.492940000000	-1.179590000000
H	8.654987000000	-1.382577000000	-1.683032000000
C	11.228433000000	0.552362000000	-0.530029000000
H	10.957375000000	2.331480000000	0.662967000000
H	11.115851000000	-1.239617000000	-1.729247000000
C	-14.808525000000	-0.670815000000	0.210700000000
H	-15.134325000000	-1.348097000000	1.012826000000
H	-15.188272000000	-1.093398000000	-0.730317000000
C	-15.465039000000	0.715115000000	0.433452000000
H	-15.087779000000	1.141861000000	1.373457000000
H	-15.141810000000	1.395882000000	-0.366642000000
C	-17.000280000000	0.644308000000	0.470859000000
H	-17.346490000000	-0.009397000000	1.282006000000
H	-17.439767000000	1.636265000000	0.629917000000
H	-17.401129000000	0.247424000000	-0.470927000000
C	12.742756000000	0.621596000000	-0.533025000000
H	13.064455000000	1.671710000000	-0.491526000000
H	13.129118000000	0.212005000000	-1.476983000000
C	13.392298000000	-0.145640000000	0.645795000000
H	13.008042000000	0.260431000000	1.593111000000
H	13.073351000000	-1.197697000000	0.608080000000
C	14.929711000000	-0.071191000000	0.637463000000
H	15.243669000000	0.984054000000	0.671830000000
H	15.309034000000	-0.475088000000	-0.314541000000
C	15.585442000000	-0.831537000000	1.805703000000
H	15.206016000000	-0.428388000000	2.756589000000
H	15.272014000000	-1.885702000000	1.771031000000
C	17.121538000000	-0.752443000000	1.791567000000

H	17.557926000000	-1.302663000000	2.633900000000
H	17.529710000000	-1.178216000000	0.865702000000
H	17.463288000000	0.288639000000	1.858300000000
C	4.240653000000	-2.469576000000	-0.218447000000
H	4.959725000000	-3.151134000000	0.254426000000
H	3.339096000000	-2.485686000000	0.401474000000
C	3.886010000000	-3.007820000000	-1.624559000000
H	3.137622000000	-2.371136000000	-2.109575000000
H	4.773674000000	-3.037146000000	-2.267663000000
H	3.477133000000	-4.023497000000	-1.557753000000
C	-1.979459000000	2.689403000000	-0.225488000000
H	-1.068459000000	2.707812000000	-0.831255000000
H	-2.692786000000	3.369676000000	-0.708861000000
C	-1.648674000000	3.227073000000	1.186652000000
H	-2.546481000000	3.252774000000	1.815566000000
H	-0.905931000000	2.591863000000	1.682233000000
H	-1.241976000000	4.244073000000	1.127477000000

Table S7. Excited state Cartesian coordinates of Sexi_Ph4

C	-12.588022000000	0.197499000000	-0.969122000000
C	-11.203197000000	0.290938000000	-0.971275000000
C	-10.418107000000	-0.355700000000	0.013241000000
C	-11.115048000000	-1.099625000000	0.995199000000
C	-12.500419000000	-1.185064000000	0.990747000000
C	-13.272339000000	-0.539800000000	0.010979000000
H	-13.155269000000	0.694342000000	-1.752988000000
H	-10.715280000000	0.838904000000	-1.770678000000
H	-10.561521000000	-1.589349000000	1.789654000000
H	-12.999757000000	-1.760092000000	1.767305000000
C	-8.958085000000	-0.263205000000	0.013095000000
C	-8.144228000000	-1.253657000000	0.622026000000
C	-8.276536000000	0.822291000000	-0.596971000000
C	-6.768057000000	-1.172071000000	0.617373000000
H	-8.611319000000	-2.119269000000	1.080504000000
C	-6.901279000000	0.916879000000	-0.591430000000
H	-8.849914000000	1.619862000000	-1.058306000000
C	-6.077488000000	-0.078301000000	0.014649000000
H	-6.203919000000	-1.955942000000	1.110309000000
H	-6.440240000000	1.763350000000	-1.088138000000
C	-4.635911000000	0.013244000000	0.016055000000
C	-3.955015000000	1.233326000000	-0.270301000000
C	-3.801042000000	-1.102669000000	0.327430000000
C	-2.579695000000	1.372218000000	-0.298160000000
H	-4.546327000000	2.133355000000	-0.404720000000
C	-2.433377000000	-0.996311000000	0.301242000000
H	-4.243096000000	-2.071726000000	0.529753000000
C	-1.750353000000	0.210523000000	-0.052344000000
H	-1.841406000000	-1.888908000000	0.474910000000
C	-0.306419000000	0.176379000000	-0.140968000000
C	0.456549000000	-0.672727000000	0.721402000000
C	0.450398000000	0.919626000000	-1.099223000000
C	1.828827000000	-0.758076000000	0.645572000000
H	-0.054990000000	-1.209765000000	1.513463000000
C	1.823484000000	0.833162000000	-1.170245000000
H	-0.068976000000	1.502981000000	-1.848986000000
C	2.577850000000	-0.015419000000	-0.310950000000
H	2.352592000000	-1.346331000000	1.390070000000

H	2.341824000000	1.377320000000	-1.953913000000
C	4.035906000000	-0.046383000000	-0.395037000000
C	4.840662000000	-1.227383000000	-0.270483000000
C	4.714817000000	1.179991000000	-0.622695000000
C	6.226074000000	-1.102896000000	-0.323148000000
C	6.091807000000	1.272376000000	-0.664357000000
H	4.124890000000	2.085907000000	-0.719981000000
C	6.897726000000	0.124110000000	-0.504164000000
H	6.818120000000	-2.006436000000	-0.208714000000
H	6.553752000000	2.238564000000	-0.840044000000
C	8.366638000000	0.201981000000	-0.537614000000
C	9.047740000000	1.368607000000	-0.130251000000
C	9.155291000000	-0.880297000000	-0.982446000000
C	10.435164000000	1.443130000000	-0.162533000000
H	8.481575000000	2.215387000000	0.244616000000
C	10.542071000000	-0.797246000000	-1.014509000000
H	8.671994000000	-1.782511000000	-1.343542000000
C	11.214913000000	0.363704000000	-0.604042000000
H	10.926844000000	2.355684000000	0.166520000000
H	11.117472000000	-1.644936000000	-1.379410000000
C	-14.780647000000	-0.601364000000	0.034740000000
H	-15.104973000000	-1.563164000000	0.452721000000
H	-15.168266000000	-0.560941000000	-0.991523000000
C	-15.423947000000	0.538517000000	0.853583000000
H	-15.035944000000	0.502858000000	1.879274000000
H	-15.099297000000	1.501298000000	0.439436000000
C	-16.953159000000	0.467310000000	0.871507000000
H	-17.301953000000	-0.474616000000	1.309975000000
H	-17.382411000000	1.285967000000	1.458017000000
H	-17.365861000000	0.531849000000	-0.141736000000
C	12.724183000000	0.433306000000	-0.598929000000
H	13.046035000000	1.466636000000	-0.780471000000
H	13.123141000000	-0.167204000000	-1.426429000000
C	13.349427000000	-0.060158000000	0.722718000000
H	12.948451000000	0.536736000000	1.552662000000
H	13.025085000000	-1.092832000000	0.907912000000
C	14.880775000000	0.009461000000	0.728480000000
H	15.199121000000	1.044697000000	0.538856000000
H	15.275622000000	-0.585569000000	-0.107626000000
C	15.509357000000	-0.480676000000	2.038861000000
H	15.114551000000	0.114199000000	2.873567000000
H	15.190958000000	-1.514862000000	2.227485000000
C	17.039283000000	-0.408974000000	2.039014000000
H	17.456804000000	-0.765157000000	2.986314000000
H	17.465045000000	-1.022297000000	1.236721000000
H	17.388052000000	0.618935000000	1.887785000000
C	4.266287000000	-2.633017000000	-0.233244000000
H	5.064295000000	-3.320928000000	0.067434000000
H	3.480861000000	-2.736502000000	0.518254000000
C	3.705864000000	-3.081535000000	-1.596290000000
H	2.882065000000	-2.437163000000	-1.915278000000
H	4.481607000000	-3.043885000000	-2.367625000000
H	3.330411000000	-4.108595000000	-1.539534000000
C	-2.027271000000	2.783438000000	-0.405104000000
H	-1.272510000000	2.872453000000	-1.190690000000
H	-2.847237000000	3.445929000000	-0.703227000000
C	-1.425809000000	3.299518000000	0.915577000000
H	-2.172112000000	3.276142000000	1.715892000000
H	-0.574506000000	2.690093000000	1.230137000000
H	-1.079356000000	4.332164000000	0.802283000000

[1] P. Guiglion and M. A. Zwijnenburg, Phys Chem Chem Phys, 2015, 17, 17854-17863.
